# A Gallbladder‐Specific Hydrophobic Bile Acid‐FXR‐MUC1 Signaling Axis Mediates Cholesterol Gallstone Formation

**DOI:** 10.1002/advs.202401956

**Published:** 2025-02-11

**Authors:** Hongtan Chen, Xin Jiang, Yiqiao Li, Honggang Guo, Jianguo Wu, Sha Li, Fengling Hu, Guoqiang Xu

**Affiliations:** ^1^ Division of Gastroenterology the First Affiliated Hospital Zhejiang University School of Medicine Hangzhou Zhejiang 310002 China; ^2^ Division of Nephrology Zhejiang Provincial People's Hospital Hangzhou Medical College Affiliated Hospital Hangzhou Zhejiang 310014 China; ^3^ Laboratory of Experimental Animal and Safety Evaluation Zhejiang Academy of Medical Sciences Medical College Hangzhou Zhejiang 310063 China; ^4^ Clinical laboratory Zhejiang Provincial People's Hospital Hangzhou Medical College Affiliated Hospital Hangzhou Zhejiang 310014 China

**Keywords:** caveolae, cholesterol gallstone, farnesoid X receptor, hydrophobic bile acid pool, protein kinase Cζ

## Abstract

Differences in the distribution of hydrophilic and hydrophobic bile acids (BA) are observed in mouse models of non‐alcoholic fatty liver disease (NAFLD) induced by a high‐fat‐cholesterol “Western‐style” diet (WD), and cholesterol gallstone disease (CGD) induced by a lithogenic diet. Despite sharing common pathological processes, these models exhibit distinct characteristics in their BA pools. The study investigates the impact of hydrophobic BA (^Hpho^BA) and hydrophilic BA (^Hphil^BA) on CGD development using cytochrome‐P450‐2c70 knockout (C70‐KO) mice (mice^C70‐KO^), genetically modified to resemble humans with a hydrophobic BA pool. All mice^C70‐KO^ fed the WD develop CGD, resembling human cholelithiasis patients, while WD‐fed wild‐type (WT) mice (mice^WT^) show cholesterol‐saturated bile but rarely form gallstones. Compared to mice^WT^, WD‐fed mice^C70‐KO^ display caveolae microdomain redistribution in the gallbladder mediated by the ^Hpho^BA, FXR, and miR30c/e axis, which enhances the Sp1 transcriptional activity of mucin‐1 (*MUC1*) genes through nuclear translocation of protein kinase Cζ (PKCζ). These changes contribute to increased production of pronucleating agents (MUC1 and MUC5ac) and accelerate crystallization of gallbladder cholesterol. The data also suggest that WD‐fed mice^C70‐KO^ appropriately model human CGD since lithogenic diet‐fed mice^WT^ have a larger BA pool that masks the negative effects of gallbladder FXR on CGD development.

## Graphical abstract

Under hydrophobic bile acid pools, a “Western‐style” diet induces NAFLD and elevated bile CSI, simultaneously activating intestinal FXR to increase circulating ceramide. Hydrophobic bile acids trigger gallbladder FXR‐miR30c/e signaling, causing SDPR suppression and caveolae instability. Together with ceramide‐activated PKCζ nuclear translocation and RBL1‐Sp1 activation, this induces MUC1·5ac hyperproduction, which combines with high CSI to promote gallstone formation.

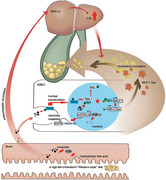

## 1 Introduction

Cholelithiasis, a common digestive disorder, primarily develops from cholesterol deposits in the gallbladder, leading to cholesterol gallstone disease (CGD).^[^ ^1^ ^]^ Non‐alcoholic fatty liver disease (NAFLD), which is induced by a high‐fat‐cholesterol “Western‐style” diet (WD), shares several risk factors with CGD.^[^ ^2^ ^]^ Factors contributing to cholesterol gallstone formation include an increased cholesterol saturation index (CSI) and accelerated cholesterol nucleation time (NT) in gallbladder bile. Elevated biliary CSI is often attributed to increased cholesterol export via the hepatic apical membrane due to upregulation of ATP‐binding cassette transporters G5/G8 (ABCG5/G8) or reduced bile acid (BA) production, which occurs through activation of the ileal farnesoid X receptor (FXR)‐fibroblast growth factor 15/19 (FGF15 in mice, FGF19 in humans) pathway. This pathway mediates a decrease in hepatic cholesterol 7 alpha‐hydroxylase (Cyp7a1), the key enzyme for BA synthesis.^[^ ^1^ ^]^ The nucleation of bile cholesterol is also largely influenced by gallbladder mucus (MUC) concentrations,^[^ ^1^ ^]^ though the mechanisms regulating its expression in CGD are not yet fully understood.

A lithogenic diet (LD) is commonly used to create murine models of CGD.^[^ ^3^ ^]^ In addition to high‐fat and high‐cholesterol components, LD contains 0.5% exogenous hydrophobic cholic acid (CA), which, in comparison to human bile, is more hydrophilic in mouse bile due to the presence of muricholic acid (MCA). This difference naturally inhibits ileal cholesterol absorption and the formation of gallbladder cholesterol crystals.^[^ ^4^ ^]^ LD rapidly alters the BA composition in mice, making it more similar to human bile, while also inhibiting Cyp7a1 expression by reducing BA synthesis and impairing gallbladder emptying via the FXR‐FGF15/19 axis.^[^ ^5^ ^]^

However, several studies suggest that mice on a WD (a high‐fat, high‐cholesterol diet without CA) also exhibit intestinal activation of the FXR‐FGF15 pathway and increased hepatic ABCG5/G8 expression.^[^ ^6^ ^]^ In mice fed a WD, taurine‐conjugated β‐muricholic acid (TβMCA), a natural FXR antagonist, is converted to βMCA by bile salt hydrolase (BSH) produced by gut microbes, reducing FXR activity in the ileum and thereby managing CGD risk (e.g., elevated serum ceramide and FGF15 levels).^[^ ^7^ ^]^ Treatments with BSH‐resistant glycine‐conjugated βMCA (GlyMCA) or BSH inhibitors can reverse these effects.^[^ ^7^, ^8^ ^]^

Knockout of ABCG5/G8 (ABCG5/G8‐KO) does not completely prevent CGD in LD‐fed mice, whereas reducing gallbladder MUC1 levels significantly decreases the incidence of CGD, regardless of changes in biliary CSI.^[^ ^9^ ^]^ This suggests that the upregulation of ABCG5/G8 and the activation of the FXR‐FGF15/19‐Cyp7a1 signaling pathway may not be the primary drivers of CGD pathogenesis. Therefore, we explored the potential roles of hydrophobic BAs (e.g., CA, chenodeoxycholic acid [CDCA], and deoxycholic acid [DCA]) and hydrophilic BAs (e.g., αMCA, βMCA, and ursodeoxycholic acid [UDCA]) in CGD. Recent studies have shown that mice lacking the cytochrome P450 2c70 (Cyp2c70) gene no longer produce hydrophilic MCA and exhibit BA compositions similar to those in humans.^[^ ^10^ ^]^ As a result, we also investigated the use of Cyp2c70 knockout (C70‐KO) mice (mice^C70‐KO^) as a promising model for studying CGD.

## 2 Results

### 2.1 Difference in Gallbladder Mucus Contents Between Wild‐Type Mice (mice^WT^) and Cytochrome‐P450‐2c70 Knockout Mice (mice^C70‐KO^) after WD

**Figure 1:**
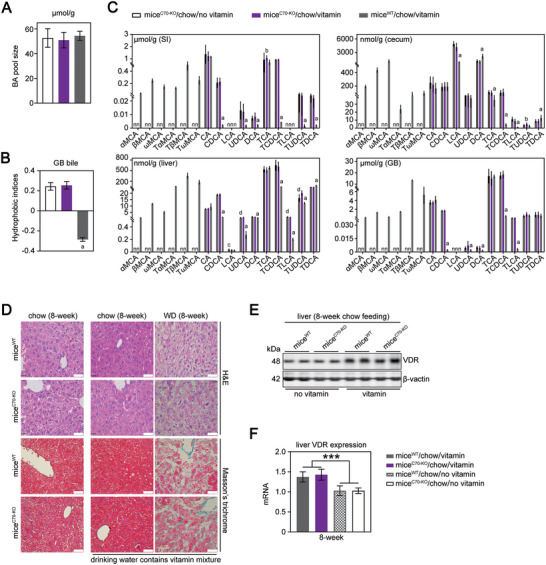
BA composition is altered in mice^C70‐KO^. A,B) BA pool size (A) and Hydrophobic indices (B). GB, gallbladder. C) BA profile in the SI, cecum, liver and GB. n, no data. SI, small intestine, GB, gallbladder. D) Histological examination of liver by H&E and Masson's trichrome staining (scale bar: 100 µm). E) WB analysis of vitamin D receptor (VDR) expression in liver tissue lysates. The expression of βactin was used as a loading internal control. F) Expression of VDR mRNA analysis by qRT‐PCR in liver tissues. Expression data were normalized to the expression of 18s RNA. chow, fed on chow diet; no vitamin, drinking water contains no vitamin mixture; vitamin, drinking water contains vitamin mixture (Table S3, Supporting Information). mice^C70‐KO^/chow/no vitamin, chow‐fed mice^C70‐KO^ on normal drinking water contains no vitamin mixture; mice^C70‐KO^/chow/vitamin, chow‐fed mice^C70‐KO^ on drinking water contains vitamin mixture; mice^WT^/chow/vitamin, chow‐fed mice^WT^ on drinking water contains vitamin mixture. Each experimental group comprised 10 mice. The data is represented as mean ± standard deviation. Normality of the data was assessed using the Shapiro‐Wilkes test. Group comparisons were performed using analysis of variance (ANOVA) along with Tukey's range test for normally distributed data. In cases of non‐normally distributed datasets, Kruskal‐Wallis non‐parametric ANOVA with Benjamini‐Hochberg corrections was utilized to compare more than two samples. An adjusted *p* < 0.05 was considered significant after Tukey's or Benjamini‐Hochberg corrections for multiple comparisons. Individual *p* values were provided in Primary Data for Figure 1. Data with different lowercase letter (a, mice^WT^/chow/vitamin versus other mice; b, mice^WT^/chow/vitamin versus mice^C70‐KO^/chow/vitamin; c, mice^C70‐KO^/chow/no vitamin versus other mice; d, mice^C70‐KO^/chow/no vitamin versus mice^C70‐KO^/chow/ vitamin) indicates significant differences (*p < *0.05). ***, significant difference between mice^WT^/chow/vitamin or mice^C70‐KO^/chow/vitamin versus mice^WT^/chow/no vitamin or mice^C70‐KO^/chow/no vitamin (*P < *0.001).

**Figure 2:**
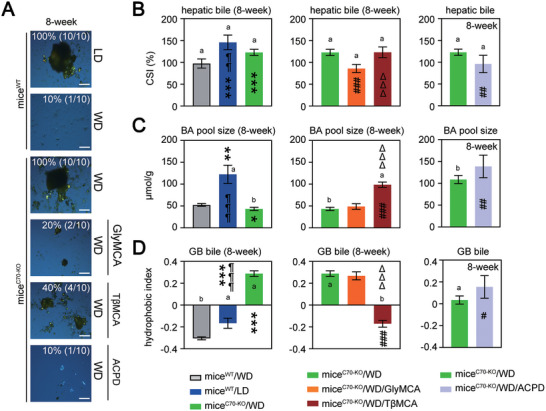
The CGD model of mice^C70‐KO^. A) The polarizing light microscopy examination of cholesterol crystals (scale bar: 100 µm). B–D) CSI values (B), BA pool size (C) and the hydrophobic indices (D) of the GB bile. GB, gallbladder; BA, bile acid. WD, fed on western‐style diet; LD, fed on lithogenic diet. mice^WT^/WD, WD‐fed mice^WT^; mice^WT^/LD, LD‐fed mice^WT^; mice^C70‐KO^/WD, WD‐fed mice^C70‐KO^; mice^C70‐KO^/WD/GlyMCA, GlyMCA‐treated (50 mg kg^−1^ per day) WD‐fed mice^C70‐KO^; mice^C70‐KO^/WD/TβMCA, TβMCA‐treated (500 mg kg^−1^ per day) WD‐fed mice^C70‐KO^; mice^C70‐KO^/WD/ACPD, ACPD‐treated (10 mg kg^−1^ per day) WD‐fed mice^C70‐KO^. Each experimental group comprised 10 mice. The data is presented as mean ± standard deviation. Data normality was determined by using Shapiro‐Wilkes test. Differences (*p* < 0.05 considered significant) between two independent samples with normal distribution were determined by using Student's t‐test, while Mann‐Whitney test was used to compare non‐normal data (*p* < 0.05 considered significant). For more than two normally distributed samples, statistical comparisons were made by ANOVA with Tukey's range test. And Kruskal‐Wallis non‐parametric ANOVA with Benjamini‐Hochberg corrections was used to compare more than two samples with non‐normal distribution. An adjusted *p* < 0.05 was considered significant after Tukey's or Benjamini‐Hochberg corrections for multiple comparisons. Data of chow‐fed mice^WT^ and mice^C70‐KO^ and individual *p* values were provided in Primary Data for Figure 2. Data with a lowercase “a” indicates a significant increase, while data with a lowercase “b” indicates a significant reduction, both compared to the control (WD vs chow, *P *< 0.05). *p < 0.05, **p < 0.01, ***p < 0.001; significant difference between mice^WT^/WD vs mice^WT^/LD or mice^C70‐KO^/WD. ¶¶¶*P < *0.001; significant difference between mice^WT^/LD vs mice^C70‐KO^/WD. ^#^ *p < *0.05, ^##^ *p < *0.01, ^###^ *p < *0.001; significant difference between mice^C70‐KO^/WD vs mice^C70‐KO^/WD/GlyMCA, mice^C70‐KO^/WD/TβMCA or mice^C70‐KO^/WD/ACPD. ΔΔ*P<*0.01, ΔΔΔ*P < *0.001; significant difference between mice^C70‐KO^/WD/GlyMCA vs mice^C70‐KO^/WD/TβMCA.

We found mice^C70‐KO^ presented an increase in BA hydrophobic indices without any alterations to BA pool size, biliary CSI, and hepatic and serum lipid concentrations (**Figure** **1**A–C; Tables S1A and S2A, Supporting Information). The WD did not aggravate the metabolic abnormalities or hepatic injury (serum alanine aminotransferase (ALT), aspartate aminotransferase (AST), hematoxylin‐eosin (H&E), and Masson's trichrome staining) in mice^C70‐KO^ compared to wild‐type mice (mice^WT^; Figure 1D; Table S2A, Supporting Information). CGD is often asymptomatic over decades. We found that 100% (10/10) of WD‐fed mice^C70‐KO^ developed CGD accompanied by an elevation of biliary CSI and BA hydrophobic indices and a reduction of BA pool size (**Figure** **2**A,B–D left panels; and Table S1A, Supporting Information). After 8 weeks of the WD, mice^WT^ showed a 10% (1/10) incidence of CGD (Figure 2A), although they had supersaturated bile (average biliary CSI = 100.7) (Figure 2B–D left panels; Table S1A, Supporting Information).

The similarity of supersaturated bile cholesterol levels and the obviously different frequency of cholesterol crystal formation between mice^C70‐KO^ and mice^WT^ on a WD may be attributed to the recruitment of distinct expression patterns of lithogenesis genes. After the WD, principal components analysis (PCA) of the candidate lithogenic genes revealed that the genes responsible for BA metabolism (BA‐related signaling and transport and BA/bilirubin conjugation) and cholesterol nucleation from mice^C70‐KO^ were grouped separately from those of mice^WT^ (Figure S1A–D, Supporting Information). The gallbladder hypomotility and MUC contents elevation contribute to the process of solid gallstone formation.^[^ ^1^ ^]^ When fed a WD, both mice^WT^ and mice^C70‐KO^ exhibited decreased gallbladder contraction and reduced expression of the cholecystokinin A receptor (CCKAR) (Figure S2A, Supporting Information). However, WD‐fed mice^C70‐KO^ displayed a shorter NT for cholesterol crystallization in gallbladder bile compared to WD‐fed mice^WT^ (Figure S3A,B, Supporting Information).

**Figure 3:**
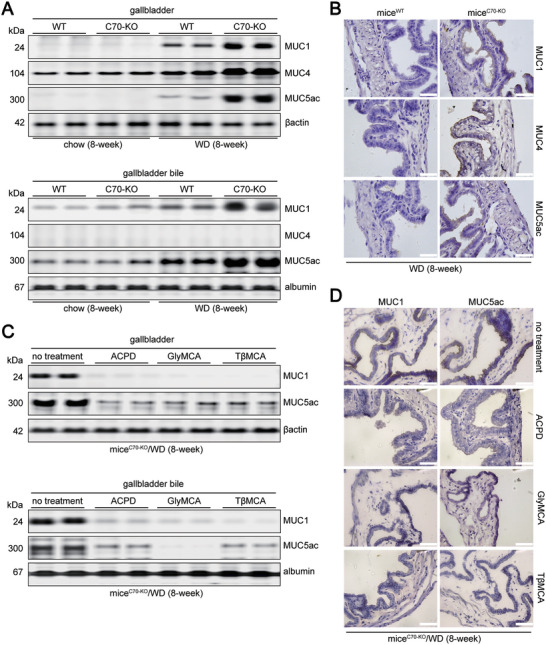
The difference of the expression of MUC1, MUC4, and MUC5ac in the gallbladder tissues and gallbladder bile between mice^C70‐KO^ and mice^WT^. A) WB analysis of MUC1, MUC4, and MUC5ac expression in gallbladder tissues (upper) and bile (lower) collected from chow‐fed or WD‐fed mice^C70‐KO^ and mice^WT^. The expression of βactin or albumin was used as a loading internal control for cytoplasmic or bile extracts, respectively. B) Immunohistochemical staining of MUC1, MUC4 and MUC5ac expression in gallbladder tissues collected from mice^C70‐KO^ and mice^WT^ after 8‐week WD feeding (scale bar: 100 µm). C) WB analysis of MUC1 and MUC5ac expression in gallbladder tissues (upper) and bile (lower) collected from mice^C70‐KO^ after 8‐week WD feeding, with or without the treatment of ACPD, GlyMCA or TβMCA. The expression of βactin or albumin was used as a loading internal control for cytoplasmic or bile extracts, respectively. D) Immunohistochemical staining of MUC1 and MUC5ac expression in GB tissues collected from mice^C70‐KO^ mice^C70‐KO^ after 8‐week WD feeding, with or without the treatment of ACPD, GlyMCA or TβMCA (scale bar: 100 µm). chow, fed on chow diet; WD, fed on western‐style diet. Each experimental group comprised 10 mice. ACPD, under ACPD (10 mg k^−1^g/day) treatment; GlyMCA, under GlyMCA (50 mg k^−1^g/day) treatment; TβMCA, under TβMCA (500 mg k^−1^g/day) treatment.

In normal mouse gallbladders, the expression of epithelial MUC1 and MUC3 is higher compared with epithelial MUC4 and gel‐forming MUC5ac, MUC5b, and MUC6.^[^ ^9a^ ^]^ Quantitative real‐time polymerase chain reaction (qRT‐PCR) and western blot (WB) analyses showed that the gallbladder's epithelial MUC1, MUC4, and gel‐forming MUC5ac expression (**Figure** **3**A,B; Figure S1C, Supporting Information) were more increased in mice^C70‐KO^ than in mice^WT^ after the WD. The down‐regulation of MUC1 (sgMUC1) and MUC5ac (sgMUC5ac) but not MUC4 (sgMUC4) in the gallbladders of WD‐fed mice^C70‐KO^ had a biliary CSI‐independent effect on the prevention of growth and agglomeration of gallbladder cholesterol crystals (Figures S4A–C and S5A and Tables S1B–D, Supporting Information). The gallbladder MUC1 deficiency also reduced gallbladder MUC5ac expression in WD‐fed micec^C70‐KO^ (Figure S5B, Supporting Information), which was consistent with prior studies.^[^ ^9^, ^11^ ^]^ Additionally, overexpression of MUC1 (MUC1‐OE) after intraperitoneal (i.p.) injection of adeno‐associated viral vector (AAV) increased gallbladder MUC1 and MUC5ac (MUC1·5ac) levels and CGD susceptibility in mice^WT^ fed either a WD for 8 weeks or a LD for 3 weeks (Figures S4D and S5C,D; Tables S1E,F, Supporting Information).

### 2.2 Differential Benefits of GlyMCA and TβMCA on Treatment of CGD

A thin layer of mucin gel in the gallbladder appears to be important for reducing the susceptibility to CGD in LD‐fed gallstone‐resistant AKR/J mice.^[^ ^12^ ^]^ Periodic acid‐Schiff (PAS) staining showed that the deposition of mucin was significantly higher in the gallbladder epithelium of WD‐fed mice^C70‐KO^ and LD‐fed mice^WT^ as compared to WD‐fed mice^WT^ (Figure 6A). However, histologic analyses showed that the gallbladder wall thickness, epithelial hyperplasia, inflammatory cell infiltration, and muscle hypertrophy were similarly increased in all the three groups (Figure S6B, Supporting Information).

**Figure 4:**
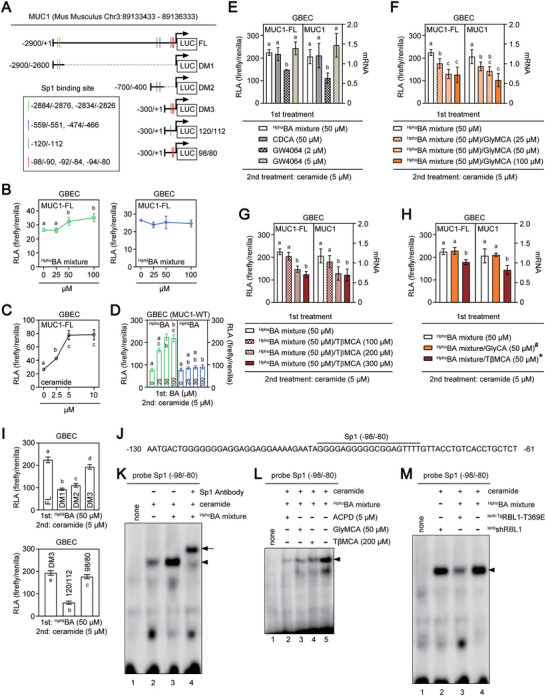
MUC1 promoter segment at −120/−80 is important for ^Hpho^BA/ceramide‐induced MUC1 gene transactivation. A) a full length (FL) and a series of deletion mutant (DM) constructs of the murine MUC1 gene promoter were transfected transiently into the human gallbladder epithelial cells (HIBEpiC). The transcription start site (+1) is indicated by the arrow. FL, LUC‐MUC1‐FL; the luciferase plasmid had the full promoter region (nucleotides −2900 to +1 bp). DM1, LUC‐MUC1‐DM1; the luciferase plasmid had a deletion fragments of the murine MUC1 promoter (nucleotides −2900 to ‐2600 bp), which containing two putative Sp1 binding sites (located at nucleotides −2884 to −2876 bp and nucleotides −2834 to −2826 bp in the MUC1 promoter). DM2, LUC‐MUC1‐DM2; the luciferase plasmid had a deletion fragments of the murine MUC1 promoter (nucleotides −700 to ‐400 bp), which containing two Sp1 binding sites (located at nucleotides −559 to −551 bp and nucleotides −474 to −466 bp in the MUC1 promoter). DM3, LUC‐MUC1‐DM3; the luciferase plasmid had 5′‐deletion fragments of the murine MUC1 promoter (nucleotides −300 to +1 bp), which containing four Sp1 binding site (located at nucleotides −120 to −112 bp, nucleotides −98 to −90 bp, nucleotides −92 to −84 bp, and nucleotides −94 to −80 bp in the MUC1 promoter). 120/112, LUC‐MUC1‐120/112; deleted 19 nucleotides, which span from bp −98 to −80 in LUC‐MUC1‐DM3. 98/80, LUC‐MUC1‐98/80; deleted 8 nucleotides, which span from bp −120 to −112 in LUC‐MUC1‐DM3. B, GBEC were transduced with LUC‐MUC1‐FL. After 24 h following lentiviral transduction, the cells were treated for an additional 24 h with various doses (0, 25, 50, 100 µm) of ^Hpho^BA mixture (left panel) or ^Hphil^BA mixture (right panel). RLA was normalized to β‐galactosidase to correct for transfection efficiency. The compositions of either ^Hphil^BA or ^Hpho^BA mixture used in these in vitro studies were shown in Table S4 (Supporting Information). C,D) GBEC were transduced with LUC‐MUC1‐FL. After 24 h following lentiviral transduction, the cells were treated for an additional 2 h with various doses (0, 2.5, 5, and 10 µm) of ceramide (C). 24 h post‐transduced GBEC (D) also were in vitro cultured with various doses (0, 25, 50, 100 µm) of ^Hpho^BA mixture (left panel) or ^Hphil^BA mixture (right panel) for 24 h prior to 2 h treatment of 5 µm ceramide. RLA was normalized to β‐galactosidase to correct for transfection efficiency. The compositions of either ^Hphil^BA or ^Hpho^BA mixture used in these in vitro studies were shown in Table S4 (Supporting Information). E) Left panel: GBEC were transduced with LUC‐MUC1‐FL. After 24 h following lentiviral transduction, the cells were treated for an additional 24 h with different compounds, such as 50 µm ^Hpho^BA mixture, 50 µm CDCA, 2 µm GW4064 or 5 µm GW4064. The cells were then stimulated with 5 µm ceramide for 2 h. RLA was normalized to β‐galactosidase to correct for transfection efficiency. Right panel: GBEC were treated with various compounds for 24 h, then stimulated with ceramide for 2 h. The compounds used in the treatment were: 50 µm ^Hpho^BA mixture, 50 µm CDCA, 2 µm GW4064 or 5 µm GW4064. The mRNA levels of MUC1 in GBEC were measured by qRT‐PCR. The MUC1 mRNA expression data was normalized to the expression of 18s RNA. F) Left panel: GBEC were transduced with LUC‐MUC1‐FL. After 24 h following lentiviral transduction, the cells were treated for 24 h with 50 µm ^Hpho^BA mixture, 50 µm ^Hpho^BA mixture plus 25 µm GlyMCA, 50 µm ^Hpho^BA mixture plus 50 µm GlyMCA or 50 µm ^Hpho^BA mixture plus 100 µm GlyMCA. Then the cells were stimulated with 5 µm ceramide for 2 h. RLA was normalized to β‐galactosidase to correct for transfection efficiency. Right panel: GBEC were treated with 50 µm ^Hpho^BA mixture, 50 µm ^Hpho^BA mixture plus 25 µm GlyMCA, 50 µm ^Hpho^BA mixture plus 50 µm GlyMCA or 50 µm ^Hpho^BA mixture plus 100 µm GlyMCA for 24 h. Then the cells were stimulated with 5 µm ceramide for 2 h. The mRNA levels of MUC1 in GBEC were measured by qRT‐PCR. The MUC1 mRNA expression data was normalized to the expression of 18s RNA. G) Left panel: GBEC were transduced with LUC‐MUC1‐FL. After 24 h following lentiviral transduction, the cells were treated for 24 h with 50 µm ^Hpho^BA mixture, 50 µm ^Hpho^BA mixture plus 100 µm TβMCA, 50 µm ^Hpho^BA mixture plus 200 µm TβMCA or 50 µm ^Hpho^BA mixture plus 300 µm TβMCA. Then the cells were stimulated with 5 µm ceramide for 2 h. RLA was normalized to β‐galactosidase to correct for transfection efficiency. Right panel: GBEC were treated with 50 µm ^Hpho^BA mixture, 50 µM ^Hpho^BA mixture plus 100 µm TβMCA, 50 µm ^Hpho^BA mixture plus 200 µm TβMCA or 50 µm ^Hpho^BA mixture plus 300 µm TβMCA for 24 h. Then the cells were stimulated with 5 µm ceramide for 2 h. The mRNA levels of MUC1 in GBEC were measured by qRT‐PCR. The MUC1 mRNA expression data was normalized to the expression of 18s RNA. The compositions of ^Hpho^BA mixture used in these in vitro studies were shown in Table S4 (Supporting Information). G) Left panel: GBEC were transduced with LUC‐MUC1‐FL. After 24 h following lentiviral transduction, the cells were treated for 24 h with 50 µm ^Hpho^BA mixture, 50 µm GlyMCA (#) or 50 µm TβMCA (*). Then the cells were stimulated with 5 µm ceramide for 2 h. RLA was normalized to β‐galactosidase to correct for transfection efficiency. Right panel: GBEC were treated with 50 µm ^Hpho^BA mixture, 50 µm GlyMCA (#) or 50 µm TβMCA (*) for 24 h. Then the cells were stimulated with 5 µm ceramide for 2 h. The mRNA levels of MUC1 in GBEC were measured by qRT‐PCR. The MUC1 mRNA expression data was normalized to the expression of 18s RNA. The compositions of ^Hpho^BA mixture used in these in vitro studies were shown in Table S4 (Supporting Information). #, BA mixture collected from GlyMCA‐treated WD‐fed mice^C70‐KO^ (as shown in Table S4, Supporting Information). *, BA mixture collected from TβMCA‐treated WD‐fed mice^C70‐KO^ (as shown in Table S4, Supporting Information). H) Upper panel: GBEC were transduced with LUC‐MUC1‐FL, LUC‐MUC1‐DM1, LUC‐MUC1‐DM2, or LUC‐MUC1‐DM3, respectively. After 24 h following transfection, the cells were treated for 24 h with 50 µm ^Hpho^BA mixture. Then the cells were stimulated with 5 µm ceramide for 2 h. RLA was normalized to β‐galactosidase to correct for transfection efficiency. Lower panel: GBEC were transduced with LUC‐MUC1‐DM3, LUC‐MUC1‐120/112, or LUC‐MUC1‐98/80, respectively. After 24 h following transfection, the cells were treated for 24‐hour with 50 µm ^Hpho^BA mixture. Then the cells were stimulated with 5 µm ceramide for 2 h. RLA was normalized to β‐galactosidase to correct for transfection efficiency. The compositions of ^Hpho^BA mixture used in these in vitro studies were shown in Table S4 (Supporting Information). I) The sequence of the proximal promoter of the mouse MUC1 gene containing the identified Sp1 binding sites (nucleotides −98 to ‐80 bp). J) lane 2–4, GBEC were treated for 2 h with 5 µm ceramide with or without pretreatment for 24 h with 50 µm ^Hpho^BA mixture. Then GBEC nuclear extracts were prepared and used to generate complexes with the MUC1 Sp1 binding site oligonucleotide (the proximal Sp1 site at −98/−80) as probes. lane 1, “none” means no nuclear extract. The compositions of ^Hpho^BA mixture used in these in vitro studies were shown in Table S4 (Supporting Information). Arrowhead, the appearance of a supershifted protein‐DNA complex. Arrow, the formation of the Sp1‐DNA complex was confirmed by addition of Sp1 antibody. K) lane 2–5, GBEC were treated for 2 h with 5 µm ceramide with or without pretreatment for 24 h with 50 µm ^Hpho^BA mixture. Then GBEC nuclear extracts were prepared and used to generate complexes with the MUC1 Sp1 binding site oligonucleotide (the proximal Sp1 site at −98/−80) as probes. lane 1, “none” means no nuclear extract; lane 2, GBEC were treated for 2 h with 5 µm ceramide and 5 µm ACPD with pretreatment for 24 h with 50 µm ^Hpho^BA mixture; lane 3, GBEC were treated for 2 h with 5 µm ceramide with pretreatment for 24 h with 50 µm ^Hpho^BA mixture and 50 µm GlyMCA; lane 4, GBEC were treated for 2 h with 5 µm ceramide with pretreatment for 24 h with 50 µm ^Hpho^BA mixture and 200 µm TβMCA. The compositions of ^Hpho^BA mixture used in these in vitro studies were shown in Table S4 (Supporting Information). L) lane 2–4, GBEC were treated for 2 h with 5 µm ceramide with or without pretreatment for 24 h with 50 µm ^Hpho^BA mixture. Then GBEC nuclear extracts were prepared and used to generate complexes with the MUC1 Sp1 binding site oligonucleotide (the proximal Sp1 site at −98/−80) as probes. lane 1, “none” means no nuclear extract; lane 2, GBEC expressing lentivirus carrying shRBL1 (^lenti^shRBL1); lane 3, GBEC expressing lentivirus carrying RBL1‐T369E (^lenti‐Tg^RBL1‐T369E). The compositions of ^Hpho^BA mixture used in these in vitro studies were shown in Table S4 (Supporting Information). Arrowhead, the appearance of a supershifted protein‐DNA complex. GBEC, gallbladder epithelial cells (HIBEpiC). RLA, Relative luciferase activity. The data for each cell experiment group is derived from 5 independent experiments. Group comparisons were performed using ANOVA along with Tukey's range test for normally distributed data. In cases of non‐normally distributed datasets, Kruskal‐Wallis non‐parametric ANOVA with Benjamini‐Hochberg corrections was utilized to compare more than two samples. An adjusted *p* < 0.05 was considered significant after Tukey's or Benjamini‐Hochberg corrections for multiple comparisons. An adjusted *p* < 0.05 was considered significant after Tukey's or Benjamini‐Hochberg corrections for multiple comparisons. Individual *p* values were provided in Primary Data for Figure 4. Data with different lowercase letter indicates significant differences (*P < *0.05) between each assigned GBEC group.

Our data also showed a function of MUC1 or MUC5ac in CGD prevention that was independent of bile CSI (Figure S5A–D; Table S1B–F, Supporting Information). Additionally, previous studies^[^ ^9^, ^11^, ^13^ ^]^ and our results (Figure S5B,D, Supporting Information) indicate that epithelial MUC1 seems to play a key role in the up‐regulation of the gel‐forming MUC5ac during cholesterol gallstone formation. Therefore, our work has begun to focus on exploring the specific reasons for the increased expression of MUC1 during the pathogenesis of CGD. After observing the unique hydrophobic characteristics of gallbladder bile in mice^C70‐KO^ and mice^WT^ (Figure 2C left panel; Figure 1B), we explored whether hydrophobic BA (^Hpho^BA) and hydrophilic BA (^Hphil^BA) have different impacts on the expression of MUC1. We used an in vitro human gallbladder epithelial cell (GBEC) model with cultured human intrahepatic biliary epithelial cells (HIBEpiC) expressing mouse MUC1 full‐length promoter (MUC1‐FL) luciferase reporter (Figure 4A). MUC1‐FL transactivation mildly increased (Figure 4B) with treatment of the ^Hpho^BA mixture (a reconstituted BA mixture from the gallbladders of mice^C70‐KO^; Table S4, but not the ^Hphil^BA mixture (a reconstituted BA mixture from the gallbladders of mice^WT^; Table S4, Supporting Information).

Lipid peroxidation has been implicated as a potential mechanism for the hyper‐production of gallbladder MUC.^[^ ^14^ ^]^ Ceramide‐metabolism abnormality exacerbates endogenous lipid peroxidation.^[^ ^15^ ^]^ A WD induces the accumulation of gallbladder 4‐hydroxy‐2‐trans‐nonenal (4‐HNE) (a lipid peroxidation marker)^[^ ^16^ ^]^ and the elevation of circulating ceramide levels in both mice^C70‐KO^ and mice^WT^ (Figures S6C and S7A; Table S5A, Supporting Information). Similar changes in 4‐HNE levels were observed in vitro in GBECs treated with ^C16:0^ceramide (which majorly contributes to NAFLD progression)^[^ ^17^ ^]^ (Figure S6D, Supporting Information). But in comparison with the up‐regulated effects of stimulation with^C16:0^ceramide alone, the reconstituted ^Hpho^BA mixture could work together with ^C16:0^ceramide supplements to further increase the luciferase activities of MUC1‐FL constructs in vitro (Figure 4C,D). Taking into account the potency of ^Hpho^BA as FXR agonists,^[^ ^18^ ^]^ we found that FXR agonists CDCA and GW4064 could also strengthen the effects of ^C16:0^ceramide on MUC1‐FL transactivation and MUC1 mRNA expression in vitro (Figure 4E). Conversely, administering FXR antagonists such as GlyMCA or TβMCA to GBECs negatively impacted the MUC1 gene transactivation/expression stimulated by a mixture of ^Hpho^BA and ceramide (Figure 4F–H).

Prior studies revealed that oral TβMCA or UDCA could help prevent CGD, although LD‐fed mice have cholesterol saturated bile. TβMCA treatment was more effective than UDCA upon feeding with the LD.^[^ ^19^ ^]^ We evaluated the effects of GlyMCA and TβMCA on CGD prevention. GlyMCA treatment (50 mg kg^−1^ per day) greatly reduced the occurrence of cholesterol gallstone formation (2/10, 8 weeks) in WD‐fed mice^C70‐KO^. This occurred through the alleviation of risk factors contributing to CGD susceptibility (Figures 2A,B–D middle panels, 3C,D; Figures S2B, S3C, and S7B; Tables S1G, S2B, and S5B, Supporting Information).

Oral TβMCA (500 mg kg^−1^) reduced the CGD incidence to 40% (4/10, 8 weeks) in WD‐fed mice^C70‐KO^, which might be associated with the elevated BA pool size, increased gallbladder BA hydrophilicity, reduced gallbladder MUC1·5ac contents, and prolonged cholesterol NT in the gallbladder bile (Figures 2A,C,D middle panels, 3C,D; Figure S3C, Supporting Information). However, it had a weaker influence on the WD‐induced elevation in biliary CSI, impaired gallbladder contraction, accumulated serum ceramide levels, and metabolic dysfunction than GlyMCA treatment (Figure 2B middle panel; Figures S2B and S7B; Tables S1G, S2B, and S5B, Supporting Information). The clustering of PCA results showed that except for genes directly linked to cholesterol nucleation (*MUC1* and *MUC5ac*), the expression patterns of other genes involved in cholesterol gallstone formation and NAFLD progression were significantly different between mice^C70‐KO^ on the WD with dietary GlyMCA and TβMCA (Figure S8A–D, Supporting Information). The similar ratio of TβMCA to natural FXR agonists (CA, CDCA, DCA, and their taurine‐conjugated counterparts) in the intestine and the increased activities of gut BSH presented in both mice^WT^ and TβMCA‐treated mice^C70‐KO^ after the WD (Figure S9A left panel and B, Supporting Information). These findings indicated that in comparison with BSH‐resistant GlyMCA, oral TβMCA is insufficient to alleviate gut FXR‐associated metabolic abnormalities^[^ ^7^, ^8^ ^]^ and bile cholesterol supersaturation after a WD.

### 2.3 Activation of Nuclear PKCζ‐RBL1‐Sp1 Axis Promotes Gallbladder MUC1 Production

The transactivation of the MUC1 gene was demonstrated to be regulated by the transcription factor Sp1, which is also a potential risk factor in the progression of NAFLD.^[^ ^20^ ^]^ We analyzed MUC1 promoter sequences from various species using the JASPAR transcription factor binding profile server^[^ ^21^ ^]^ and identified eight potential Sp1 consensus binding sites within the mouse MUC1 promoter region (**Figure** **4**A; Table S6, Supporting Information). A detailed promoter analysis using deletion mutants (DMs) showed that the most proximal Sp1 binding site (‐98/‐80) provided the strongest transcriptional activation for the MUC1 promoter (Figure 4A,I).

**Figure 5:**
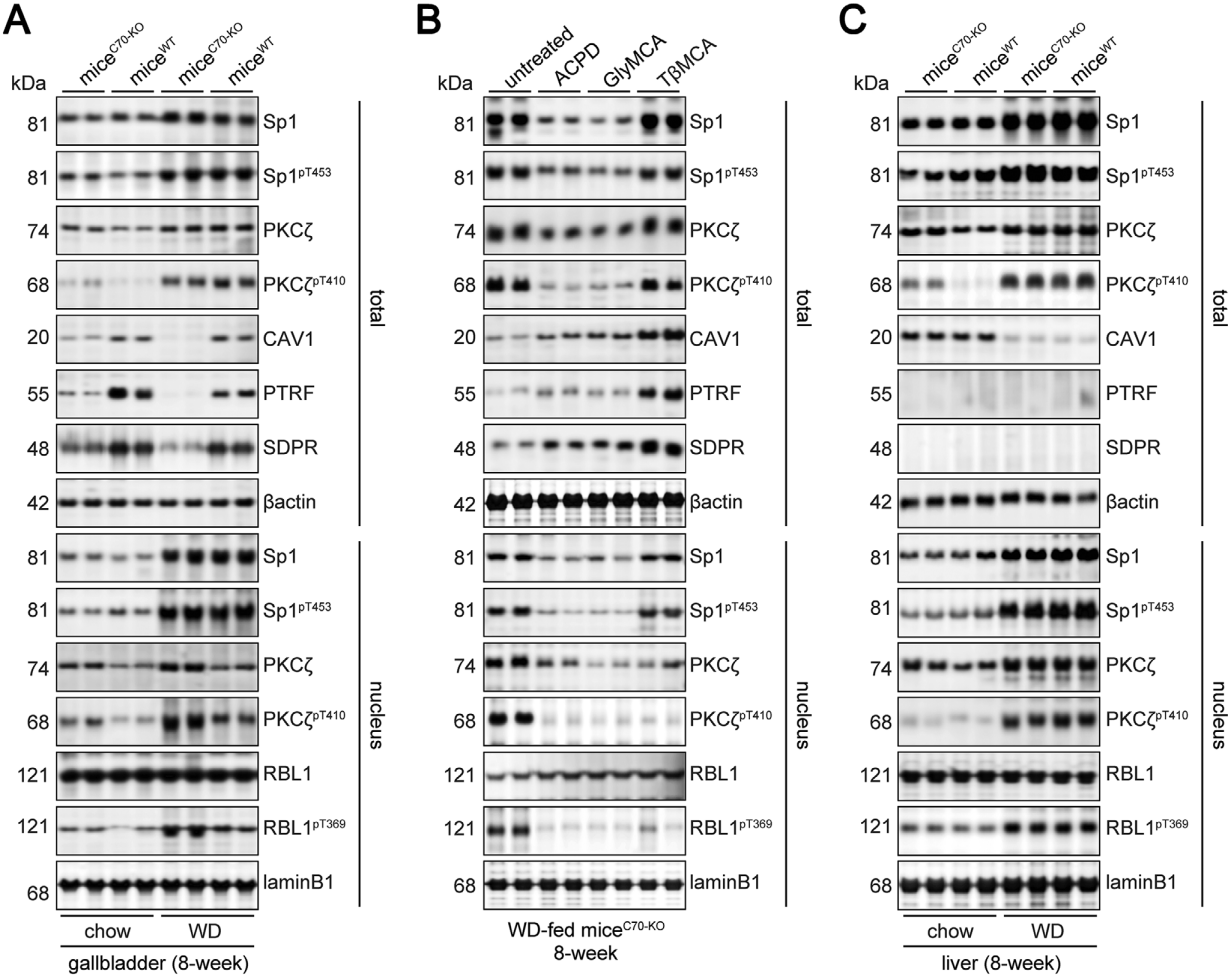
The difference of the activity of PKCζ‐RBL1‐Sp1 axis and the expression of caveolae constituents in the gallbladder and liver between mice^C70‐KO^ and mice^WT^. A–C) WB analysis of Sp1 and its phosphorylated form pT453, PKCζ and its phosphorylated form pT410, RBL1 and its phosphorylated form pT369, CAV1, PTRF, and SDPR expression in gallbladder lysates (A and B) or liver lysates (C) collected from chow‐fed or WD‐fed mice^WT^ and mice^C70‐KO^, without (untreated) or with ACPD, GlyMCA, TβMCA treatment. The expression of βactin or laminB1 was used as a loading internal control for total or nuclear extracts, respectively. Each experimental group comprised 10 mice. WD, fed on western‐style diet.

To further validate this, we performed an electrophoretic mobility shift assay (EMSA) using a 19‐bp radiolabeled probe corresponding to nucleotides ‐98 to ‐80 of the MUC1 promoter. The results revealed that pre‐treatment with ^Hpho^BA significantly enhanced Sp1 binding to this DNA region in vitro under ceramide‐induced conditions (Figure 4J,K). Moreover, the deletion of Sp1 greatly reduced the transactivation of the MUC1 gene in vitro when stimulated by a mixture of ^Hpho^BA and ceramide (Figure S10A,B, Supporting Information). Interestingly, we observed that total Sp1 expression and Sp1 phosphorylation at threonine 453 (Sp1^pT453^), a marker of transcriptional activation,^[^ ^22^ ^]^ were elevated in the gallbladders of both WD‐fed mice^WT^ and mice^C70‐KO^ (**Figure** **5**A). However, no significant difference in the nuclear Sp1^pT453^ levels between mice^WT^ and mice^C70‐KO^ was observed (Figure 5A), suggesting that Sp1 activation alone was not the primary differentiating factor between these groups.

It has been reported that the DNA‐binding capacity of Sp1 is hindered by the presence of retinoblastoma‐like 1 (RBL1) associated with chromatin, but the activation of atypical protein kinase Cζ (PKCζ) through phosphorylation at threonine 410 (PKCζ^pT410^) leads to the dissociation of RBL1 from the motifs that Sp1 targets.^[^ ^23^ ^]^ Additionally, phosphorylation of RBL1, such as the phosphorylation of PBL1 at threonine 369 (PBL1^pT369^), has been shown to reduce its chromatin‐repressive activity.^[^ ^24^ ^]^

Based on these prior findings, we sought to further investigate the mechanism by which PKCζ‐mediated phosphorylation of RBL1 may facilitate Sp1‐driven MUC1 expression. To examine this, we utilized a MUC1‐FL promoter luciferase assay (Figure S10C left panel, Supporting Information), qRT‐PCR (Figure S10C right panel, Supporting Information), and EMSA (Figure 4J,L), showing that inhibition of PKCζ using 2‐acetyl‐1,3‐cyclopentanedione (ACPD)^[^ ^25^ ^]^ reduced the protein‐DNA complex formation at the Sp1 binding site (‐98/‐80) on the MUC1 promoter. This led to decreased MUC1 gene transactivation and expression in GBECs following stimulation with the ^Hpho^BA‐ceramide mixture (Figure S11A,B, Supporting Information). Moreover, mutation of threonine 369 in RBL1 to glutamic acid (RBL1‐T369E) disrupted Sp1 binding to the MUC1 promoter (Figure 4J,M) and reduced the MUC1 transactivation induced by the ^Hpho^BA‐ceramide mixture (Figure S10D–F, Supporting Information). Notably, while both WD‐fed mice^WT^ and mice^C70‐KO^ exhibited elevated total PKCζ^pT410^ levels, the nuclear levels of PKCζ, PKCζ^pT410^, and RBL1^pT369^ were specifically increased in the gallbladder of mice^C70‐KO^ (Figure 5A). These results confirm that PKCζ‐mediated phosphorylation of RBL1 at threonine 369 facilitates Sp1‐driven MUC1 expression, providing a mechanistic explanation for the observed differences between the two mouse models.

ACPD treatment had similar effects to oral GlyMCA on the prevention of CGD (Figure 2A,B–D right panels; Figure S8A–D; Tables S1H and S2C, Supporting Information) and on the inhibition of gallbladder MUC1 expression (Figure 3C,D) in WD‐fed mice^C70‐KO^. This occurred through the limitation of the PKCζ‐RBL1 pathway (Figure 5B) and the decrease of serum ceramide levels^[^ ^25^ ^]^ (Figure S7C and Table S5C, Supporting Information). In contrast, although oral TβMCA reduced the nuclear levels of PKCζ, PKCζ^pT410^, and RBL1^pT369^, it had little influence on the overall gallbladder PKCζ and PKCζ^pT410^ expression compared to ACPD and GlyMCA treatment (Figure 5B). This suggests that the protective effects of TβMCA against CGD development may involve different signaling pathways compared to ACPD and GlyMCA.

### 2.4 Loss of SDPR Initiated Nuclear Translocation of PKCζ

Our work noted that WD‐fed mice^WT^ and mice^C70‐KO^, had similar NAFLD (Table S2A, Supporting Information) and serum ceramide (Table S5A, Supporting Information) characteristics. However, in contrast to the ^Hphil^BA pool characteristics of WD‐fed mice^WT^, the CGD model WD‐fed mice^C70‐KO^ and LD‐fed mice^WT^ showed nuclear accumulation of PKCζ in the gallbladder, which triggered the RBL1/Sp1 signaling to accelerate pro‐nucleating MUC1^[^ ^9^, ^11^ ^]^ transcription (Figure 5A). We also observed a similar nuclear PKCζ accumulation in gallbladder specimens from cholelithiasis patients (Figure S12A and Table S7, Supporting Information).

**Figure 6:**
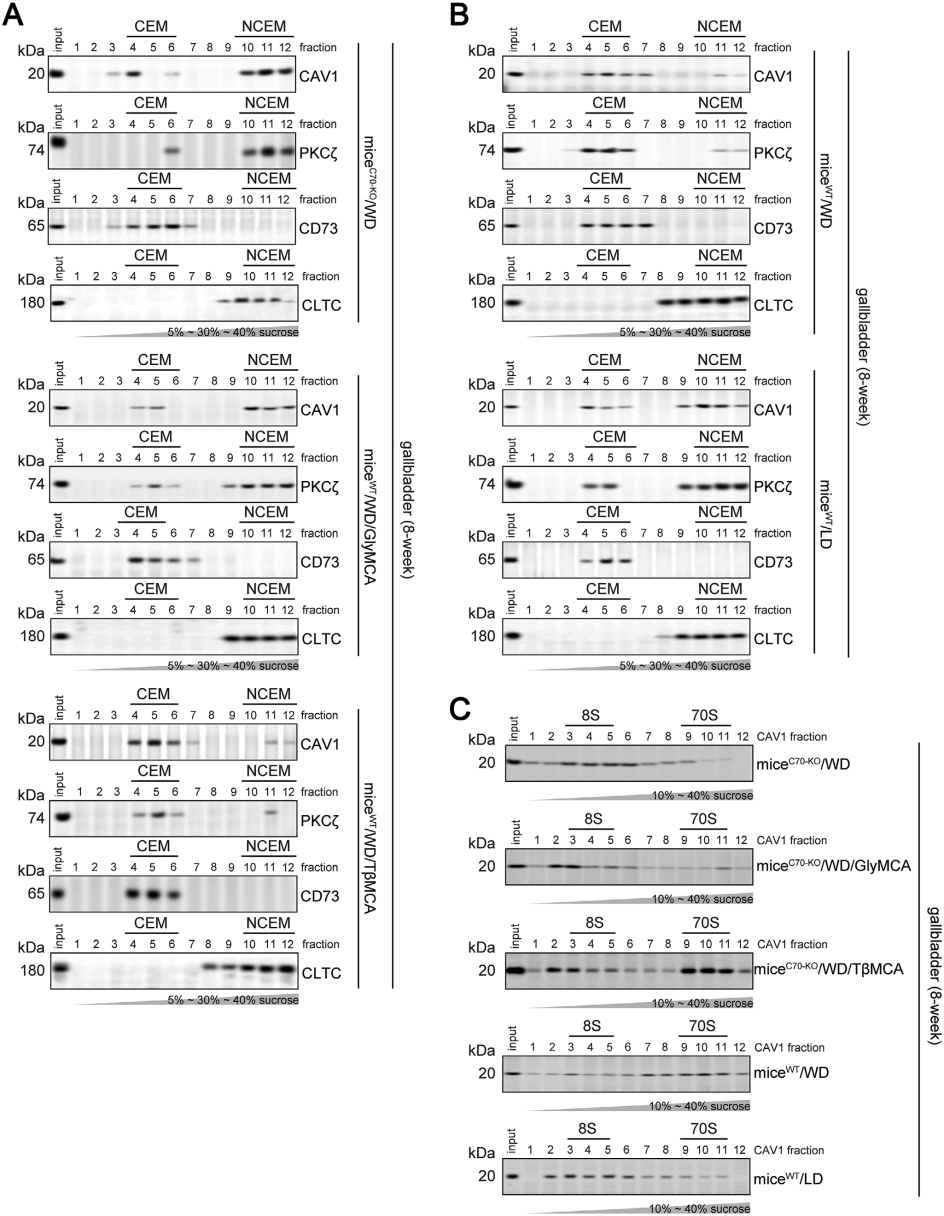
The reduced contents of PKCζ caveolae distribution and the instability of 70S‐CAV1 complexes in the gallbladder of WD‐fed mice^C70‐KO^. A,B) The sucrose linear (5%‐30%‐40%) gradients were performed to isolate CEM (fractions 4–6) or NCEM (fractions 10–12) fractions from the gallbladder lysates of mice^C70‐KO^/WD, mice^C70‐KO^/WD/GlyMCA, mice^C70‐KO^/WD/TβMCA (A) and mice^WT^/WD, mice^WT^/LD (B). Note that CAV1 and PKCζ are mainly localized in the CEM of the gallbladder of mice^C70‐KO^/WD/TβMCA and mice^WT^/WD. CD73 was used as CEM subcellular markers, and CLTC (clarthin heavy chain) was used as NCEM subcellular markers. C) The sucrose linear (10%‐40%) gradients were performed to isolate 8S‐CAV1 (fractions 3 to 5) and 70S‐CAV1 (fractions 9 to 11) complexes in the gallbladder of mice^C70‐KO^/WD, mice^C70‐KO^/WD/GlyMCA, mice^C70‐KO^/WD/TβMCA, mice^WT^/WD and mice^WT^/LD. WD, fed on western‐style diet; LD, fed on lithogenic diet. Each experimental group comprised 10 mice. mice^WT^/WD, WD‐fed mice^WT^; mice^WT^/LD, LD‐fed mice^WT^; mice^C70‐KO^/WD, WD‐fed mice^C70‐KO^; mice^C70‐KO^/WD/GlyMCA, GlyMCA‐treated (50 mg kg^−1^ per day) WD‐fed mice^C70‐KO^; mice^C70‐KO^/WD/TβMCA, TβMCA‐treated (500 mg kg^−1^ per day) WD‐fed mice^C70‐KO^; mice^C70‐KO^/WD/ACPD, ACPD‐treated (10 mg kg^−1^ per day) WD‐fed mice^C70‐KO^.

Previous studies have reported that PKCζ localizes in the clustered caveolae membrane, acts downstream of ceramide, and contributes to the progression of NAFLD.^[^ ^17^, ^26^ ^]^ However, we found that PKCζ was slightly present in the caveolae‐enriched membrane (CEM) and mainly occurred in the non‐CEM (NCEM) domains of the plasma membrane of the gallbladder in WD‐fed mice^C70‐KO^ and LD‐fed mice^WT^ (**Figure** **6**A,B). In contrast, in WD‐fed mice^WT^, PKCζ was more localized in the CEM domains of the plasma membrane of the gallbladder (Figure 6B). Caveolin‐1 (CAV1) is the primary constituent of CEM.^[^ ^27^ ^]^ We previously found that CAV1 deficiency sensitizes mice to CGD.^[^ ^3^, ^13^ ^]^ We detected no reductions in CAV1 mRNA levels in the gallbladder of mice^WT^ and mice^C70‐KO^ after they were fed the WD (Figure S13A, Supporting Information), whereas mice^C70‐KO^ had significantly lower levels of CAV1 protein in the gallbladder in comparison to mice^WT^ (Figure 5A). Normally, CAV1 protein is presented in two distinct oligomeric species sedimenting at 8S and 70S, and the CEM is enriched in 70S complexes of CAV1 (70S‐CAV1).^[^ ^28^ ^]^ In comparison with WD‐fed mice^WT^, LD‐fed mice^WT^ and WD‐fed mice^C70‐KO^ showed an increased amount of unassembled 8S‐CAV1 oligomers in the gallbladder (Figure 6C). The retention of PKCζ within NCEM and the accumulation of 8S‐CAV1 complexes were also observed in the gallbladder of cholelithiasis patients (Figure S12B,C, Supporting Information). These observations led us to explore whether the change in the hydrophobic bile acid pool altered the CEM structure (the formation of 70S complexes of CAV1) of the gallbladder, thereby causing the nuclear accumulation of PKCζ protein in the gallbladder of the CGD model mice and gallstone patients.

The formation of 70S complexes of CAV1 relies on the interaction among CAV1, polymerase I‐transcript release factor (PTRF) and serum deprivation‐response gene (SDPR).^[^ ^27^, ^29^ ^]^ These proteins are also co‐regulated with regard to their protein stability and are interdependent in the process.^[^ ^29b,c^ ^]^ No changes in PTRF mRNA expression were detected in the gallbladders of mice^C70‐KO^ when compared with mice^WT^ after being fed a WD, while the SDPR gene expression was reduced (Figure S13A, Supporting Information). It is evident that only oral TβMCA treatment was effective in restoring the expression of caveolae constituents, including the increase of SDPR mRNA levels (Figure S13B, Supporting Information), expression of CAV1, PTRF, and SDPR proteins (Figure 5B), PKCζ caveolar distribution, and stability of 70S‐CAV1 complexes in the gallbladder of mice^C70‐KO^ (Figure 6A,C). In contrast, neither ACPD treatment (Figure 5B; Figure S13B, Supporting Information) nor oral GlyMCA was able to achieve this effect (Figures 5B and 6A; Figure S13B, Supporting Information). However, in GBECs treated with a mixture of ^Hpho^BA and ceramide, both TβMCA and GlyMCA treatments were capable of rescuing the expression of caveolae constituents (CAV1, PTRF, and SDPR) and enabling PKCζ to migrate to caveolae (Figures S11B and S15A,B, Supporting Information). This phenomenon explained the undetectable levels of GlyMCA in the gallbladder after oral administration^[^ ^7b^ ^]^ (Figure S16A,B, Supporting Information), which also evidenced the role of FXR on SDPR genedown‐regulation, nuclear PKCζ translocation, and the redistribution of assembled 70S‐CAV1 into unassembled 8S‐CAV1.

**Figure 7:**
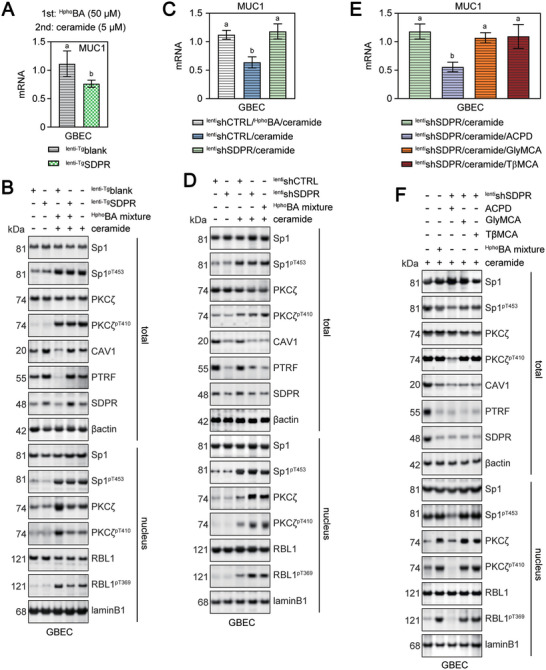
The change of SDPR expression on the nuclear PKCζ localization and MUC1 gene transcription. A,B) GBEC were stably expressing lentivirus carrying blank vector (^lenti‐Tg^Blank) or ^lenti‐Tg^SDPR. Cells were divided into 2 groups and treated with 50 µm ^Hpho^BA mixture for 24 h, and then were stimulated with 5 µm ceramide for 2 h. Levels of MUC1 mRNA in each group of GBEC were detected by qRT‐PCR. Expression data were normalized to the expression of 18s RNA (A). WB analysis of Sp1 and its phosphorylated form pT453, PKCζ and its phosphorylated form pT410, RBL1 and its phosphorylated form pT369, CAV1, PTRF, and SDPR expression in each group of GBEC. The expression of βactin or laminA was used as a loading internal control for total or nuclear extracts, respectively. Unstimulated cells were used as control (B). C,D) GBEC were stably expressing lentivirus carrying shCTRL (^lenti^shCTRL) or ^lenti^shSDPR. Cells were divided into 3 groups and treated without or with 50 µm ^Hpho^BA mixture for 24 h, and then were stimulated with 5 µm ceramide for 2 h. Levels of MUC1 mRNA in each group of GBEC were detected by qRT‐PCR. Expression data were normalized to the expression of 18s RNA (C). WB analysis of Sp1 and its phosphorylated form pT453, PKCζ and its phosphorylated form pT410, RBL1 and its phosphorylated form pT369, CAV1, PTRF, and SDPR expression in GBEC. The expression of βactin or laminA was used as a loading internal control for total or nuclear extracts, respectively. Unstimulated cells were used as control (D). ^lenti^shCTRL/^Hpho^BA/ceramide, GBEC stably expressing lentivirus carrying shCTRL were treated with 50 µm ^Hpho^BA mixture for 24 h, and then were stimulated with 5 µm ceramide for 2 h. ^lenti^shCTRL/ceramide, GBEC stably expressing lentivirus carrying shCTRL were stimulated with 5 µM ceramide for 2 h. ^lenti^shSDPR/ceramide, GBEC stably expressing lentivirus carrying shSDPR were stimulated with 5 µm ceramide for 2 h. The compositions of ^Hpho^BA mixture used in these in vitro studies were shown in Table S4 (Supporting Information). E,F) GBEC were stably expressing lentivirus carrying ^lenti^shSDPR. Cells were divided into 4 groups and stimulated with 5 µm ceramide for 2 h. Some of the cells were left untreated after ceramide stimulation, while others were treated with ACPD (5 µm), GlyMCA (50 µm), or TβMCA (200 µm), respectively. Levels of MUC1 mRNA in each group of GBEC were detected by qRT‐PCR. Expression data were normalized to the expression of 18s RNA (E). WB analysis of Sp1 and its phosphorylated form pT453, PKCζ and its phosphorylated form pT410, RBL1 and its phosphorylated form pT369, CAV1, PTRF, and SDPR expression in GBEC. The expression of βactin or laminA was used as a loading internal control for total or nuclear extracts, respectively. The cells stimulated only with ceramide (5 µm, 2 h) were used as control (F). ^lenti^shSDPR/ceramide, GBEC stably expressing lentivirus carrying shSDPR were stimulated with 5 µm ceramide for 2 h. ^lenti^shSDPR/ceramide/ACPD, GBEC stably expressing lentivirus carrying shSDPR were stimulated with ACPD (5 µm) and ceramide (5 µm) for 2 h. ^lenti^shSDPR/ceramide/GlyMCA, GBEC stably expressing lentivirus carrying shCTRL were treated with 50 µM GlyMCA for 24 h, and then were stimulated with 5 µm ceramide for 2 h. ^lenti^shSDPR/ceramide/TβMCA, GBEC stably expressing lentivirus carrying shCTRL were treated with 200 µm TβMCA for 24 h, and then were stimulated with 5 µm ceramide for 2 h. GBEC, gallbladder epithelial cells (HIBEpiC). Data with different lowercase letter indicates significant differences (*P <* 0.05) between each assigned GBEC group. The data for each cell experiment group is derived from 5 independent experiments. The data is presented as mean ± standard deviation. Data normality was determined by using Shapiro‐Wilkes test. Differences (*p* < 0.05 considered significant) between two independent samples with normal distribution were determined by using Student's t‐test, while Mann‐Whitney test was used to compare non‐normal data (*p* < 0.05 considered significant). For more than two normally distributed samples, statistical comparisons were made by ANOVA with Tukey's range test. And Kruskal‐Wallis non‐parametric ANOVA with Benjamini‐Hochberg corrections was used to compare more than two samples with non‐normal distribution. An adjusted *p* < 0.05 was considered significant after Tukey's or Benjamini‐Hochberg corrections for multiple comparisons. Individual *p* values were provided in Primary Data for Figure 7.

SDPR overexpression in GBECs via lentiviral transduction (^lenti‐Tg^SDPR) restored CAV1 and PTRF protein expression, reduced nuclear PKCζ and RBL1 activities, and alleviated MUC1 mRNA expression when treated with a mixture of ^Hpho^BA and ceramide (**Figure** **7**A,B). However, SDPR knockout (^lenti^shSDPR) in GBECs reduced CAV1 and PTRF protein expression, promoted nuclear PKCζ and RBL1 activities, and promoted MUC1 mRNA expression when treated with ceramide alone (Figure 7C,D). Moreover, MUC1 mRNA expression normalized after treating ceramide‐stimulated SDPR‐knockout GBECs with the PKCζ inhibitor ACPD, but not the FXR antagonists GlyMCA or TβMCA. This occurred through the inhibition of cytoplasmic and nuclear PKCζ activation (Figure 7E,F).

Interestingly, the increased nuclear PKCζ^pT410^ levels and the reduced CAV1 protein expression after WD consumption were discovered in the livers of both mice^WT^ and mice^C70‐KO^ (Figure 5C). PKCζ protein was mostly found in the NCEM, and CAV1 mainly presented a noncaveolar surface pool composed of 8S complexes in the livers of mice^WT^ and mice^C70‐KO^ (Figure S14A,B, Supporting Information). This might be the result of hepatocytes lacking expression of PTRF and SDPR, as reported previously^[^ ^27^, ^29^ ^]^ (Figure 5C).

### 2.5 Mir30c/e Regulates Gallbladder SDPR Gene Following Activation of FXR

The SDPR mRNA levels and 8S‐CAV1 protein fractions were similarly decreased and increased in the gallbladder of mice^WT^ after 7 days of feeding 0.5% CA as compared with WD‐fed mice^C70‐KO^, but this was not observed in FXR‐knockout (FXR‐KO) mice^FXR‐KO^ (Figure S13C,D, Supporting Information). Given the reported role of FXR‐miRNA interaction in various conditions,^[^ ^30^ ^]^ we tested whether SDPR mRNA reduction is mediated by a specific miRNA in CGD mice and whether miRNA‐based therapy could modulate gallbladder SDPR expression. Bioinformatics analyses were done using Targetscan and a cutoff of at least 10% for the probability of preferentially conserved targeting score.^[^ ^31^ ^]^ Our analyses revealed 9 putative miRNA target sites for the 3′‐untranslated region (3′‐UTR) of SDPR mRNA among mammalian species (Figure S17A and Table S8, Supporting Information).

Of these 9 miRNAs, miR‐30c, and miR‐30e (miR30c/e) were significantly induced in the gallbladders of mice^C70‐KO^ (Figure S17B, Supporting Information). Feeding with 0.5% CA failed to induce the upregulation of gallbladder miR30c/e expression in mice^FXR‐KO^ (Figure S17C, Supporting Information). We also used a pGL3 luciferase reporter plasmid containing the complete 3′‐UTR of the human *SDPR* gene and found that miR30 over‐expression (^lenti‐Tg^miR30) could reduce SDPR gene transcription (Figure S17D,E, Supporting Information).

miR30c/e is located within intron 5 of the nuclear transcription factor Y subunit γ (*NFYC*) gene. The conserved FXR‐binding motif was detected within intron 1 of the *NFYC* gene by liver chromatin immunoprecipitation (ChIP)‐seq analysis in FXR‐activated mice,^[^ ^32^ ^]^ and we obtained this result both in vitro and in vivo(Figure S18A–C, Supporting Information). TβMCA treatment reduced miR30c/e in the gallbladder of mice^C70‐KO^, whereas oral GlyMCA and ACPD injection showed no effect on it (Figure S17F, Supporting Information).

**Figure 8:**
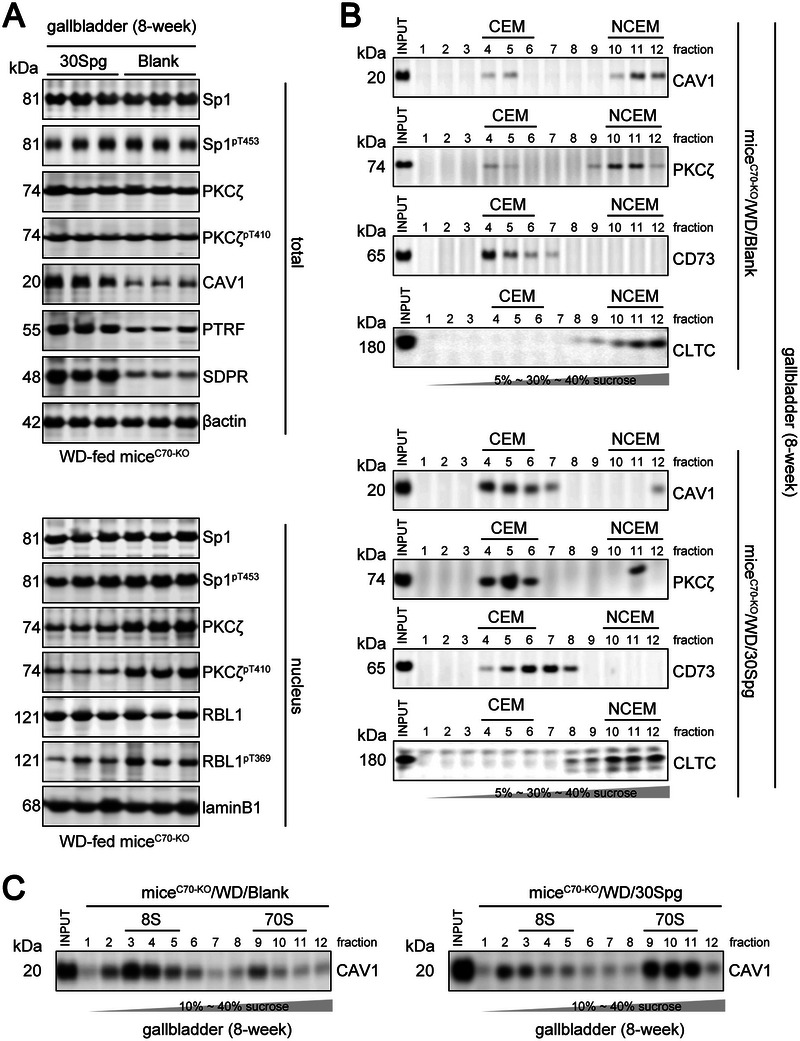
A reduced nuclear PKCζ translocation and an improved CEM distribution of PKCζ and 70S scaffolds of CAV1 oligomers in the gallbladder of WD‐fed mice^C70‐KO^ with miR‐30 Sponge over‐expression. A, WB analysis of Sp1 and its phosphorylated form pT453, PKCζ and its phosphorylated form pT410, RBL1 and its phosphorylated form pT369, CAV1, PTRF, and SDPR expression in gallbladder lysates collected from mice^C70‐KO^/WD/Blank and mice^C70‐KO^/WD/30Spg. The expression of βactin or laminB1 was used as a loading internal control for total or nuclear extracts, respectively. B) The sucrose linear (5%‐30%‐40%) gradients were performed to isolate CEM (fractions 4–6) or NCEM (fractions 10–12) fractions from the gallbladder of mice^C70‐KO^/WD/Blank and mice^C70‐KO^/WD/30Spg. Note that CAV1 and PKCζ are mainly localized in the CEM of the gallbladder of mice^C70‐KO^/WD/30Spg. CD73 was used as CEM subcellular markers, and CLTC (clarthin heavy chain) was used as NCEM subcellular markers. C, The sucrose linear (10%‐40%) gradients were performed to isolate 8S‐CAV1 (fractions 3 to 5) and 70S‐CAV1 (fractions 9 to 11) complexes in the gallbladder of mice^C70‐KO^/WD/Blank and mice^C70‐KO^/WD/30Spg. Note that the increased amounts of 8S complexes present in the gallbladder of mice^C70‐KO^/WD/Blank. Each experimental group comprised 10 mice. WD, fed on western‐style diet. mice^C70‐KO^/WD/Blank, AAV‐Null‐virus injected WD‐fed mice^C70‐KO^; mice^C70‐KO^/WD/30Spg, AAV‐30Spg‐virus injected WD‐fed mice^C70‐KO^.

**Figure 9:**
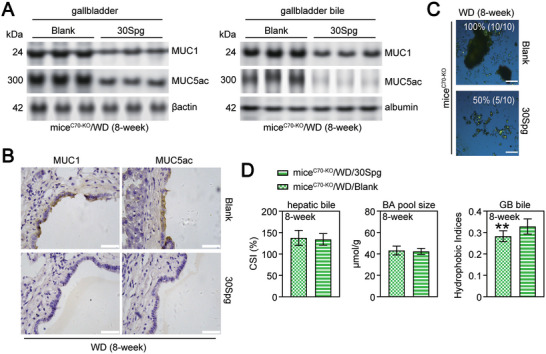
The inhibition of MIR30 on the prevention of CGD. A) WB analysis of MUC1 and MUC5ac expression in gallbladder tissues (left) and gallbladder bile (middle) collected from mice^C70‐KO^ after 8‐week WD feeding, with the i.p. injection of Blank or 30Spg. The expression of βactin or albumin was used as a loading internal control for cytoplasmic or bile extracts, respectively. B) Immunohistochemical staining of MUC1 and MUC5ac expression in gallbladder tissues (right) collected from mice^C70‐KO^ after 8‐week WD feeding, with the intraperitoneal injection of Blank or 30Spg (scale bar: 100 µm). C) the polarizing light microscopy examination of cholesterol crystals (scale bar: 100 µm). D, CSI values, BA pool size and the hydrophobic indices of the gallbladder bile. GB, gallbladder; BA, bile acid. WD, fed on western‐style diet. mice^C70‐KO^/WD/Blank, AAV‐Null‐virus injected WD‐fed mice^C70‐KO^; mice^C70‐KO^/WD/30Spg, AAV‐30Spg‐virus injected WD‐fed mice^C70‐KO^. **p < 0.01; significant difference between mice^C70‐KO^/WD/Blank versus mice^C70‐KO^/WD/30Spg. Each experimental group comprised 10 mice. The data is presented as mean ± standard deviation. Data normality was determined by using Shapiro‐Wilkes test. Differences (*p* < 0.05 considered significant) between two independent samples with normal distribution were determined by using Student's t‐test, while Mann‐Whitney test was used to compare non‐normal data (*p* < 0.05 considered significant). Individual *p* values were provided in Primary Data for Figure 9.

**Figure 10:**
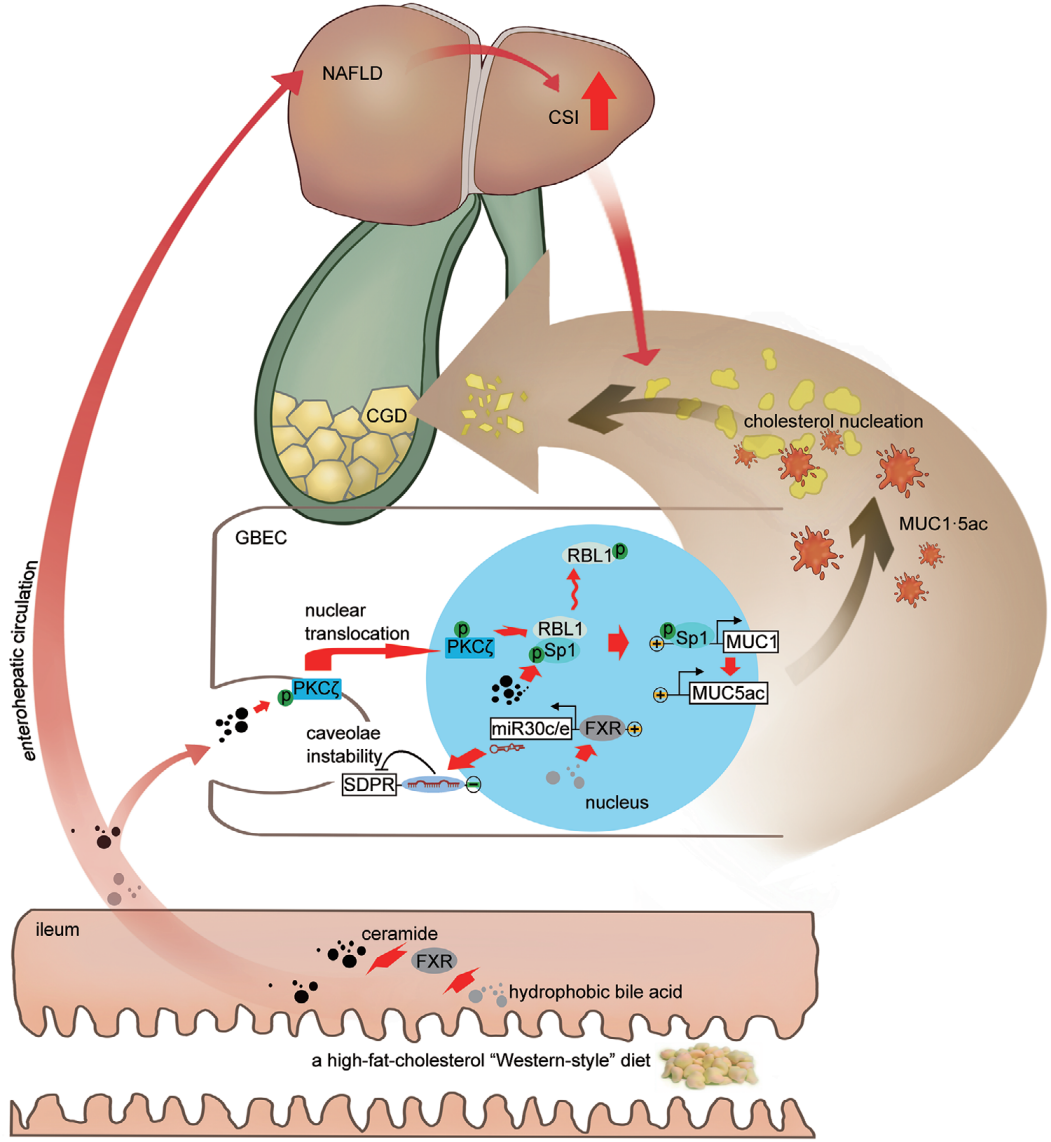
A possible pathological mechanism linking gallbladder FXR Activation to cholesterol gallstone formation.

Finally, we found that miR30c/e deletion (AAV‐mediated miR‐30 Sponge over‐expression, 30Spg^[^ ^33^ ^]^; Figure S4E, Supporting Information) did not affect the NAFLD characteristics of WD‐fed mice^C70‐KO^ (Tables S2D and S5D, Supporting Information). However, it significantly increased the expression of CAV1, PTRF, and SDPR and restored the CEM distribution of PKCζ and 70S scaffolds of CAV1 oligomers (**Figure** **8**A–C). Furthermore, it decreased gallbladder MUC1·5ac expression and played a role in CGD prevention that was independent of biliary CSI (**Figure** **9**A–D; and Table S1J, Supporting Information). This occurred via the reduction of nuclear PKCζ‐RBL1 responses in the gallbladder (Figure 8A). These findings provide compelling evidence for miR30c/e being a direct transcriptional target of FXR. The activation of the FXR‐miR30c/e‐SDPR axis in the gallbladder of mice^C70‐KO^ initiates caveolar instability and nuclear PKCζ‐RBL1‐Sp1‐MUC1 transcription, which promotes the formation of cholesterol gallstones (**Figure** **10**).

Under hydrophobic bile acid pools, a high‐fat‐cholesterol “Western‐style” diet induces NAFLD and elevated bile CSI, simultaneously activating intestinal FXR to increase circulating ceramide. Subsequently, hydrophobic bile acids stimulate gallbladder FXR‐miR30c/e activation, which induces SDPR gene suppression and caveolae instability. This pathway, in conjunction with ceramide‐mediated activation of gallbladder PKC**ζ**, promotes PKCζ translocation from its original location in gallbladder epithelial membrane caveolae to the nucleus, where it activates the RBL1‐Sp1 axis. The activated axis then induces gallbladder hypersecretion of MUC1·5ac, which, combined with elevated bile CSI, ultimately facilitates cholesterol gallstone formation.

## 3 Conclusion

NAFLD and CGD share many features of dysregulated metabolic stress, such as elevated circulating ceramide levels, increased lipid accumulation, disrupted bile acid metabolism, and chronic inflammation.^[^ ^2^, ^7^, ^34^ ^]^ NAFLD mouse models are generally reliable for simulating the disease progression observed in humans. However, while traditional LD‐fed CGD mouse models exhibit an elevated bile CSI, they also show an increased bile acid pool size, which contrasts with the reduced bile acid pool found in human cases.

In this study, we identified the mice^C70‐KO^, which more closely mimics the human condition by replicating the hydrophobic BA (^Hpho^BA) pool.^[^ ^10^ ^]^ When fed a high‐fat‐cholesterol “Western‐style” diet (WD), these mice exhibited CGD features, including a reduced bile acid pool size similar to that of human patients.^[^ ^35^ ^]^ This makes the mice^C70‐KO^ a particularly valuable model for studying CGD and developing potential therapeutic interventions.

A central finding of our research is the critical role of gallbladder mucus, specifically MUC1 and MUC5ac, in regulating CGD development independently of bile CSI.^[^ ^9^, ^11^ ^]^ While bile CSI is typically seen as a key driver of gallbladder cholesterol crystallization, our results suggest that changes in MUC1 and MUC5ac expression are equally crucial in promoting gallstone formation. We observed that the overproduction of gallbladder mucus is driven by gallbladder‐specific ^Hpho^BA‐FXR activation in mice^C70‐KO^, a mechanism not present in mice^WT^ with hydrophilic BA pool. This difference may explain why both WD‐fed mice^WT^ and mice^C70‐KO^ displayed highly supersaturated bile (CSI > 100)^[^ ^9^ ^]^ and gallbladder hypomotility, yet CGD rarely developed in WD‐fed mice^WT^. Moreover, as demonstrated in previous studies and corroborated by our data, MUC1 regulates MUC5ac expression,^[^ ^9^, ^11^, ^13^ ^]^ further amplifying lithogenesis. This discovery shifted our focus to investigating the mechanisms controlling MUC1 expression, which we identified as essential for understanding CGD pathogenesis. Our in vitro work using GBEC also demonstrated that MUC1 expression is regulated by ^Hpho^BA through the FXR signaling pathway. These findings emphasize the importance of the gallbladder ^Hpho^BA‐FXR‐MUC1 signaling axis in driving CGD.

We further examined the effects of GlyMCA and TβMCA, two hydrophilic BA and FXR antagonists,^[^ ^8^, ^19^ ^]^ on this pathway. While both compounds effectively downregulated MUC1 expression in vitro, they worked through distinct mechanisms in vivo to prevent CGD. GlyMCA, which cannot undergo enterohepatic circulation to reach the gallbladder,^[^ ^7^, ^8^ ^]^ prevents CGD by inhibiting WD‐induced intestinal FXR‐ceramide signaling,^[^ ^8^ ^]^ reducing systemic metabolic stress and alleviating bile CSI. In contrast, TβMCA acts directly on the gallbladder by functioning as an FXR antagonist.^[^ ^7^ ^]^ Although TβMCA is hydrolyzed into βMCA by intestinal BSH^[^ ^7^ ^]^ during intestinal absorption, our study shows that βMCA is re‐converted to TβMCA in the liver, even under WD feeding conditions. This re‐converted TβMCA is then delivered to the gallbladder, where it inhibits FXR, reducing MUC1 transcription and preventing CGD. This distinction highlights different therapeutic strategies for CGD based on whether FXR signaling is targeted in the intestine or gallbladder.

In addition, we identified the PKCζ‐RBL1‐Sp1 signaling pathway as a key regulator of MUC1 transcription, offering new insights into how MUC1 drives CGD. It is well‐established that Sp1 acts as a transcription factor for MUC1 and is also a critical regulator of NAFLD progression.^[^ ^20^ ^]^ This explains why no significant differences were observed in Sp1 expression or activation between WD‐fed mice^WT^ and mice^C70‐KO^. However, our work focused on PKCζ and RBL1, two proteins that modulate Sp1 activity in opposing ways.^[^ ^23^ ^]^ We showed that PKCζ phosphorylates RBL1 at T369, a phosphorylation event that plays a pivotal role in enhancing Sp1‐mediated transcription. While earlier studies have demonstrated that RBL1‐T369 phosphorylation releases E2F transcription factors,^[^ ^24^ ^]^ we found that phosphorylation at this site also releases Sp1, allowing it to drive MUC1 transcription during gallstone formation. This builds upon previous work,^[^ ^20^, ^23^, ^24^ ^]^ revealing a novel regulatory mechanism in which PKCζ‐driven RBL1 phosphorylation fully activates Sp1, contributing to MUC1 upregulation and CGD development.

Furthermore, we demonstrated the role of ACPD, a specific PKCζ inhibitor,^[^ ^25^ ^]^ in this pathway. By inhibiting PKCζ, ACPD blocks RBL1 phosphorylation at T369, preventing Sp1 activation and subsequently downregulating MUC1 expression. In vivo, ACPD's preventive effect on CGD mirrors that of GlyMCA, as both target ceramide synthesis and signaling, which are critical factors in both NAFLD and CGD progression.^[^ ^7^, ^8^ ^]^

Our study also uncovered that increased nuclear accumulation of PKCζ occurred only in the gallbladders of mice^C70‐KO^ and did not rely on WD feeding. Notably, this phenomenon was similarly observed in the gallbladders of cholelithiasis patients, suggesting a ^Hpho^BA‐driven mechanism independent of dietary factors. Previous reports in other tissues had indicated that elevated circulating ceramide induces PKCζ localization to caveolae membrane domains,^[^ ^26a^ ^]^ yet our findings showed that PKCζ predominantly localized in non‐caveolae domains in the gallbladders of mice^C70‐KO^, a pattern also observed in human gallbladders. This discrepancy prompted us to investigate further, leading to the identification of a novel FXR‐miR30c/e‐SDPR signaling axis that regulates caveolae structure and PKCζ nuclear localization in the gallbladder of mice^C70‐KO^, linking FXR activation to MUC1 overexpression.

Previously, we demonstrated that CAV1 deficiency accelerates CGD,^[^ ^3^, ^13^ ^]^ and other studies have shown that CAV1 deficiency promotes PKCζ‐mediated Sp1 activation.^[^ ^36^ ^]^ Given that caveolae structures are composed of CAV1, PTRF, and SDPR, which mutually regulate each other's stability,^[^ ^27^, ^29^ ^]^ we found that downregulation of SDPR mRNA by FXR‐miR30c/e pathway in mice^C70‐KO^ led to a corresponding reduction in CAV1 and PTRF protein expression, resulting in caveolae instability. This instability prevented the localization of PKCζ to the membrane and instead facilitated its nuclear translocation, where it activated the Sp1‐MUC1 transcriptional pathway, promoting gallstone formation. Our manipulation of SDPR expression in vitro confirmed that changes in SDPR directly affect caveolae stability and PKCζ nuclear localization, providing strong evidence for the critical role of this pathway in CGD pathogenesis. And in vivo inhibition of miR30c/e significantly reduced MUC1 expression and CGD incidence in WD‐fed mice^C70‐KO^, resembling the action of TβMCA in restoring SDPR expression and maintaining caveolae integrity.

In conclusion, our study provides novel insights into the pathogenesis of CGD. Notably, we identified the mice^C70‐KO^ as an effective model for simulating human CGD, and highlighted the critical role of gallbladder mucus in gallstone formation independent of CSI. Additionally, we uncovered the gallbladder FXR‐miR30c/e‐SDPR axis as a novel mechanism linking caveolae disruption, nuclear PKCζ‐RBL1‐Sp1 signaling and MUC1‐regulated gallstone formation. These findings expand our understanding of CGD pathogenesis and open new avenues for the development of targeted therapeutic strategies.

## 4 Experimental Section

### Animals, Diet and Drug treatment

All animal experiments (*n* = 10 for each background, mean age: 3‐month‐old male mice) were approved by the Animal Experimental Ethical Inspection of the First Affiliated Hospital, College of Medicine, Zhejiang University (2020‐1288). At the desired experimental endpoint, mice were euthanized for ex vivo analysis after 6 h fasting. The clustered regularly interspaced short palindromic repeats (CRISPR)/CRISPR‐associated protein 9 (Cas9) system^[^ ^37^ ^]^ was used to establish mice^C70‐KO^ on a C57BL/6 genetic background, which are described in Figure S19A–C and Table S9A (Supporting Information). The construction of mice^C70‐KO^ with AAV‐mediated Cas9‐based MUC1 gene deficiency (sgMUC1), mice^C70‐KO^ with AAV‐mediated Cas9‐based MUC4 gene deficiency (sgMUC4), mice^C70‐KO^ with AAV‐mediated Cas9‐based MUC5ac gene deficiency (sgMUC5ac), mice^WT^ with adeno‐associated‐viral mediated (AAV‐mediated) MUC1 overexpression (MUC1‐OE), and mice^C70‐KO^ with AAV‐mediated miR‐30 Sponge^[^ ^33^ ^]^ (30Spg) also are described in Figures S4A–E and S19A and Tables S9B–D (Supporting Information). mice^FXR‐KO^ were purchased from the Jackson Laboratory. Control groups also included wild‐type C57BL/6 mice (mice^WT^), as well as mice^C70‐KO^ administered AAV expressing either Cas9 with a non‐targeting sgRNA (sgCTRL) or no transgene (Blank; AAV‐Null).

All mice were housed in the pathogen‐free animal facility under controlled conditions of humidity (55 ± 5%), lighting (12 h light/dark cycle) and temperature (23 °C), and were given diet (Table S10, Supporting Information) and water ad libitum. The drinking water contained vitamin supplements (Table S3, Supporting Information), which could obviously alleviate the increased serum levels of liver damage markers ALT and AST and restore the expression of genes encoding classical BA synthetic enzyme (Cyp7a1/8b1) in mice^C70‐KO^ (Figure 1A–F, Figure S20A–D and Table S11A, Supporting Information) as previously described.^[^ ^38^ ^]^

For oral GlyMCA (dose of 50 mg kg^−1^ per day) or oral TβMCA (dose of 500 mg kg^−1^ per day) administration (Figures S21A,B and Table S11B, Supporting Information), the ingredients of bacon‐flavored WD were thoroughly mixed with GlyMCA or TβMCA during diet preparation. For drug treatment, daily subcutaneous injection of ACPD (10 mg kg^−1^) was given 1 day prior to the beginning of WD feeding.

### Chemical Reagents, Antibodies, and Cell Lines

Chemical reagents, antibodies, and cell lines used are described in Table S4 (Supporting Information). Here the cultured human intrahepatic biliary epithelial cells (HIBEpiC) was used as GBEC model for studying the regulation of MUC1 and MUC5ac (MUC1·5ac) expression in vitro.

### Human Gallbladder Sample Studies

The use of human gallbladder samples for this study was approved by the Clinical Research Ethics Committee of the First Affiliated Hospital, College of Medicine, Zhejiang University (2020‐936). Gallbladder samples (*n* = 7) were obtained with informed consent from patients (Table S7, Supporting Information) undergoing laparoscopic cholecystectomy. Prior to surgery, patients agreed to the use of their cholecystectomy specimens for non‐profit scientific research purposes.

### Myograph Studies on the Contractility of Gallbladder

The contractility of gallbladders from the experimental mice were measured using a MyoMED myograph system (MED Associates, Fairfax, VT). After anesthetized euthanasia, the gallbladders were removed and the gallbladder strips (measuring from 3 to 10 mm) were cut through the whole wall. The silk threads were attached to each end of the strips. Each strip was mounted in a tissue bath (15 mL volume) containing aerated (5% CO_2_/95% O_2_) physiological saline solution (PSS, containing 119 mm NaCl, 4.7 mmKCl, 24 mm NaHCO_3_, 1.2 mm KH_2_PO_4_, 2.5 mm CaCl_2_, 1.2 mm MgSO_4_, and 11 mm glucose; pH 7.4) at 37 °C. These gallbladder strips were placed under an initial resting tension equivalent to a 5 mN load and allowed to equilibrate for 60 min, with solution changes every 15 min. Agonist responses were obtained by applying the neurotransmitter cholecystokinin (10 nm; applied cumulatively; sulphated CCK‐8 was purchased from AS20741, AnaSpec, Fremont, CA) directly into the tissue bath. Responses were normalized to the wet weight of the tissue.

### Microscopic Examination and the NT of Cholesterol Crystals from Gallbladder Bile

Gallbladder bile were collected after mice were euthanized and were spread on glass slides. Isotropic gallbladder bile samples were first centrifuged to get the crystal‐free bile specimens, then were transferred into a sterile brown glass tube under nitrogen and placed in an incubator (without shaking during the observation period) at 37 °C. An aliquot was examined daily for the appearance of cholesterol monohydrate crystals under the Leica DM5000 polarizing microscope. NT was recorded as the earliest appearance of cholesterol monohydrate crystals. Sample observation was terminated at 21‐day even if no crystals had appeared. When cholesterol crystals did not appear during the observation period, the NT was described as 21‐day.

### Histological Evaluation, Hepatobiliary&Serum Chemistry, and Biliary CSI Estimation

The liver and gallbladder tissues from experimental mice were fixed in 4% formaldehyde, embedded in paraffin, sectioned at 5 µm, and stained with H&E, PAS and/or Masson's trichrome staining. The phospholipid, cholesterol, triglyceride levels and total bile salts were measured using commercially available kits from DIAN Diagnostics Laboratory (Hangzhou, China). The hydrophobicity index of the bile alt pool was calculated according to Heuman.^[^ ^39^ ^]^ The biliary CSI was calculated according to Carey's critical tables.^[^ ^40^ ^]^ Quantification of ceramide and bile acid metabolites are described in Supporting Information.

### BSH Activity

First, fecal proteins were prepared from the fecal samples (0.5 g in 5 mL PBS) of experimental mice using ultrasound sonication in an ice bath. The lysates were then centrifuged at 4 °C, 13000 g for 30 min (Optima XPN‐100, BeckmanCoulter, Brea, CA) and the supernatants were transferred to new tubes. The fecal protein solutions were partially diluted with ten‐fold volumes of PBS to determine the protein concentration using a BCA Protein Assay Kit (23 225, Pierce, Rockford, IL). The original protein solutions were diluted to 2 mg mL^−1^ by PBS as the protein working solution. The BSH activities were predicted by a generation of d4‐CDCA from d4‐TCDCA (DLM‐9572‐PK, Cambridge Isotope Laboratories, Tewksbury, MA) by BSH proteins. The incubation was carried out in 200 uL of 3 mm sodium acetate buffer (pH5.2), which contained 0.1 mm d4‐TCDCA and 0.1 mg mL^−1^ fecal protein. The mixtures were incubated at 37 °C for 20 min and the reactions were stopped by plunging the samples into dry ice. Hundred microliters of methanol were added to the mixture, samples were vortexed for 5 min and then centrifuged at 4 °C, 13000 g for 30 min (Optima XPN‐100, BeckmanCoulter, Brea, CA). The supernatants were transferred to sampling vials for d4‐CDCA quantification by ultra‐performance liquid chromatography coupled with tandem quadrupole mass spectrometry (Waters, Milford, MA) to determine the BSH activity.

### qRT‐PCR Assays and PCA

The qRT‐PCR was performed using total RNA extracted from small intestine, liver, and gallbladder tissues of experimental mice or from in vitro GBEC model. All used primer sequences are listed in Table S4 (Supporting Information). Expression data were normalized to the expression of 18s rRNA.

PCA is an unsupervised analysis that reduces the dimension of the normalized gene expression data^[^ ^41^ ^]^ to show whether a grouping exists between mice^C70‐KO^ and mice^WT^ at WD feeding. The principal component 1 (PC1) mostly reflects the changes in the gene expression levels due to Cyp2c70 deletion and therefore separates mice^C70‐KO^ from mice^WT^, whereas PC2 mainly reflects the variations in the gene expression levels within the same mice group (within mice^WT^ or within mice^C70‐KO^ group). PCA was performed by using Origin (OriginPro Software, Northampton, MA).

### The Detection of Gallbladder MUC Proteins

Western blot was used to detect the presence of MUC1, MUC4, and MUC5ac proteins in the gallbladder tissue and bile from the experimental mice (The used antibodies are listed in Table S4, Supporting Information). The gallbladder bile from experimental mice were dialyzed at 4 °C against running water for 3 days and centrifuged (30000 g for 10 min; Optima XPN‐100, BeckmanCoulter, Brea, CA) afterward at 4 °C to remove the lipid compounds and the undissolved particles. Then, 250 µL of 7% trichloric acetic acid was added to 100 µL of the supernatant. This mixture was next centrifuged for 5 min at 15000 g and the supernatant was discarded. The precipitate, which contains the biliary proteins, was washed with 1 mL ethanol/diethyl ether (1:1, v/v). After further centrifugation at 5000 g for 5 min, the remaining protein residue was dissolved in 3 mL sodium borate buffer (0.2 m sodium borate, 0.5% sodium dodecyl sulphate, pH 8.5) and mixed with 100 µL of the fluorescamine solution (0.3 mg ml^−1^ fluorescamine in acetone; F9015, SigmaAldrich, St. Louis, MO) before being loaded onto the gel for western blot analysis.

### Measurement of Lipid Peroxidation

4‐HNE, an index of lipid peroxidation,^[^ ^16^ ^]^ was detected using a 4‐HNE Assay Kit (ab238538, Abcam, Bristol, UK).

### Luciferase Reporter Assay

Detailed procedures for luciferase reporter analysis are described in Supporting Information.

### Western Blot

Tissues collected from experimental mice, HIBEpiC lines and adult cholelithiasis patients were lysed with Thermo Radioimmunoprecipitation Assay Buffer (to obtain total protein) or Thermo NE‐PER™ nuclear and cytoplasmic extraction reagents (to obtain and nuclear proteins; ThermoFisher, Waltham, MA) and complete protease inhibitor (Roche, Basel, Switzerland) on ice. For western blot, the lysate proteins were resolved by sodium dodecyl‐sulfate polyacrylamide gel electrophoresis (SDS‐PAGE) and blotted onto nitrocellulose membranes to test the binding of the antibodies (The used antibodies are listed in Table S4, Supporting Information).

### Comparative Analysis of PKCζ Protein Distribution in Gallbladder from WD‐fed mice^WT^, LD‐fed mice^WT^, LD‐fed mice^WT^ and Cholelithiasis Patients

The objective of this experiment was to investigate the distribution of cytoplasmic/membrane fractions and nuclear fractions of the PKCζ protein in gallbladder tissues derived from WD‐fed mice^WT^ (*n* = 5), LD‐fed mice^WT^ (*n* = 5), LD‐fed mice^WT^ (*n* = 5) and cholelithiasis patients (*n* = 7). Gallbladder samples were lysed with Thermo Radioimmunoprecipitation Assay Buffer (to obtain total protein) or Thermo Subcellular Protein Fractionation Kit (to separately obtain cytoplasmic/membrane and nuclear proteins; ThermoFisher, Waltham, MA) and complete protease inhibitor (Roche, Basel, Switzerland) on ice, and the protein extracts were separated into cytoplasmic/membrane, nuclear, and total fractions (the total fractions represent the sum of the cytoplasmic/membrane and nuclear fractions). For western blot, the proteins from these three fractions were resolved by SDS‐PAGE and blotted onto nitrocellulose membranes to test the binding of the PKCζ proteins (The used antibodies are listed in Table S4, Supporting Information). ImageJ software (version 1.50i, NIH, Bethesda, MA) would be used to quantify the intensity of the PKCζ protein bands in the cytoplasmic/membrane, nuclear, and total fractions. The intensities of the PKCζ bands from the cytoplasmic/membrane or nuclear fractions would be normalized to the band intensities from the total fractions, which served as an internal control. The ratio of cytoplasmic/membrane to nuclear fractions of PKCζ would be calculated and compared among WD‐fed miceWT, LD‐fed miceWT, LD‐fed miceWT and cholelithiasis patients groups. Statistical analysis would be conducted using Prism (GraphPad Software, San Diego, CA), applying one‐way analysis of variance (ANOVA) with Tukey's range test to compare the differences in PKCζ protein distribution among the four groups.

### Isolation of Caveolae‐Enriched Membranes

Liver and gallbladder tissues and HIBEpiC lines were lysed with 0.5% TritonX‐100 (X100, SigmaAldrich, St. Louis, MO) and complete protease inhibitor (11 697 498 001, Roche, Basel, Switzerland) on ice. The resulting lysates were mixed with the same volume of 80% (w/v) sucrose, 25 mm 2‐(N‐morpholino) ethanesulfonic acid (pH 6.0) and 150 mm NaCl and loaded at the bottom of a discontinuous 5%/30%/40% sucrose gradient. The samples were ultracentrifuged at 200000 × g (Optima TLX, BeckmanCoulter, Brea, CA) for 20 h at 4 °C. 12 fractions (300 µL) of samples were collected from the top to the bottom and mixed 1:1 with cold acetone for 4 h at ‐20 °C. The precipitated proteins were centrifuged at 16000 × g (Optima XPN‐100, BeckmanCoulter, Brea, CA) for 10 min and dried until required. Then a 12‐well format was used for the western blot analysis of the sucrose gradient fractions. And as previously described,^[^ ^42^ ^]^ the CEM was enriched with marker proteins like CAV1 and was expected to be detected in the lower density gradient fractions, typically in the first 4–6 sample wells. On the other hand, the NCEM was expected to be found in the higher density gradient fractions, typically in the last 4–6 sample wells.

### Velocity Gradient Centrifugation Isolation of 8S and 70S Complexes of CAV1

Liver and gallbladder tissues and HIBEpiC lines were lysed with 0.5% TritonX‐100 (X100, Sigma‐Aldrich, St. Louis, MO) and a complete protease inhibitor cocktail (11 697 498 001, Roche, Basel, Switzerland) on ice. Postnuclear supernatants were prepared by a 5 min centrifugation at 1500 g (Optima XPN‐100, Beckman Coulter, Brea, CA). Postnuclear supernatants were loaded onto linear 10% to 40% sucrose gradients containing 0.5% TritonX‐100 and protease inhibitor cocktail. After ultracentrifugation (Optima TLX, Beckman Coulter, Brea, CA) for 5 h at 4 °C, 12 fractions (300 µL) of samples were collected from the top to the bottom and analyzed by immunoblotting with an equal loading volume. Then a 12‐well format was used for the western blot analysis of the sucrose gradient fractions. And as previously described,^[^ ^42^ ^]^ the 8S monomeric/oligomeric form of CAV1 was expected to be detected in the lower density fractions, typically in the wells representing the 10–20% sucrose gradient layers. Specifically, it would expected to see the 8S CAV1 band in the first 4–6 wells, covering the lower sucrose concentration range. On the other hand, the 70S high‐molecular‐weight oligomeric/multimeric form of CAV1 was expected to be detected in the higher density fractions, typically in the wells representing the 30–40% sucrose gradient layers. Accordingly, it would expected to see the 70S CAV1 band in the last 4–6 wells, covering the higher sucrose concentration range.

### Electrophoretic Mobility Shift Assay (EMSA)

The ‐19 Sp1 oligonucleotide sequence was 5′‐GGGGAGGGGGCGGAGTTTT‐3′, which were commercially synthesized (Integrated DNA Technology, Coralville, IA). The oligonucleotides (10 µg each) were boiled for 10 min in 10×kinase buffer and allowed to cool gradually to room temperature. The double‐stranded oligonucleotide (200 ng) were 5′‐end‐labeled using T4 kinase (Promega, Madison, WI) and [γ‐^32^P]ATP (MP Biomedicals, Irvine, CA).

Nuclear extract proteins (10 µg) of HIBEpiC lines were added to a 20 µl binding reaction containing HEPES (20 mm, pH 7.9), MgCl_2_ (1 mm), 4% Ficoll, dithiothreitol (0.5 mm), KCl (50 mm), poly dIdC (2 µg; Amersham Biosciences, Amersham, UK), bovine serum albumin (300 µg mL^−1^), and salmon sperm DNA (50 µg mL^−1^) and incubated at 4 °C for 30 min. For supershifts, 2 µg of the Sp1 antibody (Table S4, Supporting Information) was added to the reaction mixture. Labeled probe (1 µL) was added and further incubated for 25 min at 4 °C. A 5% non‐denaturing polyacrylamide gels by electrophoresis with 1×Tris‐borate/EDTA buffer was used to separate the protein‐DNA complexes from unbound probe. The gels were dried and exposed to x‐ray film overnight to visualized the complexes.

### Chromatin Immunoprecipitation‐Quantitative‐PCR (ChIP‐qPCR)

Chromatin from the gallbladder tissues of each experimental mice was sonicated using the Virsonic 60 sonicator (VirTis, Gardiner, NY). Sonicated chromatin was incubated wih 5 µg of the primary antibody, adding 20 µl of Magna ChIP Protein A magnetic beads (16‐661, SigmaAldrich, St. Louis, MO). Antibodies used for immunoprecipitations were as follows: anti‐FXR antibody (sc13063, SantaCruz, Dallas, TX) and anti‐IgG antibody (ab171870, Abcam, Bristol, UK). Samples were incubated in rotation overnight at 4 °C. Beads were washed with low salt buffer, high salt buffer, LiCl buffer, and TE buffer. Subsequent elution and purification of the immunoprecipitated DNA‐proteins complexes was performed using the IPure kit (Diagenode, Denville, NJ). The association of FXR to their respective binding sites of target genes (NFYC gene) was performed by qPCR using the SyberGreen technology (AppliedBiosystems, Foster City, CA) and using sequence‐specific primers (Table S4, Supporting Information) and SyberGreen technology (AppliedBiosystems, Foster City, CA) with normalization to total input DNA. The ChIP‐qPCR data were normalized to total input DNA.

### Statistical Analysis

The data were expressed as the mean ± standard deviation. Statistical analysis was performed by using Prism (GraphPad Software, San Diego, CA). Data normality was determined by using Shapiro‐Wilkes test. Differences (*p* < 0.05 considered significant) between two independent samples with normal distribution were determined by using Student's t‐test, while Mann‐Whitney test was used to compare non‐normal data (*p* < 0.05 considered significant). For more than two normally distributed samples, statistical comparisons were made by ANOVA with Tukey's range test. And Kruskal‐Wallis non‐parametric ANOVA with Benjamini‐Hochberg corrections was used to compare more than two samples with non‐normal distribution. An adjusted *p* <0.05 was considered significant after Tukey's or Benjamini‐Hochberg corrections for multiple comparisons.

### Ethics Approval Statement

The study was reviewed and received approval from the Animal Experimental Ethical Inspection of the First Affiliated Hospital, College of Medicine, Zhejiang University (2020‐1288).

### Patient Consent Statement

Gallbladder samples were obtained with informed consent from patients undergoing laparoscopic cholecystectomy. Prior to surgery, patients agreed to the use of their cholecystectomy specimens for non‐profit scientific research purposes. The use of human gallbladder samples for this study was approved by the Clinical Research Ethics Committee of the First Affiliated Hospital, College of Medicine, Zhejiang University (2020‐936).

## Conflict of Interest

The authors declare no conflict of interest.

## Supporting information



Supporting Information

## Data Availability

The data that support the findings of this study are available in the supplementary material of this article.
