# Supplementary material for: A Gallbladder‐Specific Hydrophobic Bile Acid‐FXR‐MUC1 Signaling Axis Mediates Cholesterol Gallstone Formation
Source: Adv Sci (Weinh). 2025 Feb 11;12(13):2401956. doi: 10.1002/advs.202401956 (PMC11967835; doi:10.1002/advs.202401956)
Supplement: Supplementary file 1 — Supporting Information [file ADVS-12-2401956-s001.pdf]

ADVANCED SCIENCE
Open Access

Supporting Information

for *Adv. Sci.*, DOI 10.1002/advs.202401956

A Gallbladder-Specific Hydrophobic Bile Acid-FXR-MUC1 Signaling Axis Mediates Cholesterol Gallstone Formation

Hongtan Chen, Xin Jiang, Yiqiao Li, Honggang Guo, Jianguo Wu, Sha Li, Fengling Hu and Guoqiang Xu**

Figrure S1

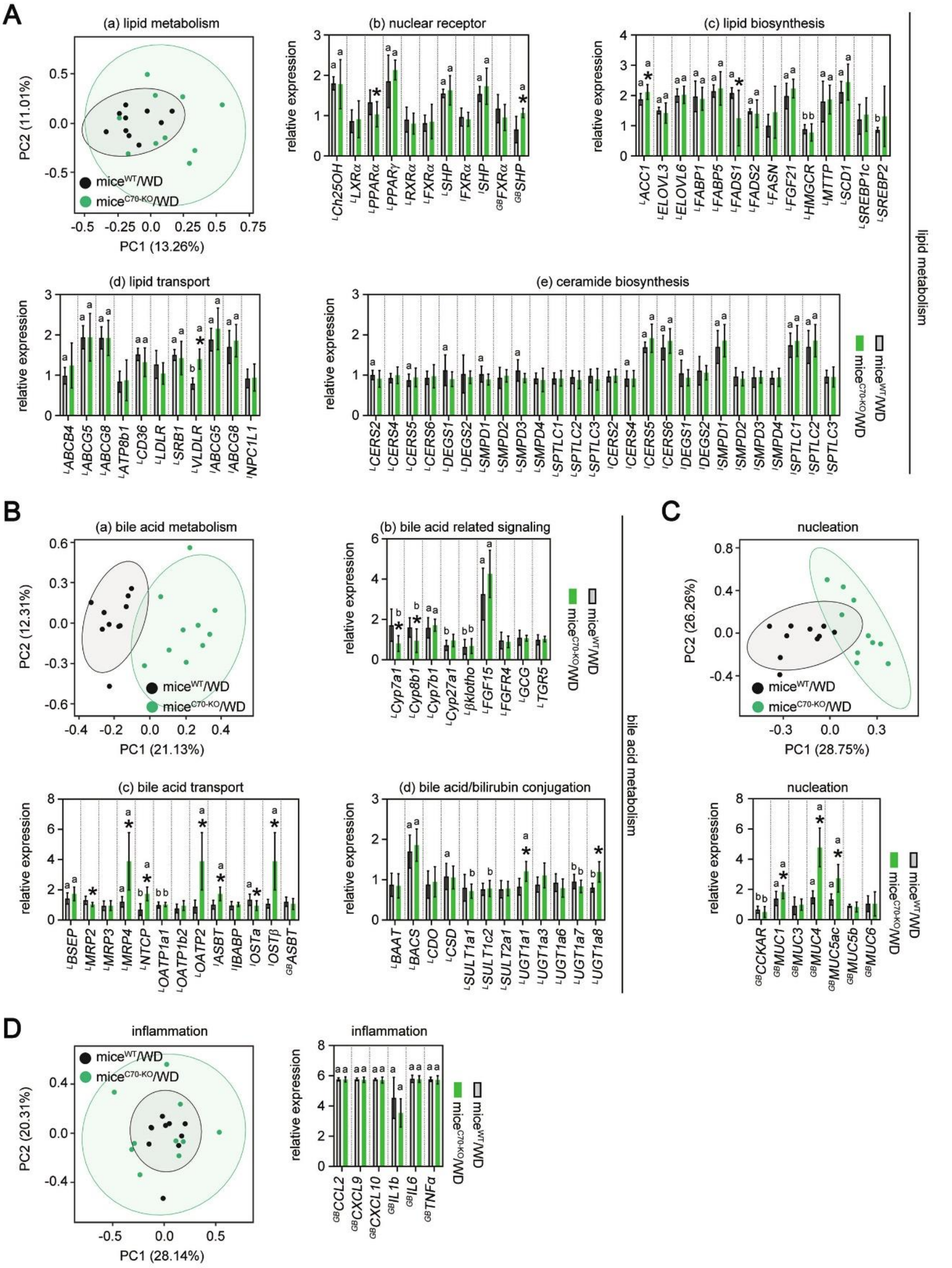
A
(a) lipid metabolism
PC2 (11.01%)
PC1 (13.26%)
mice^WT/WD
mice^C70-KO/WD
(b) nuclear receptor
relative expression
^LCh25OH ^LLXRα ^LPPARα ^LPPARγ ^LRXRα ^LFXRα ^LSHP ^IFXRα ^ISHP ^GBFXRα ^GBSHP
(c) lipid biosynthesis
^LACC1 ^LELOVL3 ^LELOVL6 ^LFABP1 ^LFABP5 ^LFADS1 ^LFADS2 ^LFASN ^LFGF21 ^LHMGCR ^LMTTP ^LSCD1 ^LSREBP1c ^LSREBP2
(d) lipid transport
^LABCB4 ^LABCG5 ^LABCG8 ^LATP8b1 ^LCD36 ^LLDLR ^LSRB1 ^LVLDLR ^IABCG5 ^IABCG8 ^INPC1L1
(e) ceramide biosynthesis
^LCERS2 ^LCERS4 ^LCERS5 ^LCERS6 ^LDEGS1 ^LDEGS2 ^LSMPD1 ^LSMPD2 ^LSMPD3 ^LSMPD4 ^LSPTLC1 ^LSPTLC2 ^LSPTLC3 ^ICERS2 ^ICERS4 ^ICERS5 ^ICERS6 ^IDEGS1 ^IDEGS2 ^ISMPD1 ^ISMPD2 ^ISMPD3 ^ISMPD4 ^ISPTLC1 ^ISPTLC2 ^ISPTLC3
lipid metabolism
B
(a) bile acid metabolism
PC2 (12.31%)
PC1 (21.13%)
(b) bile acid related signaling
^LCyp7a1 ^LCyp8b1 ^LCyp7b1 ^LCyp27a1 ^Lβklotho ^IFGF15 ^LFGFR4 ^IGCG ^LTGR5
(c) bile acid transport
^LBSEP ^LMRP2 ^LMRP3 ^LMRP4 ^LNTCP ^LOATP1a1 ^LOATP1b2 ^LOATP2 ^IASBT ^IIBABP ^IOSTa ^IOSTβ ^GBASBT
(d) bile acid/bilirubin conjugation
^LBAAT ^LBACS ^LCDO ^LCSD ^LSULT1a1 ^LSULT1c2 ^LSULT2a1 ^LUGT1a1 ^LUGT1a3 ^LUGT1a6 ^LUGT1a7 ^LUGT1a8
bile acid metabolism
C
nucleation
PC2 (26.26%)
PC1 (28.75%)
^GBCCKAR ^GBMUC1 ^GBMUC3 ^GBMUC4 ^GBMUC5ac ^GBMUC5b ^GBMUC6
D
inflammation
PC2 (20.31%)
PC1 (28.14%)
^GBCCL2 ^GBCXCL9 ^GBCXCL10 ^GBIL1b ^GBIL6 ^GBTNFα

Figure S1. The lithogenic gene expression profiles of gallbladder, liver, and small intestine collected from mice^{WT} and mice$^{C70\text{-}KO}$ after 8-week WD feeding.

A, PCA plots (a) of the expression patterns of genes associated with lipid metabolism and the mRNA levels (b-e) of genes associated with nuclear receptor (b) (Ch25OH, LXRα, PPARα, PPARγ, RXRα, FXRα, SHP), lipid biosynthesis (c) (ACC1, ELOVL3, ELOVL6, FABP1, FABP5, FADS1, FADS2, FASN, FGF21, HMGCR, MTTP, SCD1, SREBP1c, SREBP2), lipid transport (d) (ABCB4, ABCG5, ABCG8, ATP8b1, CD36, LDLR, SRB1, VLDLR, NPC1L1) and ceramide biosynthesis (e) (CERS2, CERS4, CERS5, CERS6, DEGS1, DEGS2, SMPD1, SMPD2, SMPD3, SMPD4, SPTLC1, SPTLC2, SPTLC3) in liver and small intestine of WD-fed (8-week) mice^{WT} and mice$^{C70\text{-}KO}$.

B, PCA plots (a) of the expression patterns of genes associated with bile acid metabolism and the mRNA levels (b-d) of genes associated with bile acid related signaling (b) (Cyp7a1, Cyp8b1, Cyp27a1, Cyp7b1, βklotho, FGF15, FGFR4, GCG, TGR5), bile acid transport (c) (BSEP, MRP2, MRP3, MRP4, NTCP, OATP1a1, OATP1b2, OATP2, ASBT, IBABP, OSTα, OSTβ) and bile acid/bilirubin conjugation (d) (BAAT, BACS, CDO, CSD, SULT1a1, SULT1c2, SULT2a1, UGT1a1, UGT1a3, UGT1a6, UGT1a7, UGT1a8) in liver, small intestine and gallbladder of WD-fed (8-week) mice^{WT} and mice$^{C70\text{-}KO}$.

C, PCA plots (upper panel) of the expression patterns of genes associated with cholesterol nucleation and the mRNA levels (lower panel) of CCKAR, MUC1, MUC3, MUC4, MUC5ac, MUC5b and MUC6 in gallbladder of WD-fed (8-week) mice^{WT} and mice$^{C70\text{-}KO}$.

D, PCA plots (left panel) of the expression patterns of genes associated with gallbladder inflammation and the mRNA levels (right panel) of CCL2, CXCL9, CXCL10, IL1b, IL6 and TNFα in gallbladder of WD-fed (8-week) mice^{WT} and mice$^{C70\text{-}KO}$.

WD, fed on western diet.

mice^{WT}/WD, WD-fed mice^{WT}; mice$^{C70\text{-}KO}$/WD, WD-fed mice$^{C70\text{-}KO}$.

Expression data were normalized to the expression of 18s RNA.

The superscript "L" corresponds to liver expression, the superscript "GB" corresponds to gallbladder expression, and the superscript "I" corresponds to small intestine expression.

PC1, Principal component 1; PC2, Principal component 2.

Data were presented as mean ± SD.

Data with lowercase letter "a" means a significant elevation (P<0.05) compared to the control (chow vs. WD).

Data with lowercase letter "b" means a significant reduction (P<0.05) compared to the control (chow vs. WD).

*, significant differences (P<0.05) between mice^{WT}/WD and mice$^{C70\text{-}KO}$/WD.

Data of chow-fed mice^{WT} and mice$^{C70\text{-}KO}$ and individual P values were provided in Primary Data for Figure S1.

Figrure S2

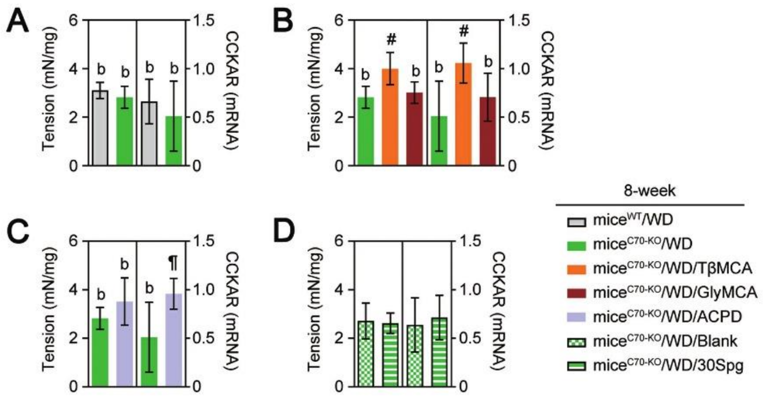
A
B
C
D
Tension (mN/mg)
CCKAR (mRNA)
8-week
mice^{WT}/WD
mice^{C70-KO}/WD
mice^{C70-KO}/WD/TβMCA
mice^{C70-KO}/WD/GlyMCA
mice^{C70-KO}/WD/ACPD
mice^{C70-KO}/WD/Blank
mice^{C70-KO}/WD/30Spg

Figure S2. Myograph studies on the contractility of gallbladder collected from each mice group.

A-D, The maximal tension and the CCKAR mRNA levels were detected in the gallbladder collected from mice^{WT} or mice$^{C70\text{-}KO}$ (A), form mice$^{C70\text{-}KO}$, with or without GlyMCA, TβMCA (B) or ACPD (C) treatment, or from mice$^{C70\text{-}KO}$/Blank and mice$^{C70\text{-}KO}$/30Spg (D), after 8-week WD feeding. mN/mg, millinewtons per milligram; The CCKAR mRNA expression data were normalized to the expression of 18s RNA.

WD, fed on western diet.

mice^{WT}/WD, WD-fed mice^{WT}; mice$^{C70\text{-}KO}$/WD, WD-fed mice$^{C70\text{-}KO}$; mice$^{C70\text{-}KO}$/WD/GlyMCA, GlyMCA-treated (50 mg/kg/day) WD-fed mice$^{C70\text{-}KO}$; mice$^{C70\text{-}KO}$/WD/TβMCA, TβMCA-treated (500 mg/kg/day) WD-fed mice$^{C70\text{-}KO}$; mice$^{C70\text{-}KO}$/WD/ACPD, ACPD-treated (10 mg/kg/day) WD-fed mice$^{C70\text{-}KO}$; mice$^{C70\text{-}KO}$/WD/Blank, AAV-Null injected WD-fed mice$^{C70\text{-}KO}$; mice$^{C70\text{-}KO}$/WD/30Spg, AAV-30Spg injected WD-fed mice$^{C70\text{-}KO}$.

Data were presented as mean ± SD.

Data with lowercase letter "b" means a significant reduction (*P*<0.05) compared to the control (chow vs. WD).

Data with different symbol (#, mice$^{C70\text{-}KO}$/WD/GlyMCA vs. mice$^{C70\text{-}KO}$/WD and mice$^{C70\text{-}KO}$/WD/TβMCA; ¶, mice$^{C70\text{-}KO}$/WD/ACPD vs. mice$^{C70\text{-}KO}$/WD) indicates significant differences (*P*<0.05).

Data of chow-fed mice^{WT} and mice$^{C70\text{-}KO}$ and individual *P* values were provided in Primary Data for Figure S2.

Figrure S3

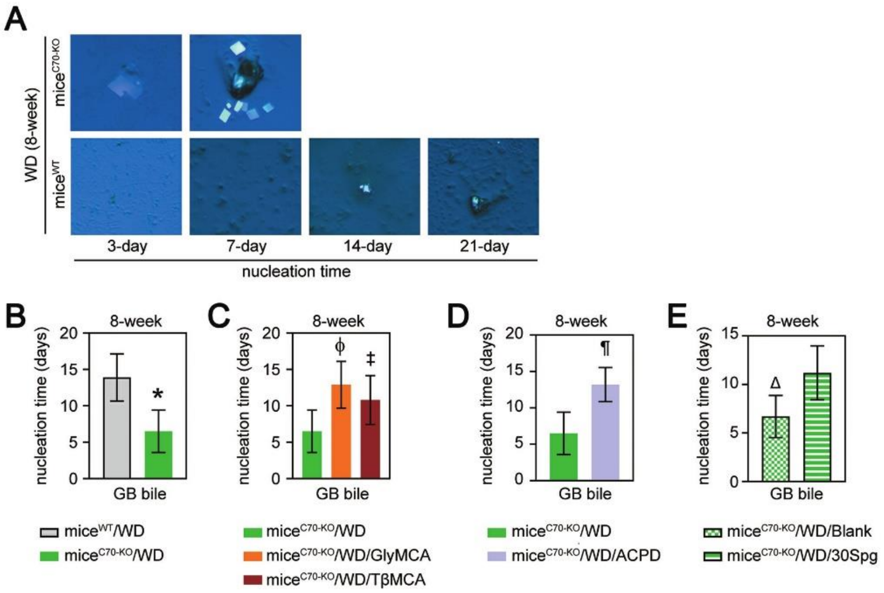
A
WD (8-week)
mice^{C70-KO}
mice^{WT}
3-day
7-day
14-day
21-day
nucleation time
B
C
D
E
8-week
nucleation time (days)
GB bile
mice^{WT}/WD
mice^{C70-KO}/WD
mice^{C70-KO}/WD/GlyMCA
mice^{C70-KO}/WD/TβMCA
mice^{C70-KO}/WD/ACPD
mice^{C70-KO}/WD/Blank
mice^{C70-KO}/WD/30Spg

Figure S3. Nucleation time for cholesterol crystal formation in gallbladder bile collected from each mice group.

A, The gallbladder bile collected from mice$^{\text{C70-KO}}$ and mice^{WT} after 8-week WD feeding were incubated *in vitro* and observed at different day.

B, Nucleation time for cholesterol crystal formation in gallbladder bile collected from mice$^{\text{C70-KO}}$ and mice^{WT} after 8-week WD feeding.

C-D, Effect of either GlyMCA or TβMCA (C) or ACPD (D) treatment on nucleation time for cholesterol crystal formation in gallbladder bile collected from 8-week WD-fed mice$^{\text{C70-KO}}$.

E, Nucleation time for cholesterol crystal formation in gallbladder bile collected from Blank or 30Spg treated mice$^{\text{C70-KO}}$ after 8-week WD feeding.

WD, fed on western diet.

mice^{WT}/WD, WD-fed mice^{WT}; mice$^{\text{C70-KO}}$/WD, WD-fed mice$^{\text{C70-KO}}$; mice$^{\text{C70-KO}}$/WD/GlyMCA, GlyMCA-treated (50 mg/kg/day) WD-fed mice$^{\text{C70-KO}}$; mice$^{\text{C70-KO}}$/WD/TβMCA, TβMCA-treated (500 mg/kg/day) WD-fed mice$^{\text{C70-KO}}$; mice$^{\text{C70-KO}}$/WD/ACPD, ACPD-treated (10 mg/kg/day) WD-fed mice$^{\text{C70-KO}}$; mice$^{\text{C70-KO}}$/WD/Blank, AAV-Null injected WD-fed mice$^{\text{C70-KO}}$; mice$^{\text{C70-KO}}$/WD/30Spg, AAV-30Spg injected WD-fed mice$^{\text{C70-KO}}$.

Data were presented as mean ± SD.

Data with different symbol (*, mice$^{\text{C70-KO}}$ vs. mice^{WT}; φ, mice$^{\text{C70-KO}}$/WD/GlyMCA vs. mice$^{\text{C70-KO}}$/WD; ‡, mice$^{\text{C70-KO}}$/WD/TβMCA vs. mice$^{\text{C70-KO}}$/WD; ¶, mice$^{\text{C70-KO}}$/WD/ACPD vs. mice$^{\text{C70-KO}}$/WD; Δ, mice$^{\text{C70-KO/30Spg}}$/WD vs. mice$^{\text{C70-KO/Blank}}$/WD) indicates significant differences (P<0.05).

Individual *P* values were provided in Primary Data for Figure S3.

Figrure S4

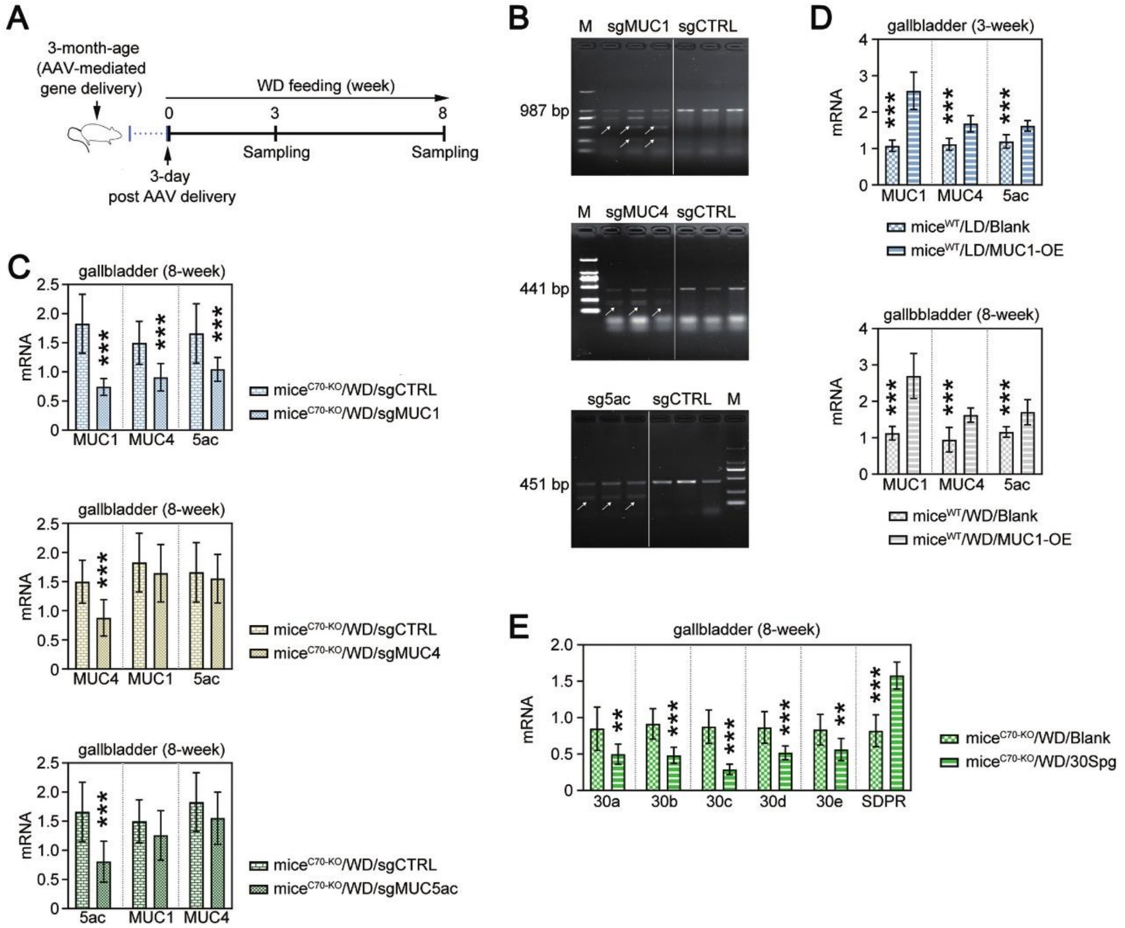
A
3-month-age
(AAV-mediated
gene delivery)
WD feeding (week)
0
3
8
Sampling
Sampling
3-day
post AAV delivery
B
M sgMUC1 sgCTRL
987 bp
M sgMUC4 sgCTRL
441 bp
sg5ac sgCTRL M
451 bp
C
gallbladder (8-week)
mRNA
MUC1 MUC4 5ac
mice^C70-KO/WD/sgCTRL
mice^C70-KO/WD/sgMUC1
MUC4 MUC1 5ac
mice^C70-KO/WD/sgCTRL
mice^C70-KO/WD/sgMUC4
5ac MUC1 MUC4
mice^C70-KO/WD/sgCTRL
mice^C70-KO/WD/sgMUC5ac
D
gallbladder (3-week)
mice^WT/LD/Blank
mice^WT/LD/MUC1-OE
gallbbladder (8-week)
mice^WT/WD/Blank
mice^WT/WD/MUC1-OE
E
gallbladder (8-week)
30a 30b 30c 30d 30e SDPR
mice^C70-KO/WD/Blank
mice^C70-KO/WD/30Spg

Figure S4. Gene Delivery to the mouse gallbladder after local administration of AAV2/8 vectors.

A, Scheme of treatment. Mice^{WT} or mice$^{C70\text{-}KO}$ were administered i.p. injections of AAV2/8-based gene delivery (1 × 10^{12} vector genomes/mouse). On the third day following the injection, mice were fed a LD for 3-week or fed a WD for 8-week.

B, *In vivo* mutation of MUC1, MUC4 and MUC5ac by AAV-Cas9 based gene editing detected in the mice$^{C70\text{-}KO}$. Gel images to detect genome editing. White arrows represent mutation. PCR amplified genomic DNA from isolated gallbladder using a forward primer that anneals upstream of the Cas9-sgRNA target site and a reverse primer that anneals downstream of the Cas9-sgRNA target site. Mice$^{C70\text{-}KO}$ were i.p. injected with (1 × 10^{12} vector genomes/mouse) AAV-Cas9-sgCTRL (sgCTRL), AAV-Cas9-sgMUC1 (sgMUC1), or AAV-Cas9-sgMUC4 (sgMUC4), or AAV-Cas9-sgMUC5ac (sg5ac). M, 2000 bp DNA Marker. i.p., intraperitoneal injection.

C, Expression of MUC1, MUC4 and MUC5ac analysis by qRT-PCR in the gallbladder tissues collected from WD-fed mice$^{C70\text{-}KO}$ with the injection of sgCTRL, sgMUC1, sgMUC4, or sgMUC5ac. 5ac, MUC5ac.

Expression data were normalized to the expression of 18s RNA.

***, $P<0.001$; significant difference between mice$^{C70\text{-}KO}$/WD/sgCTRL vs. mice$^{C70\text{-}KO}$/WD/sgMUC1, or vs. mice$^{C70\text{-}KO}$/WD/sgMUC4, vs. mice$^{C70\text{-}KO}$/WD/sgMUC5ac.

D, Expression of MUC1, MUC4 and MUC5ac analysis by qRT-PCR in the gallbladder tissues collected from 3-week LD-fed mice^{WT} (upper) or 8-week WD-fed mice^{WT} (lower), with the injection of Blank, or MUC1-OE (1 × 10^{12} vector genomes/mouse).

Expression data were normalized to the expression of 18s RNA.

***, $P<0.001$; significant difference between mice^{WT}/LD/Blank vs. mice^{WT}/LD/MUC1-OE, or between mice^{WT}/WD/Blank vs. mice^{WT}/WD/MUC1-OE.

E, Expression of miR30a, miR30b, miR30c, miR30d, miR30e and SDPR analysis by qRT-PCR in the gallbladder tissues collected from WD-fed mice$^{C70\text{-}KO}$, with the injection of Blank, or 30Spg (1 × 10^{12} vector genomes/mouse).

Expression data were normalized to the expression of 18s RNA.

**, $P<0.01$; ***, $P<0.001$; significant difference between mice$^{C70\text{-}KO}$/WD/Blank vs. mice$^{C70\text{-}KO}$/WD/30Spg.

30a, miR30a; 30b, miR30b; 30c, miR30c; 30d, miR30d; 30e, miR30e.

WD, fed on western diet.

mice$^{C70\text{-}KO}$/WD/sgCTRL, AAV-Cas9-sgCTRL injected WD-fed mice$^{C70\text{-}KO}$; mice$^{C70\text{-}}$

^{KO}/WD/sgMUC1, AAV-Cas9-sgMUC1 injected WD-fed mice$^{C70\text{-}KO}$; mice$^{C70\text{-}KO}$/WD/sgMUC4, AAV-Cas9-sgMUC4 injected WD-fed mice$^{C70\text{-}KO}$; mice$^{C70\text{-}KO}$/WD/sgMUC5ac, AAV-Cas9-sgMUC5ac injected WD-fed mice$^{C70\text{-}KO}$; mice$^{C70\text{-}KO}$/LD/Blank, AAV-Null injected LD-fed mice$^{C70\text{-}KO}$; mice$^{C70\text{-}KO}$/LD/MUC1-OE, AAV-MUC1 injected LD-fed mice$^{C70\text{-}KO}$; mice$^{C70\text{-}}$^{KO}/WD/Blank, AAV-Null injected WD-fed mice$^{C70\text{-}KO}$; mice$^{C70\text{-}KO}$/WD/MUC1-OE, AAV-MUC1 injected WD-fed mice$^{C70\text{-}KO}$; mice$^{C70\text{-}KO}$/WD/30Spg, AAV-30Spg injected WD-fed mice$^{C70\text{-}KO}$.

Data were presented as mean ± SD.

Individual *P* values were provided in Primary Data for Figure S4.

Figrure S5

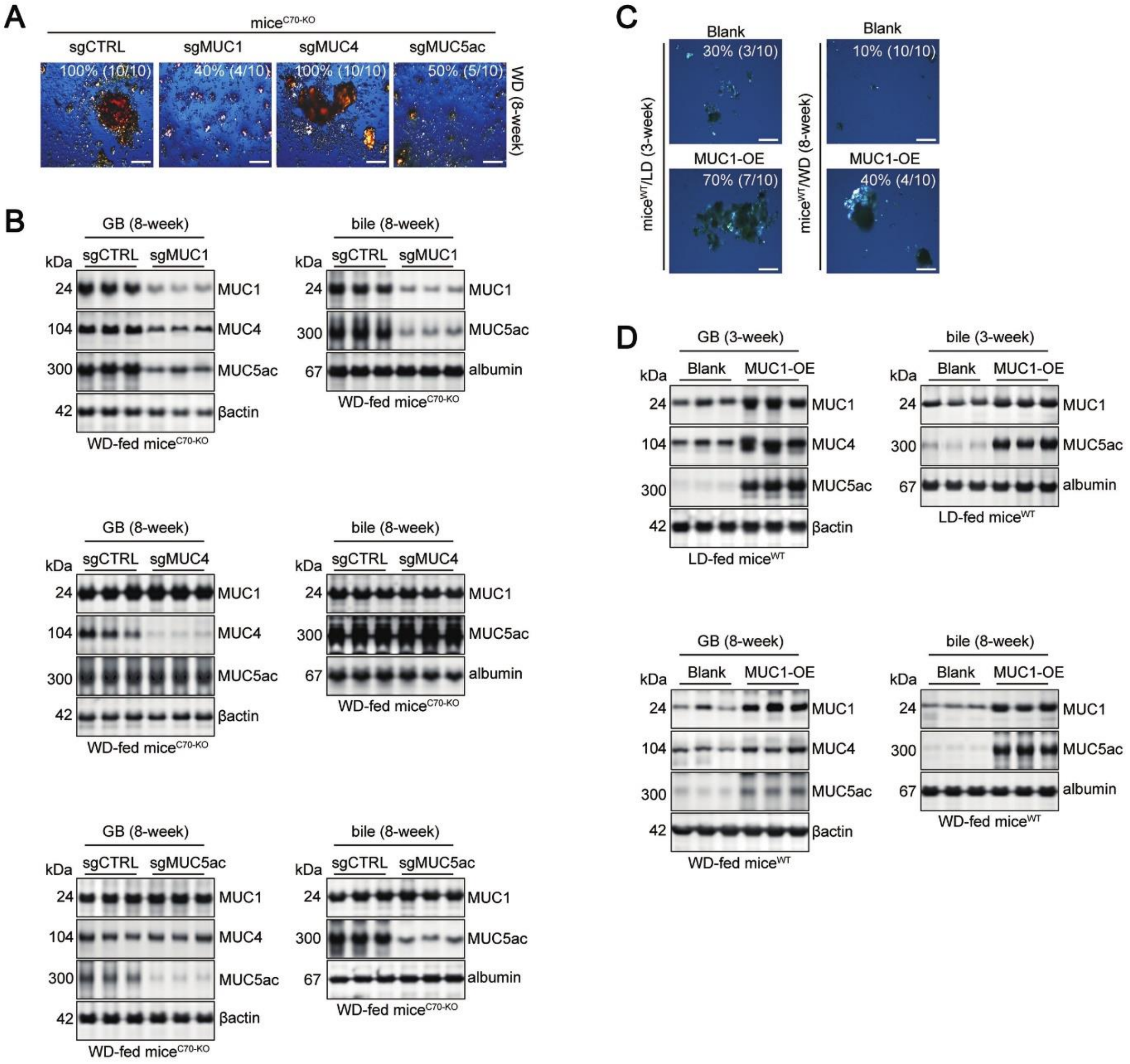
A
mice^{C70-KO}
sgCTRL
sgMUC1
sgMUC4
sgMUC5ac
100% (10/10)
40% (4/10)
100% (10/10)
50% (5/10)
WD (8-week)
B
GB (8-week)
bile (8-week)
sgCTRL
sgMUC1
sgMUC4
sgMUC5ac
kDa
MUC1
MUC4
MUC5ac
βactin
albumin
WD-fed mice^{C70-KO}
C
Blank
MUC1-OE
30% (3/10)
70% (7/10)
10% (10/10)
40% (4/10)
mice^{WT}/LD (3-week)
mice^{WT}/WD (8-week)
D
GB (3-week)
bile (3-week)
GB (8-week)
bile (8-week)
LD-fed mice^{WT}
WD-fed mice^{WT}

Figure S5. The role of MUC1, MUC4 or MUC5ac on CGD development.

A, The polarizing light microscopy examination of cholesterol crystals (scale bar: 100 μm), collected from mice$^{C70\text{-}KO}$ after 8-week WD feeding, with the treatment of sgCTRL, sgMUC1, sgMUC4, or sgMUC5ac.

B, WB analysis of MUC1, MUC4 and MUC5ac expression in the gallbladder tissues (left panel) and gallbladder bile (right panel) collected from mice$^{C70\text{-}KO}$ after 8-week WD feeding, with the i.p. injection of sgCTRL, sgMUC1, sgMUC4, or sgMUC5ac. The expression of βactin or albumin was used as a loading internal control for cytoplasmic or bile extracts, respectively.

C, The polarizing light microscopy examination of cholesterol crystals (scale bar: 100 μm), collected from mice^{WT} after 3-week LD (left) or 8-week WD (right) feeding, with the treatment of Blank or MUC1-OE.

D, WB analysis of MUC1, MUC4 and MUC5ac expression in the gallbladder tissues (left panel) and gallbladder bile (right panel) collected from mice^{WT} after 3-week LD or 8-week WD feeding, with the i.p. injection of Blank or MUC1-OE. The expression of βactin or albumin was used as a loading internal control for cytoplasmic or bile extracts, respectively.

GB, gallbladder; bile, gallbladder bile.

WD, fed on western diet; LD, fed on lithogenic diet.

Blank, AAV-Null injection; MUC1-OE, AAV-MUC1 injection; sgCTRL, AAV-Cas9-sgCTRL injection; sgMUC1, AAV-Cas9-sgMUC1 injection; sgMUC4, AAV-Cas9-sgMUC4 injection; sgMUC5ac, AAV-Cas9-sgMUC5ac injection.

Figrure S6

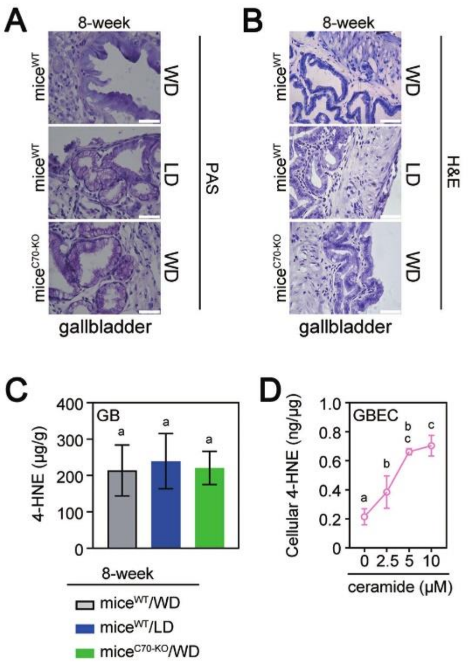

A
8-week
mice^{WT}
WD
mice^{WT}
LD
mice^{C70-KO}
WD
PAS
gallbladder
B
8-week
mice^{WT}
WD
mice^{WT}
LD
mice^{C70-KO}
WD
H&E
gallbladder
C
GB
4-HNE (μg/g)
400
300
200
100
0
a
a
a
8-week
mice^{WT}/WD
mice^{WT}/LD
mice^{C70-KO}/WD
D
GBEC
Cellular 4-HNE (ng/μg)
1.0
0.8
0.6
0.4
0.2
0
a
b
b c
c
0 2.5 5 10
ceramide (μM)

Figure S6. The change of 4-HNE contents in ceramide-treated cells and in the GB of WD-fed mice^{WT} or mice$^{C70\text{-}KO}$.

A and B, PAS (A) and H&E (B) staining of gallbladder sections from 8-week WD-fed mice^{WT}, 8-week LD-fed mice^{WT}, 8-week WD-fed mice$^{C70\text{-}KO}$ (scale bar: 100 μm).

C, The change of 4-HNE contents in the gallbladder of mice^{WT} or mice$^{C70\text{-}KO}$ after 8-week WD feeding.

GB, gallbladder.

WD, fed on western diet.

mice^{WT}/WD, WD-fed mice^{WT}; mice$^{C70\text{-}KO}$/WD, WD-fed mice$^{C70\text{-}KO}$.

Data with lowercase letter "a" means a significant elevation (P<0.05) compared to the control (chow vs. WD).

Data of chow-fed mice^{WT} and mice$^{C70\text{-}KO}$ were provided in Primary Data.

D, The change of 4-HNE activity in GBEC *in vitro* after different dose of ceramide stimulation.

GBEC, gallbladder epithelial cells (HIBEpiC).

Data with different lowercase letter indicates significant differences between each dose.

Individual *P* values were provided in Primary Data for Figure S6.

Figrure S7

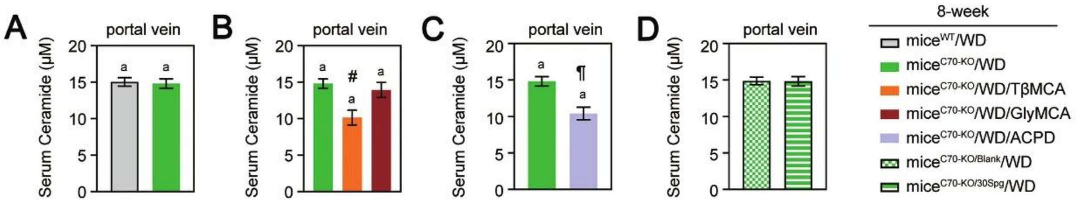
A
B
C
D
portal vein
Serum Ceramide (μM)
20
15
10
5
0
a
#
¶
8-week
miceWT/WD
miceC70-KO/WD
miceC70-KO/WD/TβMCA
miceC70-KO/WD/GlyMCA
miceC70-KO/WD/ACPD
miceC70-KO/Blank/WD
miceC70-KO/30Spg/WD

Figure S7. The detection of serum ceramide levels in samples collected from each mice group.

A-C, The serum ceramide levels in mice^{WT} or mice$^{C70\text{-}KO}$ after 8-week WD feeding (A), with or without GlyMCA, TβMCA (B) or ACPD (C) treatment.

D, The serum ceramide levels in mice$^{C70\text{-}KO/Blank}$ or mice$^{C70\text{-}KO/30Spg}$ after 8-week WD feeding.

WD, fed on western diet.

mice^{WT}/WD, WD-fed mice^{WT}; mice$^{C70\text{-}KO}$/WD, WD-fed mice$^{C70\text{-}KO}$; mice$^{C70\text{-}KO}$/WD/GlyMCA, GlyMCA-treated (50 mg/kg/day) WD-fed mice$^{C70\text{-}KO}$; mice$^{C70\text{-}KO}$/WD/TβMCA, TβMCA-treated (500 mg/kg/day) WD-fed mice$^{C70\text{-}KO}$; mice$^{C70\text{-}KO}$/WD/ACPD, ACPD-treated (10 mg/kg/day) WD-fed mice$^{C70\text{-}KO}$; mice$^{C70\text{-}KO}$/WD/Blank, AAV-Null injected WD-fed mice$^{C70\text{-}KO}$; mice$^{C70\text{-}KO}$/WD/30Spg, AAV-30Spg injected WD-fed mice$^{C70\text{-}KO}$.

Data were presented as mean ± SD.

Data with lowercase letter "a" means a significant elevation (P<0.05) compared to the control (chow vs. WD).

Data with different symbol (#, mice$^{C70\text{-}KO}$/WD/GlyMCA vs. mice$^{C70\text{-}KO}$/WD and mice$^{C70\text{-}KO}$/WD/TβMCA; ¶, mice$^{C70\text{-}KO}$/WD/ACPD vs. mice$^{C70\text{-}KO}$/WD) indicates significant differences (P<0.05).

Data of chow-fed mice^{WT} and mice$^{C70\text{-}KO}$ and individual P values were provided in Primary Data for Figure S7.

Figrure S8

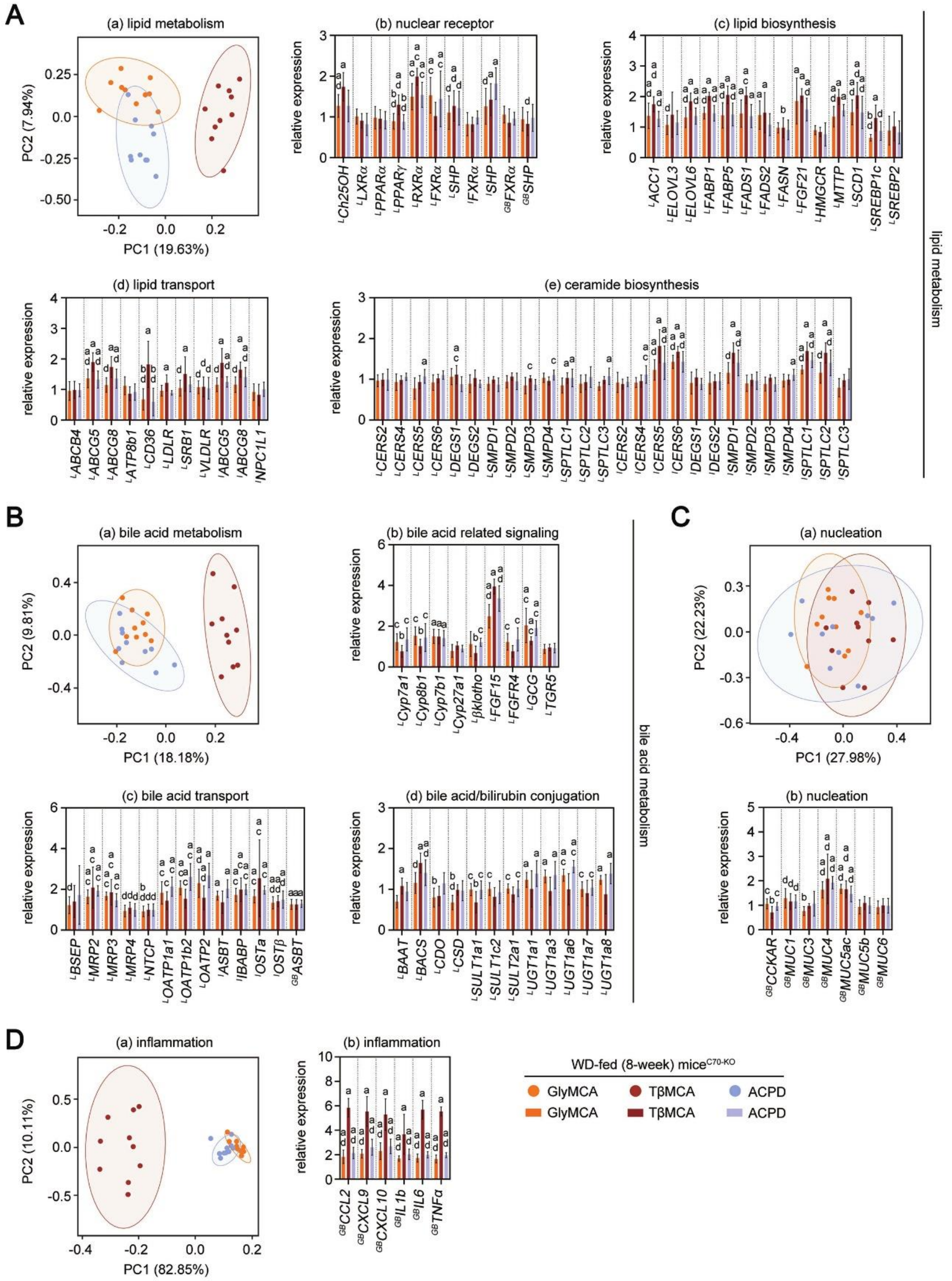
A
(a) lipid metabolism
(b) nuclear receptor
(c) lipid biosynthesis
(d) lipid transport
(e) ceramide biosynthesis
lipid metabolism
B
(a) bile acid metabolism
(b) bile acid related signaling
(c) bile acid transport
(d) bile acid/bilirubin conjugation
bile acid metabolism
C
(a) nucleation
(b) nucleation
D
(a) inflammation
(b) inflammation
relative expression
WD-fed (8-week) mice C70-KO
GlyMCA
TβMCA
ACPD

Figure S8. The lithogenic gene expression profiles of gallbladder, liver, and small intestine collected from mice$^{C70\text{-}KO}$ after 8-week WD feeding.

A, PCA plots (a) of the expression patterns of genes associated with lipid metabolism and the mRNA levels (b-e) of genes associated with nuclear receptor (b) (Ch25OH, LXRα, PPARα, PPARγ, RXRα, FXRα, SHP), lipid biosynthesis (c) (ACC1, ELOVL3, ELOVL6, FABP1, FABP5, FADS1, FADS2, FASN, FGF21, HMGCR, MTTP, SCD1, SREBP1c, SREBP2), lipid transport (d) (ABCB4, ABCG5, ABCG8, ATP8b1, CD36, LDLR, SRB1, VLDLR, NPC1L1) and ceramide biosynthesis (e) (CERS2, CERS4, CERS5, CERS6, DEGS1, DEGS2, SMPD1, SMPD2, SMPD3, SMPD4, SPTLC1, SPTLC2, SPTLC3) in liver and small intestine of WD-fed (8-week) mice$^{C70\text{-}KO}$, with or without GlyMCA, TβMCA or ACPD treatment.

B, PCA plots (a) of the expression patterns of genes associated with bile acid metabolism and the mRNA levels of genes (b-d) associated with bile acid related signaling (b) (Cyp7a1, Cyp8b1, Cyp27a1, Cyp7b1, βklotho, FGF15, FGFR4, GCG, TGR5), bile acid transport (c) (BSEP, MRP2, MRP3, MRP4, NTCP, OATP1a1, OATP1b2, OATP2, ASBT, IBABP, OSTα, OSTβ) and bile acid/bilirubin conjugation (d) (BAAT, BACS, CDO, CSD, SULT1a1, SULT1c2, SULT2a1, UGT1a1, UGT1a3, UGT1a6, UGT1a7, UGT1a8) in liver, small intestine and gallbladder of WD-fed (8-week) mice$^{C70\text{-}KO}$, with or without GlyMCA, TβMCA or ACPD treatment.

C, PCA plots (a) of the expression patterns of genes associated with cholesterol nucleation and the mRNA levels (b) of CCKAR, MUC1, MUC3, MUC4, MUC5ac, MUC5b and MUC6 in gallbladder of WD-fed (8-week) mice$^{C70\text{-}KO}$, with or without GlyMCA, TβMCA or ACPD treatment.

D, PCA plots (a) of the expression patterns of genes associated with gallbladder inflammationand the mRNA levels (b) of CCL2, CXCL9, CXCL10, IL1b, IL6 and TNFα in gallbladder of WD-fed (8-week) mice$^{C70\text{-}KO}$, with or without GlyMCA, TβMCA or ACPD treatment.

WD, fed on western diet; GlyMCA, under GlyMCA (50 mg/kg/day) treatment; TβMCA, under TβMCA (500 mg/kg/day) treatment; ACPD, under ACPD (10 mg/kg/day) treatment.

mice$^{C70\text{-}KO}$/WD, WD-fed mice$^{C70\text{-}KO}$; mice$^{C70\text{-}KO}$/WD/GlyMCA, GlyMCA-treated (50 mg/kg/day) WD-fed mice$^{C70\text{-}KO}$; mice$^{C70\text{-}KO}$/WD/TβMCA, TβMCA-treated (500 mg/kg/day) WD-fed mice$^{C70\text{-}KO}$; mice$^{C70\text{-}KO}$/WD/ACPD, ACPD-treated (10 mg/kg/day) WD-fed mice$^{C70\text{-}KO}$.

Expression data were normalized to the expression of 18s RNA.

The superscript "L" corresponds to liver expression, the superscript "GB" corresponds to

gallbladder expression, and the superscript "I" corresponds to small intestine expression.

PC1, Principal component 1; PC2, Principal component 2.

Data were presented as mean ± SD.

Data with lowercase letter "a" means a significant elevation (*P*<0.05) compared to the control (chow vs. WD).

Data with lowercase letter "b" means a significant reduction (*P*<0.05) compared to the control (chow vs. WD).

Data with lowercase letter "c" means a significant elevation (*P*<0.05) compared to WD-fed mice$^{C70\text{-}KO}$ without treatment.

Data with lowercase letter "d" means a significant reduction (*P*<0.05) compared to WD-fed mice$^{C70\text{-}KO}$ without treatment.

Data of chow-fed mice$^{C70\text{-}KO}$ and individual *P* values were provided in Primary Data for Figure S8

Figrure S9

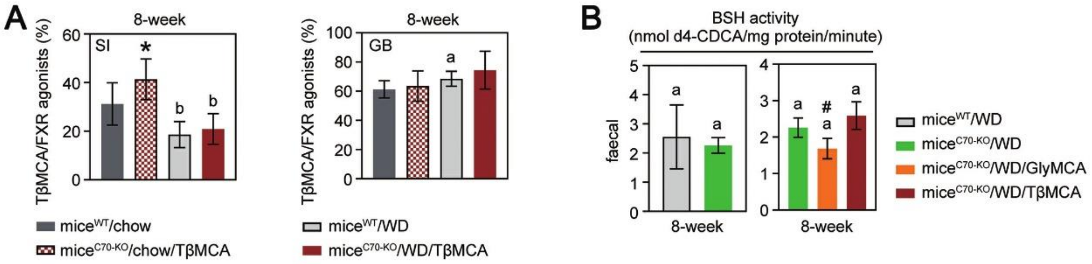
A
8-week
SI
GB
TβMCA/FXR agonists (%)
mice^WT/chow
mice^C70-KO/chow/TβMCA
mice^WT/WD
mice^C70-KO/WD/TβMCA
B
BSH activity
(nmol d4-CDCA/mg protein/minute)
faecal
8-week
mice^WT/WD
mice^C70-KO/WD
mice^C70-KO/WD/GlyMCA
mice^C70-KO/WD/TβMCA

Figure S9. The change of the composition ratio of FXR agonists and TβMCA in BA contents in the SI and GB tissues and BSH activity in the feces of mice$^{C70\text{-}KO}$ during WD feeding.

A, The composition ratio of FXR agonists and of BA content in the SI and GB.

B, The evaluation of fecal BSH enzyme activity by detecting the generation (Per minute) of d4-CDCA (nmol) from d4-TCDCA (nmol) by BSH proteins (mg)/nmol d4-CDCA Per mg of protein per minute).

SI, small intestine; GB, gallbladder.

chow, fed on chow diet; WD, fed on western diet.

mice$^{C70\text{-}KO}$/chow, chow-fed mice$^{C70\text{-}KO}$; mice$^{C70\text{-}KO}$/chow/TβMCA, TβMCA-treated (500 mg/kg/day) chow-fed mice$^{C70\text{-}KO}$; mice^{WT}/WD, WD-fed mice^{WT}; mice$^{C70\text{-}KO}$/WD, WD-fed mice$^{C70\text{-}KO}$; mice$^{C70\text{-}KO}$/WD/GlyMCA, GlyMCA-treated (50 mg/kg/day) WD-fed mice$^{C70\text{-}KO}$; mice$^{C70\text{-}KO}$/WD/TβMCA, TβMCA-treated (500 mg/kg/day) WD-fed mice$^{C70\text{-}KO}$.

Data with lowercase letter "a" means a significant elevation (*P*<0.05) compared to the control (chow vs. WD).

Data with lowercase letter "b" means a significant reduction (*P*<0.05) compared to the control (chow vs. WD).

Data with different symbol (*, mice$^{C70\text{-}KO}$/chow vs. mice$^{C70\text{-}KO}$/chow/TβMCA; #, mice$^{C70\text{-}KO}$/WD/GlyMCA vs. mice$^{C70\text{-}KO}$/WD and mice$^{C70\text{-}KO}$/WD/TβMCA) indicates significant differences (*P*<0.05).

Individual *P* values were provided in Primary Data for Figure S9.

Figrure S10

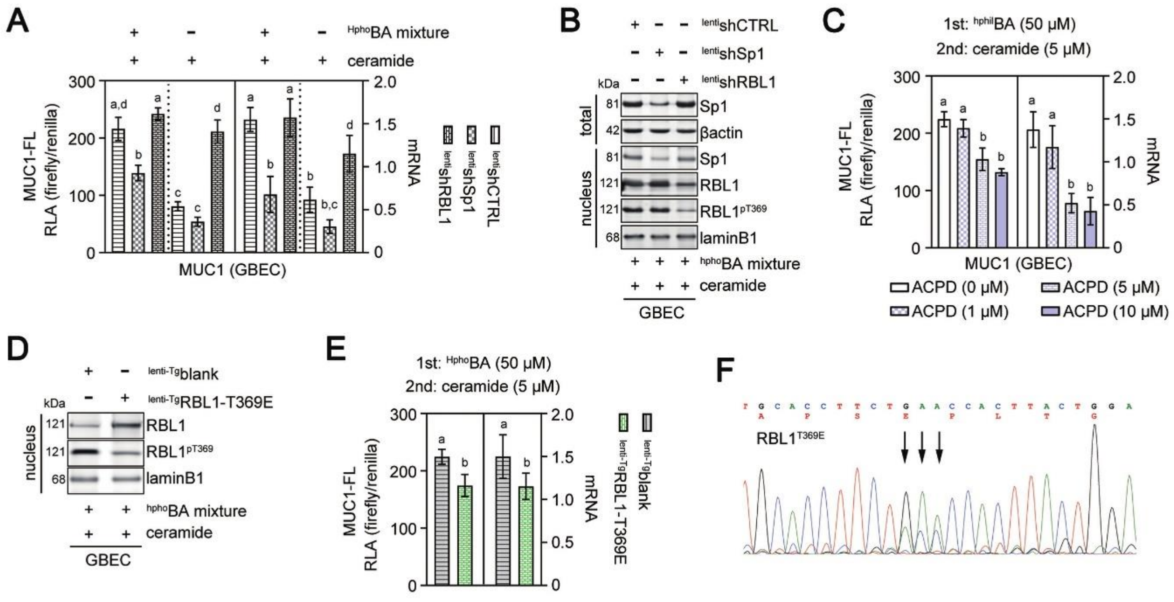

A
HphoBA mixture
ceramide
MUC1-FL RLA (firefly/renilla)
mRNA
lentishRBL1
lentishSp1
lentishCTRL
MUC1 (GBEC)
B
lentishCTRL
lentishSp1
lentishRBL1
kDa
total
nucleus
Sp1
βactin
RBL1
RBL1pT369
laminB1
hphoBA mixture
ceramide
GBEC
C
1st: hphilBA (50 μM)
2nd: ceramide (5 μM)
MUC1-FL RLA (firefly/renilla)
mRNA
MUC1 (GBEC)
ACPD (0 μM)
ACPD (1 μM)
ACPD (5 μM)
ACPD (10 μM)
D
lenti-Tgblank
lenti-TgRBL1-T369E
kDa
nucleus
RBL1
RBL1pT369
laminB1
hphoBA mixture
ceramide
GBEC
E
1st: HphoBA (50 μM)
2nd: ceramide (5 μM)
MUC1-FL RLA (firefly/renilla)
mRNA
lenti-TgRBL1-T369E
lenti-Tgblank
F
RBL1T369E

Figure S10. The change of PKCζ-RBL1-SP1 axis activity on the transcription of MUC1 gene *in vitro*.

A, Left panel: GBEC were stably expressing lentivirus carrying shCTRL (^{lenti}shCTRL), ^{lenti}shSP1 or ^{lenti}shRBL1. Each group of cells were also transduced with lentivirus expressing MUC1 gene promoter LUC reporter (1×10^8 VGP). After 24-hour following lentiviral transduction, the cells were treated with or without 50 μM ^{Hpho}BA mixture for 24-hour, and then were stimulated with 5 μM ceramide for 2-hour. RLA was normalized to β-galactosidase to correct for transfection efficiency. VGP, viral genome particles.

Right panel: GBEC were stably expressing lentivirus carrying shCTRL (^{lenti}shCTRL), ^{lenti}shSP1 or ^{lenti}shRBL1. Each group of cells were treated with or without 50 μM ^{Hpho}BA mixture for 24-hour, and then were stimulated with 5 μM ceramide for 2-hour. Levels of MUC1 mRNA in each group of GBEC were detected by qRT-PCR. Expression data were normalized to the expression of 18s RNA.

The compositions of ^{Hpho}BA mixture used in these *in vitro* studies were shown in Table S3.

B, B, GBEC were stably expressing lentivirus carrying shCTRL (^{lenti}shCTRL), ^{lenti}shSP1 or ^{lenti}shRBL1. WB analysis of Sp1, RBL1 and phosphorylated RBL1 (RBL1^{pT369}) expression in ^{Hpho}BA mixture (50 μM) and ceramide (5 μM) treated GBEC. The expression of βactin or laminA was used as a loading internal control for total or nuclear extracts, respectively.

The compositions of ^{Hpho}BA mixture used in these *in vitro* studies were shown in Table S3.

C, Left panel: GBEC were transduced with lentivirus expressing MUC1 gene promoter LUC reporter (1×10^8 VGP). At 24-hour following transfection, cells cells were treated for 24-hour with 50 μM ^{Hpho}BA mixture, and then were stimulated with 5 μM ceramide, or 5 μM ceramide plus different doses of ACPD (1 μM to 10 μM) for 2-hour. RLA was normalized to β-galactosidase to correct for transfection efficiency. VGP, viral genome particles.

Right panel: GBEC were treated for 24-hour with 50 μM ^{Hpho}BA mixture, and then were stimulated with 5 μM ceramide, or 5 μM ceramide plus different doses of ACPD (1 μM to 10 μM) for 2-hour. Levels of MUC1 mRNA in each group of GBEC were detected by qRT-PCR. Expression data were normalized to the expression of 18s RNA.

The compositions of ^{Hpho}BA mixture used in these *in vitro* studies were shown in Table S3.

D, GBEC were stably expressing lentivirus carrying blank vector ($^{lenti\text{-}Tg}$Blank) or $^{lenti\text{-}Tg}$RBL1-T369E. WB analysis of RBL1 and phosphorylated RBL1 (RBL1^{pT369}) expression in ^{Hpho}BA mixture (50 μM) and ceramide (5 μM) treated GBEC. The expression of laminA was used as a loading internal control for nuclear extracts.

The compositions of ^{Hpho}BA mixture used in these *in vitro* studies were shown in Table S3.

E, Left panel: GBEC were stably expressing lentivirus carrying blank vector ($^{\text{lenti-Tg}}$Blank) or $^{\text{lenti-Tg}}$RBL1-T369E. Each group of cells were also transduced with lentivirus expressing MUC1 gene promoter LUC reporter (1 × 10^8 VGP). After 24-hour following lentiviral transduction, cells were treated with 50 μM ^{Hpho}BA mixture for 24-hour, and then were stimulated with 5 μM ceramide for 2-hour. RLA was normalized to β-galactosidase to correct for transfection efficiency. VGP, viral genome particles.

Right panel: GBEC were stably expressing lentivirus carrying blank vector ($^{\text{lenti-Tg}}$Blank) or $^{\text{lenti-Tg}}$RBL1-T369E. Each group of cells were treated with 50 μM ^{Hpho}BA mixture for 24-hour, and then were stimulated with 5 μM ceramide for 2-hour. Levels of MUC1 mRNA in each group of GBEC were detected by qRT-PCR. Expression data were normalized to the expression of 18s RNA.

The compositions of ^{Hpho}BA mixture used in these *in vitro* studies were shown in Table S3.

F, The construction of $^{\text{lenti- Tg}}$RBL1-T369E vector. The chromatogram of RBL1 gene showing the heterozygous c.1105G>A c.1106A>C and c.1107A>C (arrow) mutation (RBL1 with threonine residues at Positions 369 rePlaced by glutamic acid) *in vitro*.

GBEC, gallbladder epithelial cells (HIBEpiC).

RLA, Relative luciferase activity.

Data were presented as mean ± SD.

Data with different lowercase letter indicates significant differences (P<0.05) between each assigned GBEC group.

Individual *P* values were provided in Primary Data for Figure S10.

Figure S11

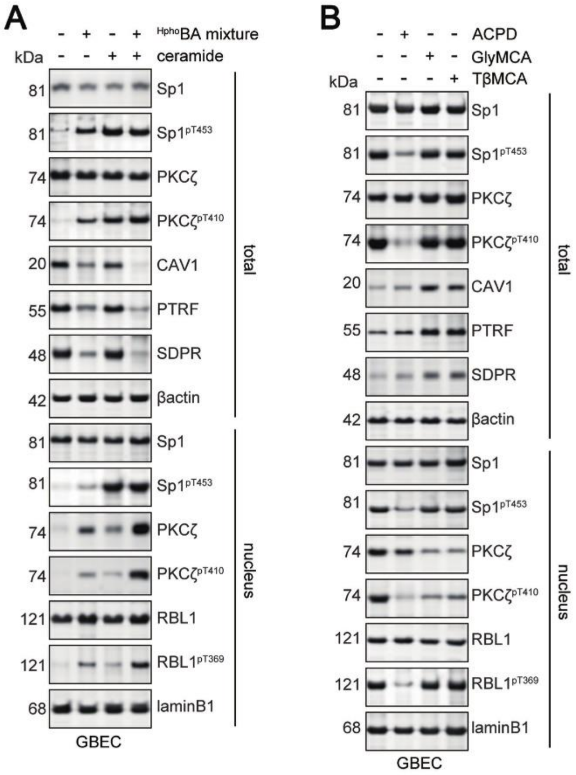
A
HphoBA mixture
ceramide
kDa
Sp1
Sp1pT453
PKCζ
PKCζpT410
CAV1
PTRF
SDPR
βactin
RBL1
RBL1pT369
laminB1
total
nucleus
GBEC
B
ACPD
GlyMCA
TβMCA

Figure S11. Effect of ^{Hpho}BA mixture and ceramide treatment on the activation of PKCζ-RBL1-Sp1 axis and the expression of caveolae constituents in GBEC.

p, phoshorylated; GBEC, gallbladder epithelial cells (HIBEpiC).

A, WB analysis of Sp1 and its phosphorylated form pT453, PKCζ and its phosphorylated form pT410, RBL1 and its phosphorylated form pT369, CAV1, PTRF and SDPR expression in GBEC without or with the treatment of ^{Hpho}BA mixture (50 μM), ceramide (5 μM) or both.

B, WB analysis of Sp1 and its phosphorylated form pT453, PKCζ and its phosphorylated form pT410, RBL1 and its phosphorylated form pT369, CAV1, PTRF and SDPR protein expression in ^{Hpho}BA mixture (50 μM) and ceramide (5 μM) treated GBEC without or with ACPD (5 μM), GlyMCA (50 μM), TβMCA (200 μM) treatment.

The expression of βactin or laminB1 was used as a loading internal control for total or nuclear extracts, respectively.

Figure S12

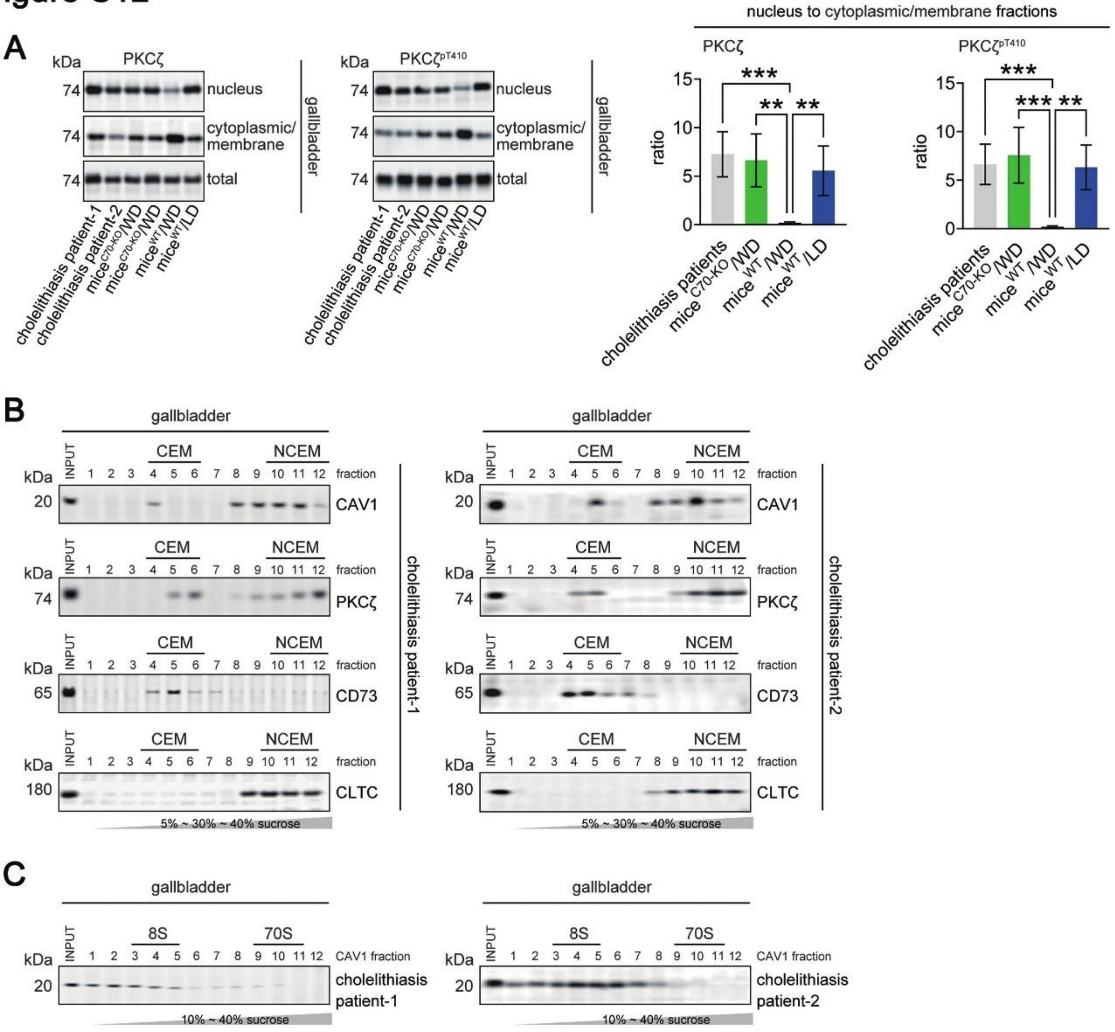
A
kDa
PKCζ
PKCζpT410
74
nucleus
cytoplasmic/
membrane
total
gallbladder
cholelithiasis patient-1
cholelithiasis patient-2
miceC70-KO/WD
miceWT/WD
miceWT/LD
nucleus to cytoplasmic/membrane fractions
ratio
15
10
5
0
***
**
cholelithiasis patients
B
gallbladder
INPUT
CEM
NCEM
fraction
1 2 3 4 5 6 7 8 9 10 11 12
20
CAV1
74
PKCζ
65
CD73
180
CLTC
5% ~ 30% ~ 40% sucrose
cholelithiasis patient-1
cholelithiasis patient-2
C
8S
70S
CAV1 fraction
10% ~ 40% sucrose

Figure S12. The contents of PKCζ in caveolae distribution and the presence of 8S-CAV1 complexes in the gallbladder of cholelithiasis patient.

A, WB analysis (left panel) and quantification (right panel) of PKCζ and its phosphorylated form pT410 were performed on the cytoplasmic/membrane, nuclear, and total fractions of gallbladder lysates collected from cholelithiasis patients, mice$^{C70\text{-}KO}$/WD, mice^{WT}/WD and mice^{WT}/LD. The total fractions represent the sum of the cytoplasmic/membrane and nuclear fractions. The intensities of the PKCζ bands from the cytoplasmic/membrane or nuclear fractions were normalized to the band intensities from the total fractions, which served as an internal control. ImageJ was used to analyze the grayscale values of the PKCζ protein bands. The ratios of cytoplasmic/membrane to nuclear fractions of PKCζ were compared among the four groups.

Data with the following symbols indicate significant differences between each group: **, P<0.01; **, P<0.001.

Individual P values were provided in Primary Data for Figure S12.

B, The sucrose linear (5%–30%-40%) gradients were performed to isolate CEM (fractions 4 to 6) or NCEM (fractions 10 to 12) fractions from the gallbladder of cholelithiasis patient.

CAV1 and PKCζ are mainly localized in the NCEM of the gallbladder of cholelithiasis patient. CD73 was used as CEM subcellular markers, and CLTC (clarthin heavy chain) was used as NCEM subcellular markers.

C, The sucrose linear (10%-40%) gradients were performed to isolate 8S-CAV1 (fractions 3 to 5) and 70S-CAV1 (fractions 9 to 11) complexes in the gallbladder of cholelithiasis patient.

Figure S13

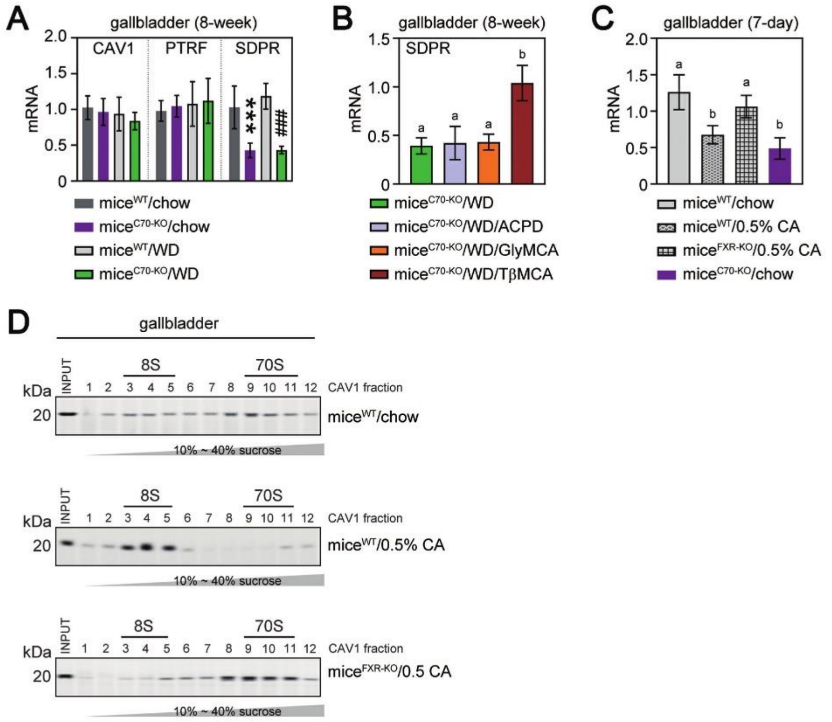
A
gallbladder (8-week)
CAV1
PTRF
SDPR
mRNA
2.0
1.5
1.0
0.5
0
***
###
miceWT/chow
miceC70-KO/chow
miceWT/WD
miceC70-KO/WD
B
gallbladder (8-week)
SDPR
mRNA
1.5
1.0
0.5
0
a
b
miceC70-KO/WD
miceC70-KO/WD/ACPD
miceC70-KO/WD/GlyMCA
miceC70-KO/WD/TβMCA
C
gallbladder (7-day)
mRNA
2.0
1.5
1.0
0.5
0
a
b
miceWT/chow
miceWT/0.5% CA
miceFXR-KO/0.5% CA
miceC70-KO/chow
D
gallbladder
INPUT
8S
70S
kDa
1 2 3 4 5 6 7 8 9 10 11 12
CAV1 fraction
20
miceWT/chow
10% ~ 40% sucrose
miceWT/0.5% CA
miceFXR-KO/0.5 CA

Figure S13. The reduced SDPR expression in the gallbladder of mice$^{C70\text{-}KO}$ via FXR.

A, Levels of CAV1, PTRF and SDPR mRNA in the gallbladder mice$^{C70\text{-}KO}$ and mice^{WT} were detected by qRT-PCR. Expression data were normalized to the expression of 18s RNA.

*, significant differences (P<0.05) between mice^{WT}/chow vs. mice$^{C70\text{-}KO}$/chow.

#, significant differences (P<0.05) between mice^{WT}/WD vs. mice$^{C70\text{-}KO}$/WD.

B, Levels of SDPR mRNA in the gallbladder of WD-fed mice$^{C70\text{-}KO}$, with or without ACPD, GlyMCA or TβMCA treatment, were detected by qRT-PCR. Expression data were normalized to the expression of 18s RNA.

Data with different lowercase letter indicates significant differences (P<0.05) between each mice group.

Δ, significant differences (P<0.05) between mice$^{C70\text{-}KO}$/chow vs. mice$^{C70\text{-}KO}$/WD/TβMCA.

C, Levels of SDPR mRNA in the gallbladder of chow-fed mice^{WT}, 0.5% CA (7-day) fed mice^{WT} or 0.5% CA (7-day) fed mice$^{FXR\text{-}KO}$, were detected by qRT-PCR. Expression data were normalized to the expression of 18s RNA.

Data with different lowercase letter indicates significant differences (P<0.05) between each mice group.

D, The sucrose linear (10%-40%) gradients were performed to isolate 8S-CAV1 (fractions 3 to 5) and 70S-CAV1 (fractions 9 to 11) complexes in the gallbladder of mice^{WT}/chow, mice^{WT}/0.5% CA and mice$^{FXR\text{-}KO}$/0.5% CA.

chow, fed on chow diet; 0.5% CA, fed on chow diet with 0.5% CA; WD, fed on western diet. mice^{WT}/chow, chow-fed mice^{WT}; mice$^{C70\text{-}KO}$/chow, chow-fed mice$^{C70\text{-}KO}$; mice^{WT}/WD, WD-fed mice^{WT}; mice$^{C70\text{-}KO}$/WD, WD-fed mice$^{C70\text{-}KO}$; mice$^{C70\text{-}KO}$/WD/ACPD, ACPD-treated (10 mg/kg/day) WD-fed mice$^{C70\text{-}KO}$; mice$^{C70\text{-}KO}$/WD/GlyMCA, GlyMCA-treated (50 mg/kg/day) WD-fed mice$^{C70\text{-}KO}$; mice$^{C70\text{-}KO}$/WD/TβMCA, TβMCA-treated (500 mg/kg/day) WD-fed mice$^{C70\text{-}KO}$; mice^{WT}/0.5% CA, 0.5% CA-fed mice^{WT}; mice$^{FXR\text{-}KO}$/0.5% CA, 0.5% CA-fed mice$^{FXR\text{-}KO}$.

Individual P values were provided in Primary Data for Figure S13.

Figure S14

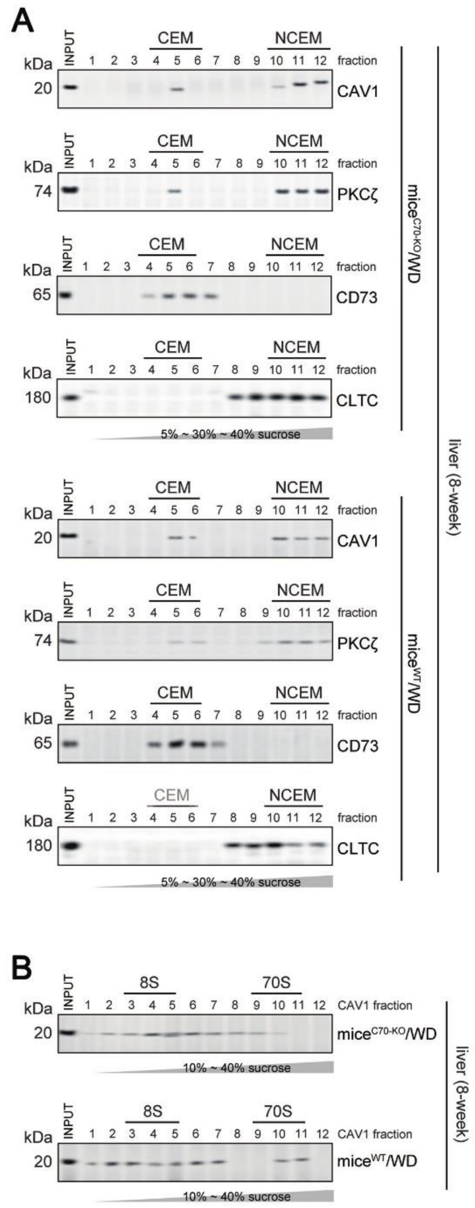
A
INPUT
CEM
NCEM
fraction
kDa
20
CAV1
74
PKCζ
65
CD73
180
CLTC
5% ~ 30% ~ 40% sucrose
mice^C70-KO/WD
mice^WT/WD
liver (8-week)
B
8S
70S
CAV1 fraction
10% ~ 40% sucrose

Figure S14. The contents of PKCζ caveolae distribution and the presence of 8S-CAV1 complexes in the liver of WD-fed mice$^{C70\text{-}KO}$ and mice^{WT}.

A, The sucrose linear (5%–30%-40%) gradients were performed to isolate CEM (fractions 4 to 6) or NCEM (fractions 10 to 12) fractions from the liver of mice$^{C70\text{-}KO}$/WD and mice^{WT}/WD. CAV1 and PKCζ are mainly localized in the NCEM of the liver of mice$^{C70\text{-}KO}$/WD and mice^{WT}/WD.

CD73 was used as CEM subcellular markers, and CLTC (clarthin heavy chain) was used as NCEM subcellular markers.

B, The sucrose linear (10%-40%) gradients were performed to isolate 8S-CAV1 (fractions 3 to 5) and 70S-CAV1 (fractions 9 to 11) complexes in the liver of mice$^{C70\text{-}KO}$/WD and mice^{WT}/WD. mice$^{C70\text{-}KO}$/WD, WD-fed mice$^{C70\text{-}KO}$; mice^{WT}/WD, WD-fed mice^{WT}.

Figure S15

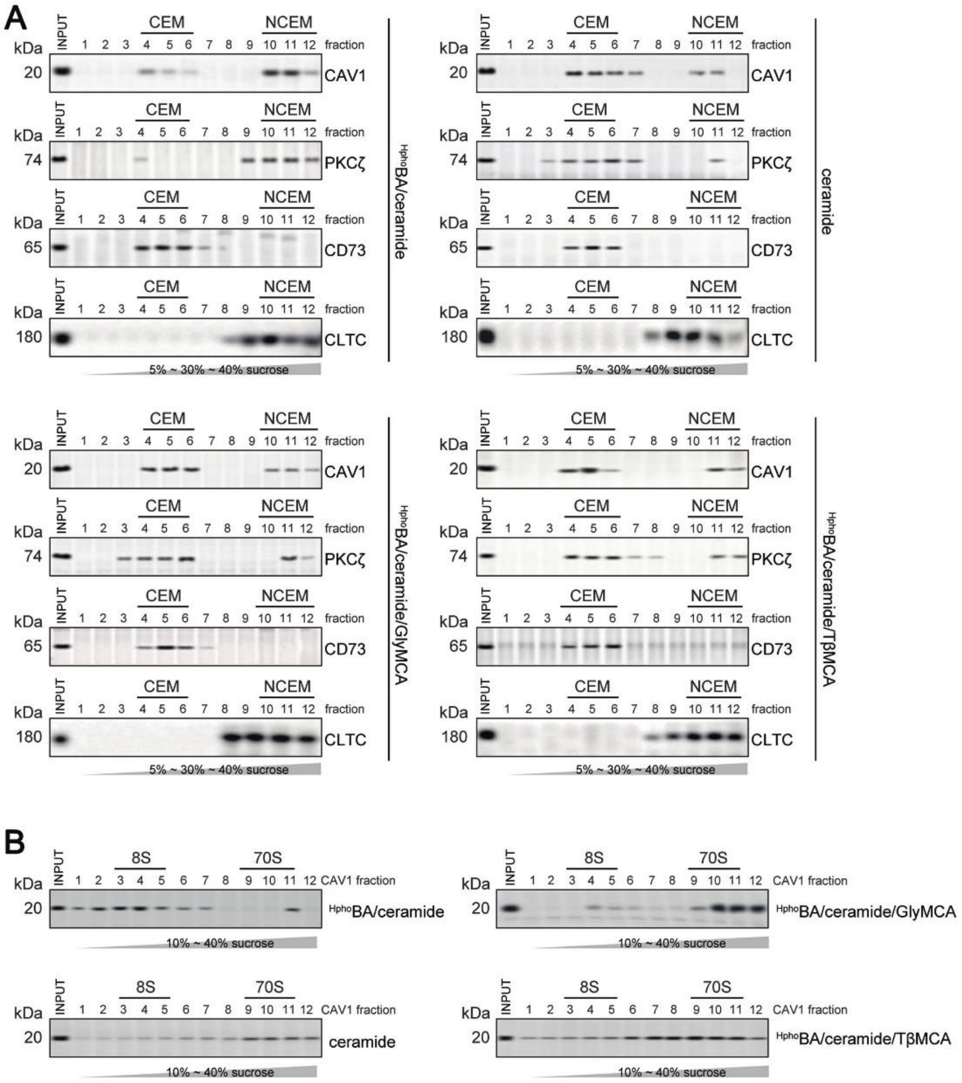
A
B
INPUT
CEM
NCEM
fraction
kDa
CAV1
PKCζ
CD73
CLTC
5% ~ 30% ~ 40% sucrose
HphoBA/ceramide
ceramide
HphoBA/ceramide/GlyMCA
HphoBA/ceramide/TβMCA
8S
70S
CAV1 fraction
10% ~ 40% sucrose

Figure S15. The reduced contents of PKCζ caveolae distribution and the instability of 70S-CAV1 complexes in in ^{Hpho}BA mixture (50 μM) and ceramide (5 μM) treated GBEC.

A, The sucrose linear (5%–30%-40%) gradients were performed to isolate CEM (fractions 4 to 6) or NCEM (fractions 10 to 12) fractions in ^{Hpho}BA mixture/ceramide treated GBEC, ceramide treated GBEC, ^{Hpho}BA mixture/GlyMCA/ceramide treated GBEC, and ^{Hpho}BA mixture/TβMCA/ceramide treated GBEC.

CAV1 and PKCζ are mainly localized in the NCEM of ^{Hpho}BA mixture/ceramide treated GBEC. CD73 was used as CEM subcellular markers, and CLTC (clarthin heavy chain) was used as NCEM subcellular markers.

B, The sucrose linear (10%-40%) gradients were performed to isolate 8S-CAV1 (fractions 3 to 5) and 70S-CAV1 (fractions 9 to 11) complexes in ^{Hpho}BA mixture/ceramide treated GBEC, ceramide treated GBEC, ^{Hpho}BA mixture/GlyMCA/ceramide treated GBEC, and ^{Hpho}BA mixture/TβMCA/ceramide treated GBEC.Note the increased amounts of 8S complexes present in ^{Hpho}BA mixture/ceramide treated GBEC.

^{Hpho}BA mixture/ceramide, ceramide (5 μM) treated GBEC with ^{Hpho}BA mixture (50 μM) pre-treatment;

ceramide, ceramide (5 μM) treated GBEC;

^{Hpho}BA mixture/GlyMCA/ceramide, ceramide (5 μM) treated GBEC with ^{Hpho}BA mixture (50 μM) and GlyMCA (50 μM) pre-treatment;

^{Hpho}BA mixture/TβMCA/ceramide, ceramide (5 μM) treated GBEC with ^{Hpho}BA mixture (50 μM) and TβMCA (200 μM) pre-treatment.

Figure S16

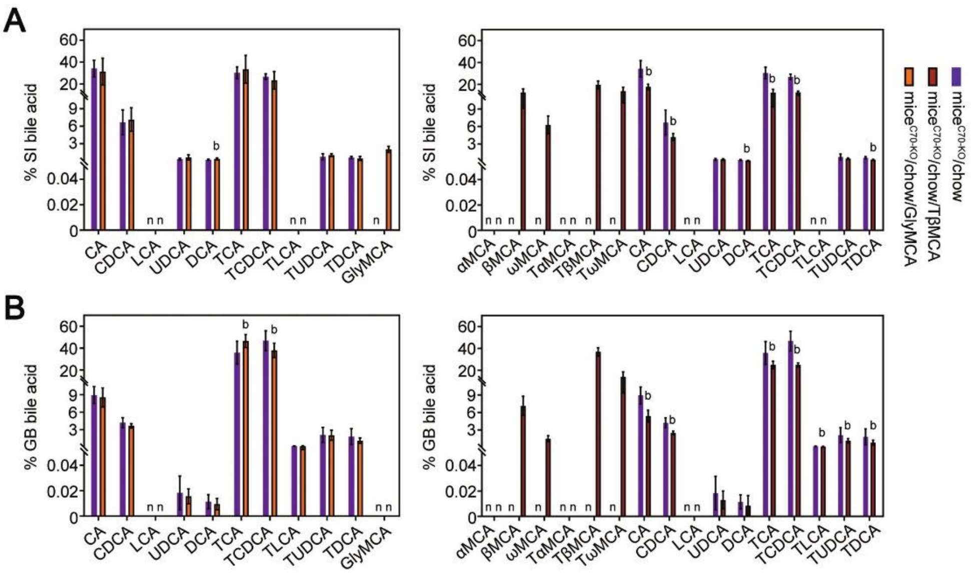
A
B
% SI bile acid
% GB bile acid
CA
CDCA
LCA
UDCA
DCA
TCA
TCDCA
TLCA
TUDCA
TDCA
GlyMCA
αMCA
βMCA
ωMCA
TαMCA
TβMCA
TωMCA
mice^C70-KO/chow
mice^C70-KO/chow/TβMCA
mice^C70-KO/chow/GlyMCA

Figure S16. Different dose of either GlyMCA or TβMCA on intestinal and gallbladder FXR signal activation and BA composition in mice$^{C70\text{-}KO}$.

chow, fed on chow diet.

GB, gallbladder; SI, small intestine.

A and B, BA composition ratio in the SI (A) and GB (B) from mice$^{C70\text{-}KO}$, with or without the treatment of either GlyMCA (50 mg/kg, left panel) or TβMCA (500 mg/kg, right panel).

GB, gallbladder; SI, small intestine.

mice$^{C70\text{-}KO}$/chow, chow-fed mice$^{C70\text{-}KO}$; mice$^{C70\text{-}KO}$/chow/GlyMCA, GlyMCA-treated (50 mg/kg/day) chow-fed mice$^{C70\text{-}KO}$; mice$^{C70\text{-}KO}$/chow/TβMCA, TβMCA-treated (500 mg/kg/day) chow-fed mice$^{C70\text{-}KO}$.

Data were presented as mean ± SD.

Data with different lowercase letter indicates significant differences (P<0.05) between each mice group.

Individual *P* values were provided in Primary Data for Figure S16.

Figure S17

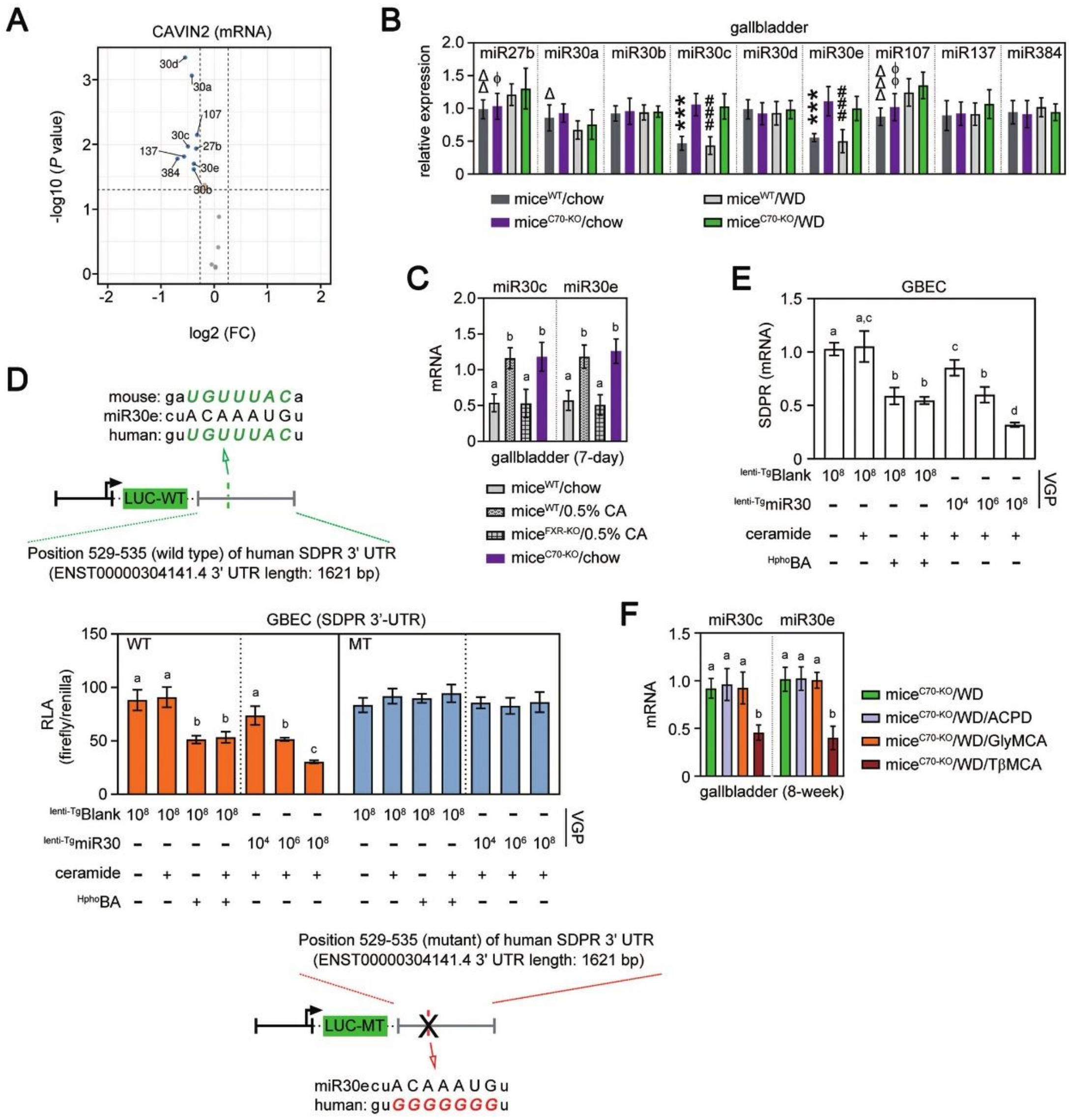
A
CAVIN2 (mRNA)
-log10 (P value)
log2 (FC)
B
gallbladder
relative expression
miR27b miR30a miR30b miR30c miR30d miR30e miR107 miR137 miR384
mice^WT/chow
mice^C70-KO/chow
mice^WT/WD
mice^C70-KO/WD
C
miR30c miR30e
mRNA
gallbladder (7-day)
mice^WT/chow
mice^WT/0.5% CA
mice^FXR-KO/0.5% CA
mice^C70-KO/chow
D
mouse: gaUGUUUACa
miR30e: cuACAAAUGu
human: guUGUUUACu
LUC-WT
Position 529-535 (wild type) of human SDPR 3' UTR
(ENST00000304141.4 3' UTR length: 1621 bp)
GBEC (SDPR 3'-UTR)
RLA (firefly/renilla)
WT
MT
lenti-TgBlank
lenti-TgmiR30
ceramide
HphoBA
VGP
Position 529-535 (mutant) of human SDPR 3' UTR
(ENST00000304141.4 3' UTR length: 1621 bp)
LUC-MT
miR30e cuACAAAUGu
human: guGGGGGGGu
E
GBEC
SDPR (mRNA)
lenti-TgBlank
lenti-TgmiR30
ceramide
HphoBA
VGP
F
miR30c miR30e
mRNA
gallbladder (8-week)
mice^C70-KO/WD
mice^C70-KO/WD/ACPD
mice^C70-KO/WD/GlyMCA
mice^C70-KO/WD/TβMCA

Figure S17. Elevated gallbladder "miR34c,e," expression down-regulated SDPR expression via the direct binding of miR30 to the 3'UTR of SDPR mRNA.

A, Volcano Plots for screening miRNA targeted SDPR gene in GBEC *in vitro*.

FC, fold change; log, logarithm.

The y axis values show the negative log 10 of the *P* value. The dotted horizontal line on the Plot represents the α-level used for this analysis (*P* = 0.05). The x axis is shown as the log 2 difference in the mean of the gene expression. Vertical dashed lines represent the threshold for the log 2 fold change (equivalent to a 1.2 fold change).

By using Targetscan and a Probability of Preferentially conserved targeting PCT score cut-off of at least 10% (httP://www.targetscan.org/cgi-bin/mmu_72/view_genetable.cgi?rs=ENSMUST00000051572.7&taxid=10090&members=&showcnc=1&shownc=1&sortText=Pct), our bioinformatics analyses revealed 17 Putative miRNA target sites for 3'UTR of SDPR mRNA among the mammalian sPecies. GBEC *in vitro* transfected with each miRNA mimics, compared with control miRNA transfected cells, were analyzed by qRT-PCR.

GBEC, gallbladder epithelial cells (HIBEpiC).

27b, mmu-miR-27b-3p; 30a, mmu-miR-30a-5p; 30b, mmu-miR-30b-5p; 30c, mmu-miR-30c-5p; 30d, mmu-miR-30d-5p; 30e, mmu-miR-30e-5p; 107, mmu-miR-107-3p; 137, mmu-miR-137-3p; 384, mmu-miR-384-5p.

B, Levels of miR27b, miR30a, miR30b, miR30c, miR30d, miR30e, miR103, miR137 and miR384 expression in the gallbldder of mice$^{C70\text{-}KO}$/chow, mice^{WT}/chow, mice$^{C70\text{-}KO}$/WD and mice^{WT}/WD were detected by qRT-PCR. Expression data were normalized to the expression of 18s RNA.

*, indicates significant differences between mice^{WT}/chow and mice$^{C70\text{-}KO}$/chow (***, *P*<0.001).

#, indicates significant differences between mice^{WT}/WD and mice$^{C70\text{-}KO}$/WD (###, *P*<0.001).

Δ, indicates significant differences between chow-fed and WD fed mice^{WT} or between chow-fed and WD fed mice$^{C70\text{-}KO}$ (Δ, *P*<0.05; ΔΔ, *P*<0.01; ΔΔΔ, *P*<0.001).

φ, indicates significant differences between chow-fed and WD fed mice^{WT} or between chow-fed and WD fed mice$^{C70\text{-}KO}$ (φ, *P*<0.05; φφ , *P*<0.01).

C, Levels of miR30c and miR30e expression in the gallbladder of chow-fed mice^{WT}, 0.5% CA (7-day) fed mice^{WT} or 0.5% CA (7-day) fed mice$^{FXR\text{-}KO}$, were detected by qRT-PCR. in the gallbldder of WD-fed mice$^{C70\text{-}KO}$ and WD-fed mice^{WT} were detected by qRT-PCR. Expression data were normalized to the expression of 18s RNA.

Data with different lowercase letter indicates significant differences (*P*<0.05) between each mice group.

D, Schematic of the full 3'UTR (1621 bp) of human SDPR gene with the wild type (WT, green) or mutated (MT, red) miR30 site was inserted into a LUC reporter as described in Supplementary Materials.

Lower panel: GBEC were transducing lentivirus carrying blank vector ($^{lenti\text{-}Tg}$Blank, 1 × 10^8 VGP) or various doses of $^{lenti\text{-}Tg}$MIR30e (from 1 × 10^4 VGP to 1 × 10^8 VGP). After 96-hour following lentiviral transduction, each group of cells were transduced with lentivirus expressing LUC reporter containing the wild type (WT) or the mutated (MT) 3'UTR of human SDPR gene. Then after 24-hour following lentiviral transduction, cells were treated with or without 50 μM ^{Hpho}BA mixture for 24-hour, and then were stimulated with 5 μM ceramide for 2-hour. RLA was normalized to β-galactosidase to correct for transfection efficiency.

VGP, viral genome particles.

10^4, 1 × 10^4 VGP; 10^6, 1 × 10^6 VGP; 10^8, 1 × 10^8 VGP.

RLA, Relative luciferase activity.

Data with different lowercase letter indicates significant differences (*P*<0.05) between each assigned GBEC group.

E, Levels of SDPR mRNA in each group of GBEC was detected by qRT-PCR. Expression data were normalized to the expression of 18s RNA.

GBEC were transducing lentivirus carrying blank vector ($^{lenti\text{-}Tg}$Blank, 1 × 10^8 VGP) or various doses of $^{lenti\text{-}Tg}$MIR30e (from 1 × 10^4 VGP to 1 × 10^8 VGP). VGP, viral genome particles.

10^4, 1 × 10^4 VGP; 10^6, 1 × 10^6 VGP; 10^8, 1 × 10^8 VGP.

Each group of cells were treated with or without 50 μM ^{Hpho}BA mixture for 24-hour, and then were stimulated with 5 μM ceramide for 2-hour.

Data with different lowercase letter indicates significant differences (*P*<0.05) between each assigned GBEC group.

F, Levels of miR30c and miR30e expression in the gallbladder of WD-fed mice$^{C70\text{-}KO}$, with or without ACPD, GlyMCA or TβMCA treatment, were detected by qRT-PCR. Expression data were normalized to the expression of 18s RNA.

Data with different lowercase letter indicates significant differences (*P*<0.05) between each mice group.

chow, fed on chow diet; 0.5% CA, fed on chow diet with 0.5% CA; WD, fed on western diet.

mice^{WT}/chow, chow-fed mice^{WT}; mice$^{C70\text{-}KO}$/chow, chow-fed mice$^{C70\text{-}KO}$; mice^{WT}/WD, WD-fed mice^{WT}; mice$^{C70\text{-}KO}$/WD, WD-fed mice$^{C70\text{-}KO}$; mice$^{C70\text{-}KO}$/WD/ACPD, ACPD-treated (10 mg/kg/day) WD-fed mice$^{C70\text{-}KO}$; mice$^{C70\text{-}KO}$/WD/GlyMCA, GlyMCA-treated (50 mg/kg/day) WD-fed mice$^{C70\text{-}KO}$; mice$^{C70\text{-}KO}$/WD/TβMCA, TβMCA-treated

(500 mg/kg/day) WD-fed mice^{C70-KO}; mice^{WT}/0.5% CA, 0.5% CA-fed mice^{WT}; mice^{FXR-KO}/0.5% CA, 0.5% CA-fed mice^{FXR-KO}.

Individual *P* values were provided in Primary Data for Figure S17.

Figure S18

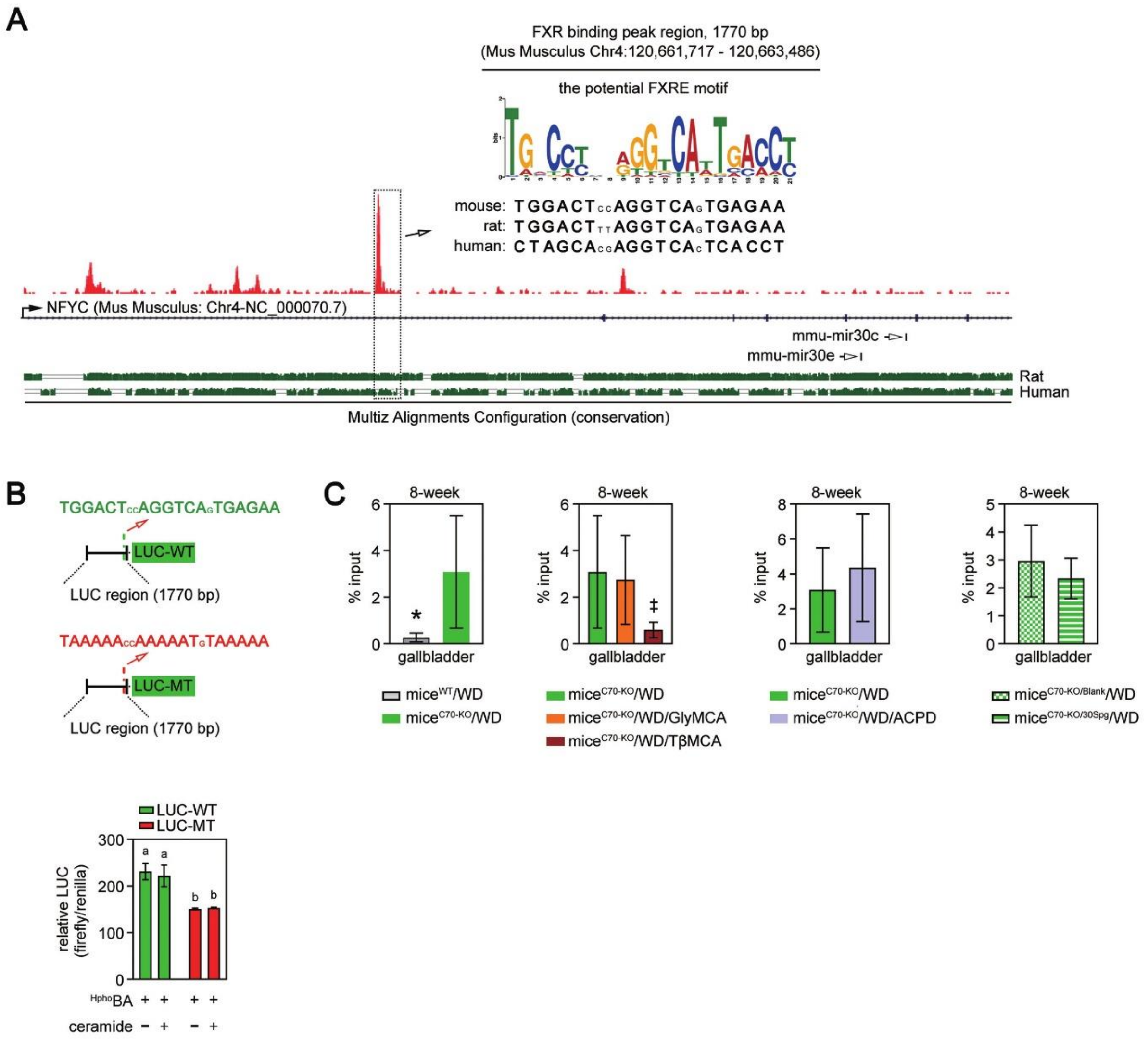
A
FXR binding peak region, 1770 bp
(Mus Musculus Chr4:120,661,717 - 120,663,486)
the potential FXRE motif
mouse: TGGACTccAGGTCAgTGAGAA
rat: TGGACTttAGGTCAgTGAGAA
human: CTAGCAcgAGGTCAcTCACCT
NFYC (Mus Musculus: Chr4-NC_000070.7)
mmu-mir30c
mmu-mir30e
Rat
Human
Multiz Alignments Configuration (conservation)
B
TGGACTccAGGTCAgTGAGAA
LUC-WT
LUC region (1770 bp)
TAAAAAccAAAAATgTAAAAA
LUC-MT
LUC region (1770 bp)
LUC-WT
LUC-MT
relative LUC (firefly/renilla)
HphoBA
ceramide
C
8-week
% input
gallbladder
miceWT/WD
miceC70-KO/WD
miceC70-KO/WD/GlyMCA
miceC70-KO/WD/TβMCA
miceC70-KO/WD/ACPD
miceC70-KO/Blank/WD
miceC70-KO/30Spg/WD

Figure S18. ***In vivo/vitro*** **FXR inactivation decreased its occupancy at the intron 1 of NFYC gene.**

RLA, Relative luciferase activity.

A, ChIP-Seq analysis of genome-wide FXR binding sites identifies an FXR resPonse element (FXRE) motif in intron 1 of the NFYC gene (httP://genome.ucsc.edu/cgi-bin/hgTracks?db=mm9&lastVirtModeTyPe=default&lastVirtModeExtraState=&virtModeTyPe=default&virtMode=0&nonVirtPosition=&Position=chr4%3A120430040%2D120498320&hgsid=997414783_9TA2FZAGrmPOxdNqbPCMLMibGl9e).

The dotted box shows the location and evolutionary conservation of FXRE among mouse, rat and human within intron 1 of the NFYC gene (arrow).

B, Schematic of the 1770 bp fragment of murine NFYC gene with the wild type (WT, green) or mutated (MT, red) FXRE motif was inserted into a LUC reporter (left panel) as described in Supplementary Materials.

The LUC-WT or LUC-MT Plasmids were respectively transfected into GBEC. After 24-hour following transfection, the cells were treated for 24-hour with 50 μM ^{Hpho}BA mixture. Then the cells were stimulated with or without 5 μM ceramide for 2-hour. RLA was normalized to β-galactosidase to correct for transfection efficiency (right panel).

Data with different lowercase letter indicates significant differences (P<0.05) between each assigned group.

C. ChIP-qPCR was performed in each mice group to examine the occupancy of FXR at the FXRE motif at NFYC gene. ChIP-qPCR analysis was performed using the percent input method.

Data with different symbol (*, mice$^{C70\text{-}KO}$ vs. mice^{WT}; ‡, mice$^{C70\text{-}KO}$/WD/TβMCA vs. mice$^{C70\text{-}KO}$/WD) indicates significant differences (P<0.05).

WD, fed on western diet.

mice^{WT}/WD, WD-fed mice^{WT}; mice$^{C70\text{-}KO}$/WD, WD-fed mice$^{C70\text{-}KO}$; mice$^{C70\text{-}KO}$/WD/GlyMCA, GlyMCA-treated (50 mg/kg/day) WD-fed mice$^{C70\text{-}KO}$; mice$^{C70\text{-}KO}$/WD/TβMCA, TβMCA-treated (500 mg/kg/day) WD-fed mice$^{C70\text{-}KO}$; mice$^{C70\text{-}KO}$/WD/ACPD, ACPD-treated (10 mg/kg/day) WD-fed mice$^{C70\text{-}KO}$; mice$^{C70\text{-}KO}$/WD/Blank, AAV-Null injected WD-fed mice$^{C70\text{-}KO}$; mice$^{C70\text{-}KO}$/WD/30Spg, AAV-30Spg injected WD-fed mice$^{C70\text{-}KO}$.

Data were presented as mean ± SD.

Individual P values were provided in Primary Data for Figure S18.

Figure S19

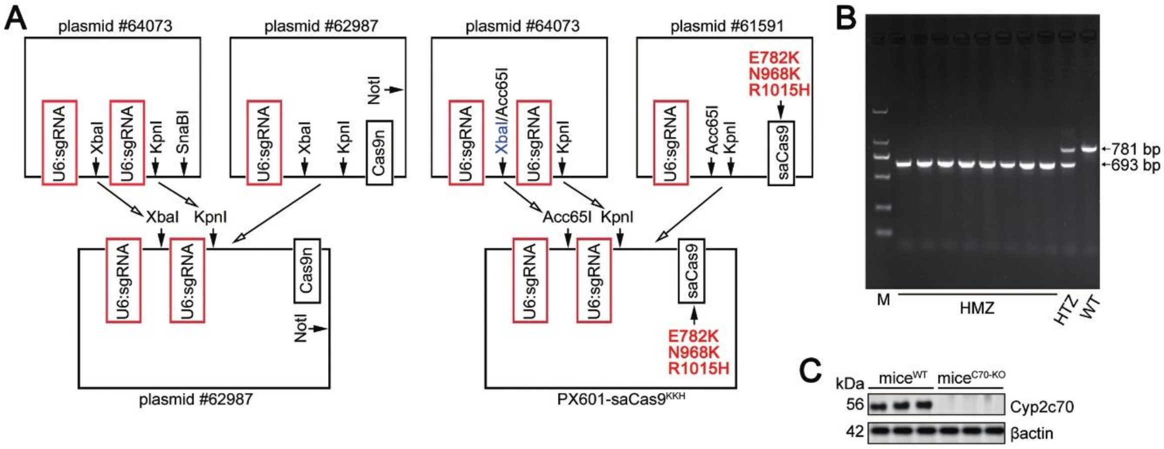
A
plasmid #64073
U6:sgRNA
XbaI
U6:sgRNA
KpnI
SnaBI
plasmid #62987
U6:sgRNA
XbaI
KpnI
Cas9n
NotI
XbaI
KpnI
U6:sgRNA
U6:sgRNA
Cas9n
NotI
plasmid #62987
plasmid #64073
U6:sgRNA
XbaI/Acc65I
U6:sgRNA
KpnI
plasmid #61591
E782K
N968K
R1015H
U6:sgRNA
Acc65I
KpnI
saCas9
Acc65I
KpnI
U6:sgRNA
U6:sgRNA
saCas9
E782K
N968K
R1015H
PX601-saCas9KKH
B
781 bp
693 bp
M
HMZ
HTZ
WT
C
kDa
miceWT
miceC70-KO
56
Cyp2c70
42
βactin

Figure S19. The model of mice$^{C70\text{-}KO}$.

A, Schematic of the sgRNA-Cas9 vector.

Left panel: the construction of U6:sgRNA/U6:sgRNA-CMV-pSpCas9n vector. Right panel: the construction of saCas9^{KKH}-U6:sgRNA/U6:sgRNA. The blue letters represent the mutation of an unwanted XbaI restriction site at 497 bp to Acc65I restriction site in the PX333 vector (plasmid #64073). The red letters represent the mutation of the SaCas9^{KKH} (E782K/N968K/R1015H) mutants in the PX601 vector (plasmid #61591).

B, PCR genotyping of Cyp2c70−/− (HMZ), Cyp2c70+/− (HTZ), and Cyp2a12+/+ (WT) in pups for the screening of F0 mice. Using genomic DNA obtained from tail clippings. WT, wild-type; HTZ, heterozygous; HMZ, homozygous. M, 2000 bp DNA Marker.

C, WB analysis of Cyp2c70 expression in liver tissue lysates. The expression of βactin was used as a loading internal control.

Figure S20

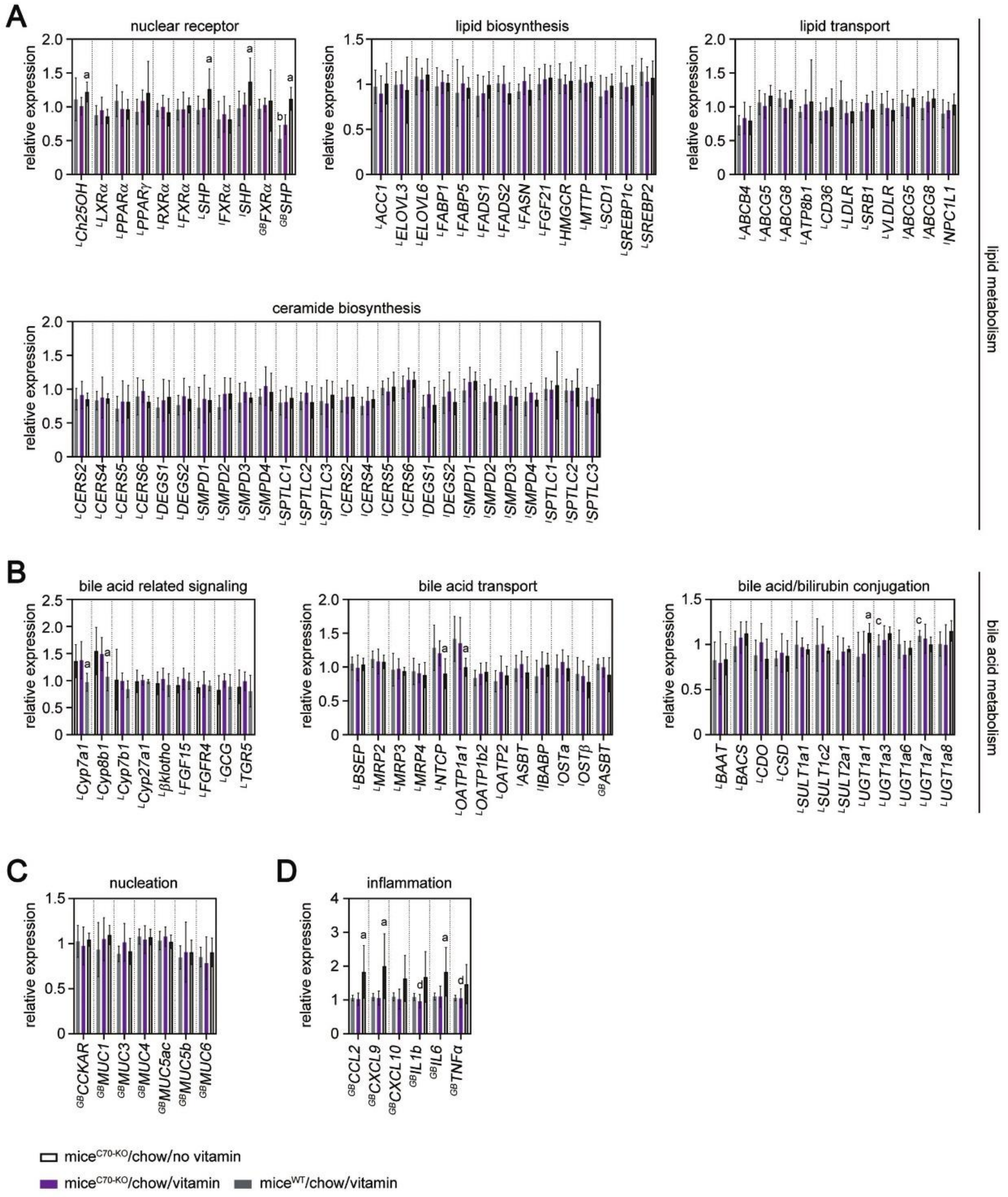
A
nuclear receptor
lipid biosynthesis
lipid transport
ceramide biosynthesis
relative expression
lipid metabolism
B
bile acid related signaling
bile acid transport
bile acid/bilirubin conjugation
bile acid metabolism
C
nucleation
D
inflammation
mice^C70-KO/chow/no vitamin
mice^C70-KO/chow/vitamin
mice^WT/chow/vitamin

Figure S20. Oral administration of vitamin mixture alters gallbladder, liver and ileum gene expression.

A, superscript "L", liver; superscript "GB", gallbladder; superscript "I", small intestine.

The mRNA levels of genes associated with lipid metabolism [nuclear receptor (cholesterol 25-hydroxylase (Ch25OH), liver X receptor α (LXRα), peroxisome proliferator-activated receptor α (PPARα), PPARγ, retinoid X receptor α (RXRα), farnesoid X receptor (FXRα), small heterodimer partner (SHP), lipid biosynthesis (Acetyl-CoA carboxylase 1 (ACC1), elongation of very long chain fatty acids protein 3 (ELOVL3), ELOVL6, fatty acid binding protein 1 (FABP1), FABP5, fatty acid desaturase 1 (FADS1), FADS2, fatty acid synthase (FASN), fibroblast growth factor 21 (FGF21), 3-Hydroxy-3-methylglutaryl-CoA reductase (HMGCR), microsomal triglyceride transfer protein (MTTP), stearoyl-CoA desaturase 1 (SCD1), sterol regulatory element-binding protein 1 (SREBP1c), SREBP2), lipid transport (ATP binding cassette subfamily B member 4 (ABCB4), ATP binding cassette subfamily G member 5 (ABCG5), ABCG8, ATPase phospholipid transporting 8b1 (ATP8b1), CD36, low density lipoprotein receptor (LDLR), scavenger receptor class B type 1 (SRB1), very low density lipoprotein receptor (VLDLR), Niemann-Pick disease type C1-like intracellular cholesterol transporter 1 (NPC1L1) and ceramide biosynthesis (ceramide synthase 2 (CERS2), CERS4, CERS5, CERS6, delta 4-desaturase sphingolipid 1 (DEGS1), DEGS2, sphingomyelin phosphodiesterase 1 (SMPD1), SMPD2, SMPD3, SMPD4, serine palmitoyltransferase long chain base subunit 1 (SPTLC1), SPTLC2, SPTLC3)] in liver and small intestine of chow-fed (8-week) mice^{WT} and mice$^{C70\text{-}KO}$, with or without oral administration of vitamin mixture. Expression data were normalized to the expression of 18s RNA.

B, superscript "L", liver; superscript "GB", gallbladder; superscript "I", small intestine.

The mRNA levels of genes associated with bile acid metabolism [bile acid related signaling (cytochrome P450 family 7 subfamily a member 1 (Cyp7a1), Cyp8b1, Cyp27a1, Cyp7b1, βklotho, fibroblast growth factor 15 (FGF15), fibroblast growth factor receptor 4 (FGFR4), proglucagon gene (GCG), takeda G protein-coupled receptor 5 (TGR5), bile acid transport (bile salt export pump (BSEP), multidrug resistance-associated protein 2 (MRP2), MRP3, MRP4, sodium taurocholate cotransporting polypeptide (NTCP), organic anion transporting polypeptide 1a1 (OATP1a1), OATP1b2, OATP2, apical sodium dependent bile acid transporter (ASBT), ileal bile acid binding protein (IBABP), organic solute transporter α (OSTα), OSTβ) and bile acid/bilirubin conjugation (bile acid-CoA:amino acid N-acyltransferase (BAAT), bile acyl-CoA synthetase (BACS), cysteine dioxygenase (CDO), cysteine sulfinate decarboxylase (CSD), sulfotransferase 1a1 (SULT1a1), SULT1c2, SULT2a1, UDP glucuronosyl-transferase

family 1 member a1 (UGT1a1), UGT1a3, UGT1a6, UGT1a7, UGT1a8] in liver, small intestine and gallbladder of chow-fed (8-week) $mice^{WT}$ and $mice^{C70\text{-}KO}$, with or without oral administration of vitamin mixture.

Expression data were normalized to the expression of 18s RNA.

C, superscript "GB", gallbladder.

The mRNA levels of genes associated with gallbladder cholesterol nucleation [cholecystokinin receptor type a (CCKAR), mucin 1 (MUC1), MUC3, MUC4, MUC5ac, MUC5b, MUC6] in gallbladder of chow-fed (8-week) $mice^{WT}$ and $mice^{C70\text{-}KO}$, with or without oral administration of vitamin mixture.

Expression data were normalized to the expression of 18s RNA.

D, superscript "GB", gallbladder.

The mRNA levels of genes associated with gallbladder inflammation [C-C motif chemokine ligand 2 (CCL2), chemokine (C-X-C motif) ligand 9 (CXCL9), CXCL10, interleukin 1b (IL1b), IL6, tumor necrosis factor α (TNFα)] in gallbladder of chow-fed (8-week) $mice^{WT}$ and $mice^{C70\text{-}KO}$, with or without oral administration of vitamin mixture.

Expression data were normalized to the expression of 18s RNA.

chow, fed on chow diet; no vitamin, drinking water contains no vitamin mixture; vitamin, drinking water contains vitamin mixture (Table S9).

$mice^{C70\text{-}KO}$/chow/no vitamin, chow-fed $mice^{C70\text{-}KO}$ on normal drinking water contains no vitamin mixture; $mice^{C70\text{-}KO}$/chow/vitamin, chow-fed $mice^{C70\text{-}KO}$ on drinking water contains vitamin mixture; $mice^{WT}$/chow/vitamin, chow-fed $mice^{WT}$ on drinking water contains vitamin mixture.

Data were presented as mean ± SD.

Data with different lowercase letter (a, $mice^{C70\text{-}KO}$/chow/no vitamin vs. $mice^{C70\text{-}KO}$/chow/vitamin and $mice^{WT}$/chow/vitamin; b, $mice^{WT}$/chow/vitamin vs. $mice^{C70\text{-}KO}$/chow/vitamin and $mice^{C70\text{-}KO}$/chow/no vitamin; c, $mice^{WT}$/chow/vitamin vs. $mice^{C70\text{-}KO}$/chow/no vitamin; d, $mice^{C70\text{-}KO}$/ chow/no vitamin vs. $mice^{C70\text{-}KO}$/chow/vitamin) indicates significantly differences ($P<0.05$).

Individual *P* values were provided in Primary Data for Figure S20.

Figure S21

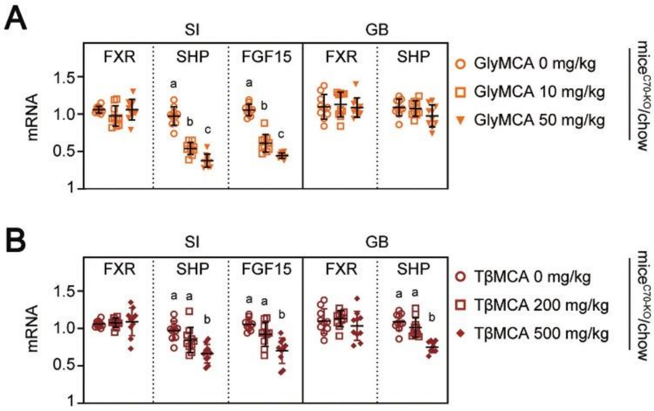
A
SI
GB
FXR
SHP
FGF15
FXR
SHP
1.5
1.0
0.5
1
mRNA
a
b
c
a
b
c
GlyMCA 0 mg/kg
GlyMCA 10 mg/kg
GlyMCA 50 mg/kg
mice^C70-KO/chow
B
SI
GB
FXR
SHP
FGF15
FXR
SHP
1.5
1.0
0.5
1
mRNA
a a b
a a b
a a b
TβMCA 0 mg/kg
TβMCA 200 mg/kg
TβMCA 500 mg/kg
mice^C70-KO/chow

Figure S21. Different dose of either GlyMCA or TβMCA on intestinal and gallbladder FXR signal activation in mice$^{C70\text{-}KO}$.

chow, fed on chow diet.

GB, gallbladder; SI, small intestine.

A and B, The mRNA levels of FXR, SHP and FGF15 were measured by qRT-PCR in SI and GB of mice$^{C70\text{-}KO}$ after 7-day treatment with either various doses (0 mg/kg, 10 mg/kg, 50 mg/kg) of GlyMCA (A) or various doses (0 mg/kg, 200 mg/kg, 500 mg/kg) TβMCA (B). Expression data were normalized to the expression of 18s RNA.

Data were presented as mean ± SD.

Data with different lowercase letter indicates significant differences (P<0.05) between each mice group.

Individual *P* values were provided in Primary Data for Figure S21.

Table S1A. Biliary lipid composition of mice^{WT} and mice$^{C70\text{-}KO}$.

Biliary lipid	mice^{WT}			mice$^{C70\text{-}KO}$	
BA (mM)	106.2 (3)	174.9 (18.1)a,¶,***	219.4 (17.6)a	107.9 (2.5)	157.3 (6.2)a,***
PL (mM)	20.3 (2.9)	35.9 (1.9)a	40.9 (3.5)a	21.1 (1.6)	38.6 (2.8)a
Ch (mM)	4.4 (0.7)	20.2 (2.2)a,¶¶¶,***	16.3 (1.0)a	4.8 (0.8)	16.8 (1.3)a*
TL (g/dL)	7.2 (0.4)	12.5 (1)a,¶,***	15.0 (1.0)a	7.3 (0.2)	11.6 (0.4)a,***
CSI (%)	66.5 (10)	145.7 (15.8)a,¶¶,***	100.7 (10.5)a	70.9 (12.9)	122.8 (6.7)a,***
week	0	8	8	0	8
diet	WD	LD	WD		

Data exhibited as mean (S.D.). Individual *P* values were provided in Primary data for Supplementary Table 1A.

BA, bile acid; PL, phospholipid; Ch, cholesterol; TL, total lipid; CSI, cholesterol saturation index.

a, significant differences between 0-week vs. 8-week in same group.

Data with different symbol (*, WD-fed mice^{WT} vs. LD-fed mice^{WT} or vs. WD-fed mice$^{C70\text{-}KO}$; ¶, LD-fed mice^{WT} vs. WD-fed mice$^{C70\text{-}KO}$) indicates significant differences between each mice group (**, $P < 0.01$; ***, $P < 0.001$; ¶, $P < 0.05$; ¶¶, $P < 0.01$).

Table S1B. Biliary lipid composition of mice$^{C70\text{-}KO}$, with i.p. injection of AAV2/8-Cas9-based sgCTRL or sgMUC1.

Biliary lipid	Treatment	
	sgCTRL	sgMUC1
BA (mM)	176.6 (12.1)	172.5 (13.2)
PL (mM)	41.8 (3)	42.3 (2.8)
Ch (mM)	18.5 (1.6)	19.6 (1.8)
TL (g/dL)	12.8 (0.5)	12.7 (0.7)
CSI (%)	120.5 (5.1)	126.2 (11.3)
week	8	
diet	WD	

Data exhibited as mean (S.D.).

i.p. injection, intraperitoneal injection.

BA, bile acid; PL, phospholipid; Ch, cholesterol; TL, total lipid; CSI, cholesterol saturation index.

Table S1C. Biliary lipid composition of mice$^{C70\text{-}KO}$, with i.p. injection of AAV2/8-Cas9-based sgCTRL or sgMUC4.

Biliary lipid	Treatment	
	sgCTRL	sgMUC4
BA (mM)	176.6 (12.1)	172.1 (8.6)
PL (mM)	41.8 (3)	43.2 (3.3)
Ch (mM)	18.5 (1.6)	19.1 (1.7)
TL (g/dL)	12.8 (0.5)	12.8 (0.4)
CSI (%)	120.5 (5.1)	121.2 (14.1)
week	8	
diet	WD	

Data exhibited as mean (S.D.).

i.p. injection, intraperitoneal injection.

BA, bile acid; PL, phospholipid; Ch, cholesterol; TL, total lipid; CSI, cholesterol saturation index.

Table S1D. Biliary lipid composition of mice$^{C70\text{-}KO}$, with i.p. injection of AAV2/8-Cas9-based sgCTRL or sgMUC5ac.

Biliary lipid	Treatment	
	sgCTRL	sgMUC5ac
BA (mM)	176.6 (12.1)	164 (24)
PL (mM)	41.8 (3)	43.5 (4.8)
Ch (mM)	18.5 (1.6)	20.1 (1.7)
TL (g/dL)	12.8 (0.5)	12.4 (1.5)
CSI (%)	120.5 (5.1)	129.9 (18.2)
week	8	
diet	WD	

Data exhibited as mean (S.D.).

i.p. injection, intraperitoneal injection.

BA, bile acid; PL, phospholipid; Ch, cholesterol; TL, total lipid; CSI, cholesterol saturation index.

Table S1E. Biliary lipid composition of WD-fed mice^{WT} (8-week), with i.p. injection of AAV2/8-based null vector or MUC1.

Biliary lipid	Treatment	
	Blank	MUC1-OE
BA (mM)	225.7 (21.3)	222 (33)
PL (mM)	43.4 (5.4)	45.2 (5.7)
Ch (mM)	16.8 (2.1)	17 (2.8)
TL (g/dL)	15.6 (1.3)	15.5 (1.6)
CSI (%)	97.9 (13.8)	96.7 (10.8)
week	8	
diet	WD	

Data exhibited as mean (S.D.).

i.p. injection, intraperitoneal injection.

Blank, AAV2/8-based null vector; MUC1-OE, AAV2/8-based MUC1 over-expression.

BA, bile acid; PL, phospholipid; Ch, cholesterol; TL, total lipid; CSI, cholesterol saturation index.

Table S1F. Biliary lipid composition of LD-fed mice^{WT} (3-week), with i.p. injection of AAV2/8-based null vector or MUC1.

Biliary lipid	Treatment	
	Blank	MUC1-OE
BA (mM)	168 (16.7)	165.9 (23.8)
PL (mM)	42.2 (1.9)	40.7 (3.6)
Ch (mM)	20.5 (1.7)	19.3 (1.4)
TL (g/dL)	12.7 (1)	12.4 (1.1)
CSI (%)	133.3 (9.1)	130 (8.2)
week	3	
diet	LD	

Data exhibited as mean (S.D.). Individual *P* values were provided in Primary data for Supplementary Table 1F.

i.p. injection, intraperitoneal injection.

Blank, AAV2/8-based null vector; MUC1-OE, AAV2/8-based MUC1 over-expression.

BA, bile acid; PL, phospholipid; Ch, cholesterol; TL, total lipid; CSI, cholesterol saturation index.

, indicates significant differences between between Blank vs. MUC1-OE after 8-week WD feeding (, $P < 0.05$).

Table S1G. Biliary lipid composition of mice$^{C70\text{-}KO}$, under the treatment of either VEH or GlyMCA or TβMCA.

Biliary lipid	Treatment		
	no treatment	GlyMCA	TβMCA
BA (mM)	157.3 (6.2)a	199.1 (30.6)a,##	179.5 (9.7)a,###
PL (mM)	38.6 (2.8)a	43.4 (3.8)a,#	37.5 (2.6)a,ΔΔ
Ch (mM)	16.8 (1.3)a	13.8 (1)a,###	17.4 (1.5)a,ΔΔΔ
TL (g/dL)	11.6 (0.4)a	13.9 (1.5)a,##	12.7 (0.5)a,###
CSI (%)	122.8 (6.7)a	85.8 (8.7)a,###	123 (11.6)a,ΔΔΔ
week	8		
diet	WD		

Data exhibited as mean (S.D.). Individual *P* values were provided in Primary data for Supplementary Table 1G.

BA, bile acid; PL, phospholipid; Ch, cholesterol; TL, total lipid; CSI, cholesterol saturation index.

a, significant differences between 0-week vs. 8-week in same group.

Data with different symbol (#, no treatment vs. GlyMCA or TβMCA treatment; Δ, TβMCA vs. GlyMCA) indicates significant differences between each mice group at the same time point (#, $P < 0.05$; ##, $P < 0.01$; ###, $P < 0.001$; ΔΔ, $P < 0.01$; ΔΔΔ, $P < 0.001$).

Table S1H. Biliary lipid composition of mice$^{C70\text{-}KO}$, under the treatment of VEH or ACPD.

Biliary lipid	Treatment	
	no treatment	ACPD
BA (mM)	157.3 (6.2)a	170.2 (11.1)a,##
PL (mM)	38.6 (2.8)a	43.3 (4.9)a,#
Ch (mM)	16.8 (1.3)a	14.6 (2.5)a,#
TL (g/dL)	11.6 (0.4)a	12.6 (0.6)a,###
CSI (%)	122.8 (6.7)a	96 (18.9)a,##
week	8	
diet	WD	

Data exhibited as mean (S.D.). Individual *P* values were provided in Primary data for Supplementary Table 1H.

BA, bile acid; PL, phospholipid; Ch, cholesterol; TL, total lipid; CSI, cholesterol saturation index.

a, significant differences between 0-week vs. 8-week in same group.

#, indicates significant differences between between each mice group (no treatment vs. ACPD) at the same time point (#, $P < 0.05$; ##, $P < 0.01$).

Table S1I. Biliary lipid composition of mice$^{C70\text{-}KO}$, with i.p. injection of Blank or 30Spg.

Biliary lipid	Treatment	
	Blank	30Spg
BA (mM)	167.6 (31.2)	178.6 (16.9)
PL (mM)	41.9 (4.1)	40 (3.7)
Ch (mM)	20 (3.2)	19.8 (2.4)
TL (g/dL)	12.5 (1.6)	12.9 (1)
CSI (%)	131.8 (19.4)	132.4 (12.7)
week	8	
diet	WD	

Data exhibited as mean (S.D.). Individual *P* values were provided in Primary data for Supplementary Table 1I.

i.p. injection, intraperitoneal injection.

BA, bile acid; PL, phospholipid; Ch, cholesterol; TL, total lipid; CSI, cholesterol saturation index.

Blank, AAV-Null virus; 30Spg, AAV-30Spg virus.

Table S2A. Hepatic and serum parameters of mice^{WT} and mice$^{C70\text{-}KO}$.

hepatic&serum parameters	mice^{WT}		mice$^{C70\text{-}KO}$	
Hepatic FC (mg/g)	2.4 (0.4)a	21.8 (6.1)	2.3 (0.2)a	23.2 (6.4)
Hepatic CE (mg/g)	1.3 (0.2)a	13.1 (4.6)	1.3 (0.1)a	13.5 (6.2)
Hepatic TG (mg/g)	49.2 (3.2)a	132.5 (24.9)	50 (2.9)a	130.2 (30.5)
Hepatic FFA (μEq/liter)	37.3 (3.1)a	83.7 (15.5)	39.5 (5.8)a	86.4 (27.5)
Serum TC (mg/dL)	122 (10.3)a	254.1 (66.4)	123.5 (7.9)a	253.4 (62.1)
Serum LDL-C (mg/dL)	28.1 (2.2)a	89.7 (19.6)	27.5 (3.1)a	95.2 (16.3)
Serum TG (mg/dL)	69.8 (16.2)a	148.8 (37.2)	83.6 (16.5)a	144.4 (32.1)
Serum FFA (μEq/liter)	580.7 (31.6)a	780.4 (30.4)	602.3 (23.8)a	784.8 (56.6)
FBG (mg/dL)	84.2 (9.5)a	145.1 (19.9)	78.3 (12.4)a	147.1 (22.2)
Serum ALT (U/L)	71.9 (37.7)a***	151.4 (16.4)*	155 (31.6)	188.5 (40.1)
Serum AST (U/L)	173.6 (41.2)a***	301.3 (48.4)**	285.3 (38)a	412.8 (98.2)
week	0	8	0	8
diet	WD			

Data exhibited as mean (S.D.). Individual *P* values were provided in Primary data for Supplementary Table 2A.

FC, free cholesterol; CE, cholesterol ester; TG, triglyceride; FFA, free fatty acid; TC, total cholesterol in the blood; LDL-C, low-density lipoprotein carry cholesterol in the blood; FBG, fasting blood glucose; ALT, Alanine Aminotransferase; AST, Aspartate Aminotransferase.

a, significant differences between 0-week vs. 8-week.

, indicates significant differences between between each mice group (mice^{WT} vs. mice$^{C70\text{-}KO}$) at the same time point (, $P < 0.05$; **, $P < 0.01$; ***, $P < 0.001$).

Table S2B. Hepatic and serum parameters of mice$^{C70\text{-}KO}$, under the treatment of either VEH or GlyMCA or TβMCA.

hepatic&serum parameters	treatment		
	no treatment	GlyMCA	TβMCA
Hepatic FC (mg/g)	23.2 (6.4)a	14.3 (1.7)a,###	23.2 (7)a,ΔΔΔ
Hepatic CE (mg/g)	13.5 (6.2)a	9.4 (2.1)a	9.3 (3)a
Hepatic TG (mg/g)	130.2 (30.5)a	61.8 (7)a,###	97.7 (19)a,ΔΔΔ,#
Hepatic FFA (μEq/liter)	86.4 (27.5)a	59.7 (14)a	120.3 (63.1)a,Δ
Serum TC (mg/dL)	253.4 (62.1)a	225.7 (18.5)a,#	281.7 (73.3)a,Δ
Serum LDL-C (mg/dL)	95.2 (16.3)a	46.4 (4.8)a,###	120.9 (27.8)a,ΔΔΔ
Serum TG (mg/dL)	144.4 (32.1)a	97.7 (6.3)a,##	94.2 (13.4)##
Serum FFA (μEq/liter)	784.8 (56.6)a	574.1 (56.7)###	617.8 (86.3)###
FBG (mg/dL)	147.1 (22.2)a	90.4 (12.1)###	105.4 (8)a##
Serum ALT (U/L)	188.5 (40.1)	172 (76.2)	225.1 (87.4)a
Serum AST (U/L)	412.8 (98.2)a	287.8 (62.2)#	474.3 (87.5)a,ΔΔΔ
week	8		
diet	WD		

Data exhibited as mean (S.D.). Individual *P* values were provided in Primary data for Supplementary Table 2B.

FC, free cholesterol; CE, cholesterol ester; TG, triglyceride; FFA, free fatty acid; TC, total cholesterol in the blood; LDL-C, low-density lipoprotein carry cholesterol in the blood; FBG, fasting blood glucose; ALT, Alanine Aminotransferase; AST, Aspartate Aminotransferase.

a, significant differences between 0-week vs. 8-week.

Data with different symbol (#, GlyMCA or TβMCA treatment vs. no treatment; Δ, TβMCA vs. GlyMCA) indicates significant differences between each mice group at the same time point. #, $P < 0.05$; ##, $P < 0.01$; ###, $P < 0.001$; Δ, $P < 0.05$; ΔΔΔ, $P < 0.001$.

Table S2C. Hepatic and serum parameters of mice$^{C70\text{-}KO}$, under the treatment of VEH or ACPD.

hepatic&serum parameters	treatment	
	no treatment	ACPD
Hepatic FC (mg/g)	23.2 (6.4)a	13.5 (1.9)a,##
Hepatic CE (mg/g)	13.5 (6.2)a	8.7 (2.6)a
Hepatic TG (mg/g)	130.2 (30.5)a	65.9 (4.7)a,###
Hepatic FFA (μEq/liter)	86.4 (27.5)a	56.2 (16.2)a,#
Serum TC (mg/dL)	253.4 (62.1)a	207.5 (19.8)a,##
Serum LDL-C (mg/dL)	95.2 (16.3)a	44.2 (6.7)a,###
Serum TG (mg/dL)	144.4 (32.1)a	94.2 (6.4)##
Serum FFA (μEq/liter)	784.8 (56.6)a	567.1 (29)a,###
FBG (mg/dL)	147.1 (22.2)a	91.4 (8)a,###
Serum ALT (U/L)	188.5 (40.1)	155.4 (46.6)
Serum AST (U/L)	412.8 (98.2)a	301.7 (88.9)#
week	8	
diet	WD	

Data exhibited as mean (S.D.). Individual *P* values were provided in Primary data for Supplementary Table 2C.

FC, free cholesterol; CE, cholesterol ester; TG, triglyceride; FFA, free fatty acid; TC, total cholesterol in the blood; LDL-C, low-density lipoprotein carry cholesterol in the blood; FBG, fasting blood glucose; ALT, Alanine Aminotransferase; AST, Aspartate Aminotransferase.

a, significant differences between 0-week vs. 8-week.

#, indicates significant differences between between each mice group (no treatment vs. ACPD treatment) at the same time point (#, $P < 0.05$; ##, $P < 0.01$; ###, $P < 0.001$).

Table S2D. Hepatic and serum parameters of mice$^{C70\text{-}KO}$, with i.p. injection of Blank or 30Spg.

hepatic&serum parameters	treatment	
	Blank	30Spg
Hepatic FC (mg/g)	21.9 (6.3)	22.2 (5.3)
Hepatic CE (mg/g)	12.7 (4.9)	10.8 (3.3)
Hepatic TG (mg/g)	134 (14)	133.7 (19.2)
Hepatic FFA (μEq/liter)	92.8 (15.8)	88.9 (23.6)
Serum TC (mg/dL)	237.1 (76.2)	258.5 (69.9)
Serum LDL-C (mg/dL)	93.4 (17.2)	96 (27.4)
Serum TG (mg/dL)	137.4 (30.7)	166.1 (40)
Serum FFA (μEq/liter)	781.5 (26.8)	771.3 (39.3)
FBG (mg/dL)	142.5 (18.3)	146.4 (17.8)
Serum ALT (U/L)	212.7 (14.9)	201.2 (21.4)
Serum AST (U/L)	435.7 (91)	426 (68.1)
week	8	
diet	WD	

Data exhibited as mean (S.D.).

i.p. injection, intraperitoneal injection.

FC, free cholesterol; CE, cholesterol ester; TG, triglyceride; FFA, free fatty acid; TC, total cholesterol in the blood; LDL-C, low-density lipoprotein carry cholesterol in the blood; FBG, fasting blood glucose; ALT, Alanine Aminotransferase; AST, Aspartate Aminotransferase.

Blank, AAV-Null virus; 30Spg, AAV-30Spg virus.

Table S3. The composition of vitamin mixture in the drinking water*.

	Individual vitamin supplements in the drinking water
vitamin A	300 IU/L
vitamin B1	180 μg/L
vitamin B2	300 μg/L
vitamin B3	1500 μg/L
vitamin B5	600 μg/L
vitamin B6	150 μg/L
vitamin B9	100 μg/L
vitamin B12	150 μg/L
Vitamin C	9.0 mg/L
vitamin D3	50 IU/L
vitamin E	0.75 IU/L
menadione	250 μg/L

*: The vitamin-laced water was replaced every day.

Table S4. Primers, Antibodies, Drugs and miR30 sponge sequence.

Primers used for qRT-PCR

gene symbol (mice)	forward primer (5' → 3')	reverse primer (5' → 3')
18s rRNA	GATGGGAAGTACAGCCAGGT	TTTCTTCAGCCTCTCCAGGT
βklotho	CAGTACGTGAGTCCGGTAAAC	TCCACTTGAAATGCTCCGGTC
ABCB4	ATACTGGGAACTTTTCCTTGCC	ACCACCTAGTCCCGAATAGTAGT
ABCG5	AGAGTCAGGATGGCCTGTAT	ATGCTGAGCAGGGCCACTAT
ABCG8	GAGAGCTTCACAGCCCACAA	GCCTGAAGATGTCAGAGCGA
ACC1	ACCCACTCCACTGTTTGTGA	CCTTGGAATTCAGGAGAGGA
ASBT	TGGGTTTCTTCCTGGCTAGACT	TGTTCTGCATTCCAGTTTCCAA
ATP8b1	GTCTCAGCCCAACGATGAAGT	TCTGTTCTGGTTCAACCGTGG
BAAT	GGAAACCTGTTAGTTCTCAGGC	GTGGACCCCCATATAGTCTCC
BACS	ACCCTGGATCAGCTCCTGGAT	GTTCTCAGCTAGCAGCTTGG
BSEP	GTGTCTACTTCATGCTTGTGAC	GAGACTTAGATCGTTGACGGAT
CCKAR	CTGGAGAGATACGGTGCCATC	TGTAAATGGGGTACGGAGTCA
CCL2	AACTCTCACTGAAGCCAGCTCT	CGTTAACTGCATCTGGCTGA
CD36	TTCCAGCCAATGCCTTTGC	TGGAGATTACTTTTTCAGTGCAG
CDO	GGGGACGAAGTCAACGTGG	ACCCCAGCACAGAATCATCAG
CERS2	AAGTGGGAAACGGAGTAGCG	ACAGGCAGCCATAGTCGTTC
CERS4	GGATTAGCTGATCTCCGCAC	CCAGTATGTCTCCTGCCACA
CERS5	CTTCTCCGTGAGGATGCTGT	GTGTCATTGGGTTCCACCTT
CERS6	AAGCCAATGGACCACAAACT	TGCTTGGAGAGCCCTTCTAAT
Ch25OH	TGCTACAACGGTTCGGAGC	AGAAGCCCACGTAAGTGATGAT
CSD	CCAGGACGTGTTTGGGATTGT	ACCAGTCTTGACACTGTAGTGA
CXCL10	CCAGTGAGAATGAGGGCCATA	CTCAACACGTGGGCAGGAT
CXCL9	TGAAGTCCGCTGTTCTTTTCC	GGGTTCCTCGAACTCCACACT
Cyp27b1	GCCTCACCTATGGGATCTTCA	TCAAAGCCTGACGCAGATG
Cyp7a1	TACAGAGTGCTGGCCAAGAG	TTCAAGGATGCACTGGAGAG
Cyp7b1	AATTGGACAGCTTGGTCTGCCT	TGTGTATGAGTGGAGGAAAGAGGG
Cyp8b1	GGCTGGCTTCCTGAGCTTATT	ACTTCCTGAACAGCTCATCGG
DEGS1	AATGGGTCTACACGGACCAG	TGGTCAGGTTTCATCAAGGAC
DEGS2	AAGCCAATGGACCACAAACT	TGCTTGGAGAGCCCTTCTAAT
ELOVL3	GCCTCTCATCCTCTGGTCCT	TGCCATAAACTTCCACATCCT
ELOVL6	AGCAGAGGCGCAGAGAACACGTA	ATAAAGGCAGCGTACAGCGCAGAA
FABP1	CCATGAACTTCTCCGGCAAGTACC	CTTTGGGTCCATAGGTGATGGTGAG
FABP5	GGAAGGAGAGCACGATAACAAGA	GGTGGCATTGTTCATGACACA
FADS1	ACCCACCAAGAATAAAGCGCTAA	CAGCCACATCCAGCAGCAG
FADS2	ACCGTGGCAAAAGCTCTCAG	GAGAGGATGAACCAGGCAAGGC
FASN	TTGCTGGCACTACAGAATGC	AACAGCCTCAGAGCGACAAT
FGF15	GGCAAGATATACGGGCTGAT	GATGGTGCTTCATGGATCTG
FGF21	CTGGGGGTCTACCAAGCATA	CACCCAGGATTTGAATGACC
FGFR4	TCCATGACCGTCGTACACAAT	ATTTGACAGTATTCCCGGCAG
FXRα	TCCACAACCAAGTTTTGCAG	TCTCTGTTTGTTGTACGAATCCA
GCG	CCAGTGATGTGAGTTCTTACTTGG	CAATGG CGACTTCTTCTGG
HMGCR	CCGGCAACAACAAGATCTGTG	ATGTACAGGATGGCGATGCA
IBABP	ACCATTGGCAAAGAATGTGA	GACCTCCGAAGTCTGGTGAT
IL1b	CAACCAACAAGTG ATATTCTCCATG	GATCCACACTCTC CAGCTGCA
IL6	CCGGAGAGGAGACTTCACAGA	AGAATTGCCATTGCACAACTCTT
LDLR	CCACAGAACTGCCAGGGCCG	GAATTCATCAGGTCGGCAGGT

LXRα	GGGAGGAGTGTGTGCTGTCAG	GAGCGCCTGTTACACTGTTGC
MRP2	GGATGGTGACTGTGGGCTGAT	GGCTGTTCTCCCTTCTCATGG
MRP3	CAGCTGCTCAGTATACTCATCA	CCTATAATAGCAGTCCGTAGCC
MRP4	CAAGGGTTCCGGCTACACATT	CTACCGTAGTAAGCCCCTCTT
MTTP	ACAAGCTCACGTACTCCACTG	TCCTCCATAGTAAGGCCACATC
MUC1	ACGTGAAGTCACAGCTTATACA	AGGGCAAGGAAATAGACGATAG
MUC3	GCCGTGAATTGTATGAACGGA	CGCAGTTGACCACGTTGACTA
MUC4	CCTTCAGGGAAATACACCTGTT	TAGAGCATCTTCAGGTAGCAAG
MUC5ac	ATCTGTAAGGAAGCCACGCTAA	CTCCTCGTATGTGACTATCAGG
MUC5b	CTACTCGAACTGCTTGTTTGAC	CTTGTAGCAAACTTTGTCCCTC
MUC6	CCTTCTACAAGAGATGCGTGTA	GATCACAGGCCTGGCTATCATA
NPC1L1	GAGGCCATCTGCTTCTTTCTAG	GTCAAAGATGATTGCTAAGCCA
NTCP	ATGACCACCTGCTCCAGCTT	GCCTTTGTAGGGCACCTTGT
OATP1a1	TGGATTTATCACTGGGAGCTTT	AGGAAATGAGGTGATGCCATTA
OATP1b2	CAGTGATTGCAGACGTTCCC	AAAATAGCTTGAATCCATCGCAGA
OATP2b1	TCACCCCTCTACCTGGGAAT	ATGAGGCTACCCAGCCCATA
OSTα	TGTTCCAGGTGCTTGTCATCC	CCACTGTTAGCCAAGATGGAGAA
OSTβ	GATGCGGCTCCTTGGAATTA	GGAGGAACATGCTTGTCATGAC
PPARα	CTGGCATTTGTTCCGGTTCT	TATTCGGCTGAAGCTGGTGT
PPARγ	AGGCCGAGAAGGAGAAGCTGTTG	TGGCCACCTCTTTGCTCTGCTC
RXRα	ATGGACACCAAACATTTCCTGC	GGGAGCTGATGACCGAGAAAG
SCD1	TGTCTCGGTGTGTGTCGGAGT	TGTACCACTACCTGCCTGCATG
SHP	CAAGGAGTATGCGTACCTGAAG	GGCTCCAAGACTTCACACAGT
SMPD1	GTTACCAGCTGATGCCCTTC	AGCAGGATCTGTGGAGTTG
SMPD2	AGCAGGATCTGTGGAGTTG	CTCCAGCCATGAAGCTCAAC
SMPD3	CCTGACCAGTGCCATTCTTT	AGAAACCCGGTCCTCGTACT
SMPD4	ACCTGGCCCTCAATCCATTTG	ATAGGCACAGTCCGAAGTACG
SPTLC1	CGAGGGTTCTATGGCACATT	GGTGGAGAAGCCATACGAGT
SPTLC2	TCACCTCCATGAAGTGCATC	CAGGCGTCTCCTGAAATACC
SPTLC3	ACACAATCCTAAGACCCAGCA	AGACTGGCTTATCCTCAGCATA
SRB1	AACATCACCTTCAATGACAACG	ACCAAGATGTTAGGCAGTACAA
SREBP1c	GGAGCCATGGATTGCACATT	GCTTCCAGAGAGGAGGCCAG
SREBP2	GCGTTCTGGAGACCATGGA	ACAAAGTTGCTCTGAAAACAAATCA
SULT1a1	AACATGGAGCCCTTGCGTAAA	ATGAGCACATCATCAGGCCAG
SULT1c2	CAGCCTGCAACTGTGGACAA	GATGGCGGTGTTGGATGATG
SULT2a1	GAAGATCTGTGACTTCCTAGGG	AGCTTCAAGCCATTAGTAACCT
TGR5	CTGTGTGAGATCCGCCGAC	CGACGCTCATAGGCCAAGA
TNFα	CCACCACGCTCTTCTGTCTAC	AGGGTCTGGGCCATAGAACT
UGT1a1	GCTGTTAGTGTTCCCTATGGAT	GGGAACTTCCTCAGAGTGTAAA
UGT1a3	TGTTGAACAATATGTCTTTGGTCTA	ACCACATCAAAGGAAGTAGCA
UGT1a6	GTTTCTCTTCCTAGTGCTTTGGG	CCTCGTTCACTGAGATGTTCTAC
UGT1a7	GTTTACGATGCAGACGGTTGT	CCTCCTGCGTATGAGAAACTG
UGT1a8	CAAGCAATGGGAAAATCGAGAA	CCAGAAACACAGCATCAAAAGA
VLDLR	GAGCCCCTGAAGGAATGCC	CCTATAACTAGGTCTTTGCAGATATGG

Mature miRNA

Mature miRNA ID	company	miScript Primer Assay Catalog #
mmu-miR-27b-3p	Qiagen	MS00001358
mmu-miR-30a-5p		MS00011704
mmu-miR-30b-5p		MS00001386
mmu-miR-30c-5p		MS00011725
mmu-miR-30d-5p		MS00011746
mmu-miR-30e-5p		MS00011753
mmu-miR-107-3p		MS00032235
mmu-miR-137-3p		MS00001589
mmu-miR-384-5p		MS00011963

Mature miRNA ID	company	miRNA Mimic Catalogue No.
mmu-miR-27a-3p	RealGene Labs	6130123
mmu-miR-27b-3p		6130006
mmu-miR-30a-5p		6130008
mmu-miR-30b-5p		6130010
mmu-miR-30c-5p		6130101
mmu-miR-30d-5p		6130100
mmu-miR-30e-5p		6130075
mmu-miR-103-3p		6130131
mmu-miR-107-3p		6130157
mmu-miR-137-3p		6130029
mmu-miR-140-5p		6130031
mmu-miR-146a-5p		6130038
mmu-miR-146b-5p		6130290
mmu-miR-192-5p		6130103
mmu-miR-215-5p		6130204
mmu-miR-384-5p		6130453

Primers used for rAAV qPCR titration

rAAV qPCR titration	forward primer (5' → 3')	reverse primer (5' → 3')
AAV-ITR	GGAACCCCTAGTGATGGAGTT	CGGCCTCAGTGAGCGA

Primers used for Cas9 based deletion

targeted gene	forward primer (5' → 3')	reverse primer (5' → 3')
Cyp2c70	TGGAAGAGAGGAGAGATGAT	ATATAATGCTCTCTTCTGGT
MUC1	ACATGTTATCCAAAGCGACCCA	GTAAACTGGAGTGGTGGCCAAG
MUC4	CCTCCTCACGTGACCAAATAC	TGACACCAGAAGTAGATGGCTT
MUC5ac	GGAGGGCATATCAGGGCTCTA	GTTTAGGGGGCAGCTCTAGTAT

Primers used for Chip-qPCR

gene symbol (mice)	chromatin position	forward primer (5' → 3')	reverse primer (5' → 3')
FXRE with the intron 1 of NFYC gene (Mus Musculus Chr4:120,661,717 - 120,663,486)	Chr4:120661694 - 120661890	ATTTGCCTATCTTTAATCCCA	GATTATAGACAGTTGTGAGC

Antibodies used for immunoblotting and immunoprecipitation

antibody	company	Catalogue No
βactin	Abcam	ab8226
albumin	CellSignaling	#4929
CAV1	Abcam	ab192869
CAVIN1	ThermoFisher	PA568780
CAVIN2	Bioss	BS19605R
CD73	Abcam	ab133582
Cyp2c70	Novus	NBP3-10165
laminB1	Abcam	ab16048
MUC1	Abcam	ab45167
MUC4	SantaCruz	sc33654
MUC5ac	St Johnslabs	STJ119455
PKCζ	Abcam	ab225554
PKCζ-pT410	CellSignaling	#2060
RBL1	Biorbyt	orb25528
RBL1-pT369	Biorbyt	orb100309
Sp1	Abcam	ab227383
Sp1-pT453	Abcam	ab59257

p, phosphorylation

Drugs used in this study

drug	company	Catalogue No
ceramide C16:0	Cayman	10681
ACPD	SigmaAldrich	R426911
GlyMCA	Tocris	6669
βMCA	Cayman	20287
CA	Cayman	20250
CDCA	Cayman	10011286
TαMCA	Cayman	20288
TβMCA	Cayman	20289
TωMCA	Cayman	28842
TCA	Cayman	16215
TCDCA	Cayman	20275
TDCA	Cayman	15935
TUDCA	Cayman	20277

Composite bile acid mixtures of 6 bile acids representing the major in vivo gallbladder bile acid species in mice^{WT} and mice$^{C70\text{-}KO}$

	WD (8-week)			
	none	none	GlyMCA treatment	TβMCA treatment
bile acid species	mice^{WT} (% bile acid)	mice$^{C70\text{-}KO}$ (% bile acid)	mice$^{C70\text{-}KO}$ (% bile acid)	mice$^{C70\text{-}KO}$ (% bile acid)
βMCA	3.1	0	0	7.2
TαMCA	3.5	0	0	0
TβMCA	34.1	0	0	34.2
TωMCA	11.2	0	0	15.3
CA	9.3	7.2	8.7	3.6
CDCA	0	5.2	4.4	0
TCA	38.8	27	32.6	14.7
TCDCA	0	55.4	49.4	25
TDCA	0	1.6	2	0
TUDCA	0	3.6	2.9	0

The valiated miR30 sponge sequence

actggtcgacTAGGTAACTGATtgccatgCTTCCAGTCGattTGTTTACAtcctAGCTGAGTGTAcagTGTTTACAagtaGCTGAGAGTGTcatTGTTTACAgtcaCTTCCAGTCGGcagTGTTTACAtgcgCTTCCAGTCAg
ccTGTTTACAaaatCTTCCAGTCGttgTGTTTACAttaaCTTCCAGTCAgcaTGTTTACAatagGCTGAGAGTGTcttTGTTTACAgactCTTCCAGTCGgcaATGTTTACAtggcCTTCCAGTCGAattTGTTTACAccatA
GCTGAGTGTtgcATGTTTACAtgacCTTCCAGTCGtaATGTTTACAtacaGCTGAGAGTGTctaATGTTTACAgttgCTTCCAGTCAcaaATGTTTACAgttaCTTCCAGTCGtcgTGTTTACAggatccCTTCCAGTCG
attATGTTTACAatccAGCTGAGTGTacaATGTTTACAaaggCTTCCAGTCAtccATGTTTACAttgtCTTCCAGTCGagtTGTTTACAgaatCTTCCAGTCGcatATGTTTACAgttggtcgacgaac

Table S5A. Serum ceramides of mice^{WT} and mice$^{C70\text{-}KO}$.

serum ceramides (nmol/g)	mice^{WT}		mice$^{C70\text{-}KO}$	
C16:0	1.1 (0.1664)	1.8 (0.0682)a	1.1 (0.1423)	1.8 (0.0899)a
C18:0	0.1 (0.0122)	0.1 (0.0054)a	0.1 (0.011)	0.1 (0.0054)a
C20:0	1.4 (0.209)	3.3 (0.1372)a	1.4 (0.2052)	3.4 (0.1406)a
C22:0	3 (0.3264)	5.1 (0.136)a	3 (0.5255)	5.1 (0.3146)a
C24:0	2.4 (0.3567)	4 (0.2995)a	2.3 (0.3938)	3.7 (0.244)a*
C24:1	0.6 (0.0948)	0.7 (0.0729)a	0.6 (0.089)	0.7 (0.0641)a
total	8.6 (1.0791)	15 (0.5539)a	8.6 (1.3209)	14.8 (0.6073)a
week	0	8	0	8
diet	WD			

Data exhibited as mean (S.D.). Individual *P* values were provided in Primary data for Table S5A.

a, significant differences between 0-week vs. 8-week.

, indicates significant differences between between each mice group (mice^{WT} vs. mice$^{C70\text{-}KO}$) at the same time point (, $P < 0.05$).

Table S5B. Serum ceramides of mice^{C70-KO}, under the treatment of either VEH or GlyMCA or TβMCA.

serum ceramides (nmol/g)	treatment		
	no treatment	GlyMCA	TβMCA
C16:0	1.8 (0.0899)a	1.3 (0.1478)a,###	1.8 (0.1492)a,ΔΔΔ
C18:0	0.1 (0.0054)a	0.1 (0.0106)a	0.1 (0.0097)a,###,ΔΔΔ
C20:0	3.4 (0.1406)a	1.7 (0.2254)a,###	2.4 (0.1983)a,#,Δ
C22:0	5.1 (0.3146)a	3.6 (0.3608)a,###	4.8 (0.305)a,ΔΔΔ
C24:0	3.7 (0.244)a	2.7 (0.3165)a,###	3.8 (0.3425)a,ΔΔΔ
C24:1	0.7 (0.0641)a	0.7 (0.0696)a	1 (0.0973)a,###,ΔΔΔ
total	14.8 (0.6073)a	10.2 (1.0274)a,###	13.9 (1.0237)a,ΔΔΔ
week	8		
diet	WD		

Data exhibited as mean (S.D.). Individual *P* values were provided in Primary data for Table S5B.

a, significant differences between 0-week (data shows in Table S5A) vs. 8-week.

Data with different symbol (#, GlyMCA or TβMCA treatment vs. no treatment; Δ, TβMCA vs. GlyMCA) indicates significant differences between each mice group at the same time point (#, $P < 0.05$; ###, $P < 0.001$; ΔΔΔ, $P < 0.001$).

Table S5C. Serum ceramides of mice$^{C70\text{-}KO}$, under the treatment of VEH or ACPD.

serum ceramides (nmol/g)	treatment	
	no treatment	ACPD
C16:0	1.8 (0.0948)a	1.4 (0.1438)a,###
C18:0	0.1 (0.0057)a	0.1 (0.0097)a
C20:0	3.4 (0.1482)a	1.8 (0.1551)a,###
C22:0	5.1 (0.3316)a	3.6 (0.2909)a,###
C24:0	3.7 (0.2572)a	2.8 (0.3852)a,###
C24:1	0.7 (0.0675)a	0.7 (0.0656)a
total	14.8 (0.6402)a	10.4 (0.8663)a,###
week	8	
diet	WD	

Data exhibited as mean (S.D.). Individual *P* values were provided in Primary data for Table 5C.

a, significant differences between 0-week (data shows in Table S5A) vs. 8-week.

#, indicates significant differences between between each mice group (no treatment vs. ACPD) at the same time point (###, $P < 0.001$).

Table S5D. Serum ceramides of mice$^{C70\text{-}KO}$, with i.p. injection of Blank or 30Spg.

serum ceramides (nmol/g)	treatment	
	Blank	30Spg
C16:0	1.8 (0.1141)	1.8 (0.0899)
C18:0	0.1 (0.004)	0.1 (0.0054)
C20:0	3.4 (0.185)	3.4 (0.1406)
C22:0	5.1 (0.238)	5.1 (0.3146)
C24:0	3.7 (0.2346)	3.7 (0.244)
C24:1	0.7 (0.0668)	0.7 (0.0641)
total	14.9 (0.4872)	14.8 (0.6073)
week	8	
diet	WD	

Data exhibited as mean (S.D.).

i.p. injection, intraperitoneal injection.

Blank, AAV-Null virus; 30Spg, AAV-30Spg virus.

Table S6. 8 potential Sp1 binding sites predicted by JASPAR, whose relative scores are more than 0.9, distribute from −80 to −2884 bp upstream of the transcription initiation site of mouse MUC1 gene (NC_000069.7:89133433-89136362).

Site	Sequence	Score	Relative Score	Strand
-2884/-2876	GGGGAGGAG	13.402603	0.951780455950466	-
-2834/-2826	GGGGAGGGG	14.750445	0.9752014461701994	+
-559/-551	GGGGCGGGG	16.177565	1.0000000061992012	+
-474/-466	GGGGAGGGA	12.432075	0.9349159145142774	+
-120/-112	GGGGAGGAG	13.402603	0.951780455950466	+
-98/-90	GGGGAGGGG	14.750445	0.9752014461701994	+
-92/-84	GGGGCGGAG	14.829722	0.9765790159794677	+
-94/-80	AAAACTCCGCCCCCT	13.728618	0.9183409333527228	-

Table S7. Clinical findings of human cholelithiasis patients (n = 7)

Variables	n (%)
Abdominal Pain	6 (85.7)
Vomiting	1 (14.3)
Dyspepsia	4 (57.1)
Jaundice	3 (42.9)
Palpable gallbladder	7 (100)
Murphy’s Sign	5 (71.4)
Leukocytosis	2 (28.6)
Hyperbilirubinemia	1 (14.3)

Leukocytosis, total leucocytes count more than 11,000/mm^3.

Hyperbilirubinemia, Total bilirubin >1.2 mg/dL.

Table S8. Mouse SDPR gene (ENSMUST00000051572.7) 3' UTR miRNA Table.

miRNA family	Total conserved sites	Conserved 8mer sites	Conserved 7mer-m8 sites	Conserved 7mer-1A sites	6mers	Aggregate Pct
miR-137-3p	1	1	0	0	0	0.74
miR-30-5p/384-5p	1	0	1	0	0	0.68
miR-27-3p	1	0	0	1	0	0.54
miR-103-3p/107-3p	0	0	0	0	2	0.23
miR-192-5p/215-5p	0	0	0	0	2	0.18
miR-140-5p	0	0	0	0	1	0.11
miR-146-5p	0	0	0	0	0	0.11

Table S9A. Effect of sgCyp2c70-mediated gene disruption in the model of miceC70-KO.

Intact sequence	GTGCATTTGTTCCCAgtgagtcaatggctctcttcATCTTTCTGGGGATTTGGCTTTCTTGTTTTCTTTTTCTTTTTCTATggaatcagcaccgtggcagaGGGAAGCT (sgCyp2c70)

The PAM sites were underlined. The sgCyp2c70-1 sites or the sgCyp2c70-2 sites were marked with blue lowercase font.

Table S9B. Effect of sgMUC1-mediated gene disruption in the model of miceC70-KO.

Intact sequence	CAAGTGAGGAAAACAGTGTCACCTCATCTCAGGACACCAGCAGTtccttagcatcgactaccacTCCAGTCCACAGCAGCAACTCAGAC (sgMUC1-1)
	TAGCACTCCAGACCACTCAGTCACAACTACCAGCTCTGCACTGGgctcagccaccagtccagacCACAGTGGTACCTCAACTACAACTA (sgMUC1-2)

The PAM sites were underlined. The sgMUC1-1 sites or the sgMUC1-2 sites were marked with blue lowercase font.

Table S9C. Effect of sgMUC4-mediated gene disruption in the model of miceC70-KO.

Intact sequence	GGACACACAACAGCAATAACAACTCAAggtttaacacctgccaccgcacaagtctcactgaCACCTTCATCCCAGAATATGTCAACAGTGTCAACACCCATCAC (sgMUC4)

The PAM sites were underlined. The sgMUC4-1 sites or the sgMUC4-2 sites were marked with blue lowercase font.

Table S9D. Effect of sgMUC5ac-mediated gene disruption in the model of miceC70-KO.

Intact sequence	TGGTACGAGCCTTCAACCCAGgacataccaggcgggtgtgcAGCACATGGGGCAACTTCCACTACAAGACCTTTGATGGACAGGTCTT (sgMUC5ac-1)
	CTAGTTGTTGAGCTGACCAAGagctccgtcttagtcaataaCCACCCGGTCCAGCTGCCCTTTAGCCAGTCTGGGGTCCTCATTGAGCT (sgMUC5ac-2)

The PAM sites were underlined. The sgMUC5ac-1 sites or the sgMUC5ac-2 sites were marked with blue lowercase font.

Table S10. Diet composition.

	Experimental Diet Composition					
	chow (%)		WD* (%)		LD# (%)	
	kcal	kcal/g	Kcal	kcal/g	kcal	kcal/g
Protein	23		20		20	
Fat	12		60		60	
Carbohydrate	65		20		20	
total	100	3.7	100	5.4	100	5.4
	weight %		weight %		weight %	
Casein	20		20		20	
Corn Starch	45.9		26.7		26.7	
Lard	0		20		20	
Sucrose	5		5		5	
Cellulose	5		5		5	
Soy oil	5		5		5	
Maltodextrin	5.6		5.6		5.6	
Mineral	6		6		6	
Vitamin	1.2		1.2		1.2	
Cholesterol	0.02		1		1	
Cholic acid	0		0		0.5	

*: WD, western diet; #: LD, lithogenic diet.

Table S11A. Serum ALT and AST levels of mice$^{C70\text{-}KO}$, supplemented with the treatment of the drinking water containing vitamin supplements.

	mice^{WT}	mice$^{C70\text{-}KO}$	
	water/vitamin[a]	water	water/vitamin[a]
Serum ALT (U/L)	75.7 (26.8)***	299.4 (95.2)##	151.8 (25.5)
Serum AST (U/L)	162 (37.5)***	706.8 (206.1)###	286.7 (42.6)

ALT, alanine aminotransferase; AST, aspartate aminotransferase.

a, the distilled water containing vitamin supplements.

Data with different lowercase letter (*, mice^{WT} vs. mice$^{C70\text{-}KO}$; #, mice$^{C70\text{-}KO}$ supplemented with the distilled water vs mice$^{C70\text{-}KO}$ supplemented with the distilled water containing vitamin supplements.) are significantly different between each mice group. ***, $P < 0.001$; ##, $P < 0.01$; ###, $P < 0.001$.

Individual *P* value were provided in Primary data for Table S11A.

Table S11B. Serum ALT and AST levels of mice$^{C70\text{-}KO}$ after 7 days treatment with GlyMCA or with TβMCA.

	mice$^{C70\text{-}KO}$/GlyMCA			mice$^{C70\text{-}KO}$/TβMCA		
	0 mg/kg	10 mg/kg	50 mg/kg	0 mg/kg	200 mg/kg	500 mg/kg
Serum ALT (U/L)	151.8 (25.5)	158 (25.9)	161.6 (31.2)	151.8 (25.5)	159.5 (37.5)	158.9 (24.5)
Serum AST (U/L)	286.7 (42.6)	291.3 (26)	283.4 (46.1)	286.7 (42.6)	274.4 (34)	280.9 (53.4)

ALT, alanine aminotransferase; AST, aspartate aminotransferase.

Supplementary Materials

Generation of Cyp2c70-Knockout (C70-KO) mice

A pair of single guide RNA (sgRNA): sgCyp2c70-1 (5'-GGAATCAGCACCGTGGCAGA-3') and sgCyp2c70-2 (5'-GAAGAGAGCCAT-TGACTCAC-3') targeting exon 1 of the murine Cyp2c70 gene (OTTMUST00000094546.1) were designed by an Optimized CRISPR Design tool (http://www.e-crisp.org).

Addgene plasmid #64073, a gift from Andrea Ventura[1], was digested with XbaI and KpnI to gel extracted the secondary U6:sgRNA unit. The digestion products were gel-purified and were subsequently recombined into the digested PX462 vector [Addgene plasmid #62987, Streptococcus pyogenes (Sp) Cas9 D10A nickase mutant (SpCas9n), a gift from Feng Zhang[2]] to form the U6:sgRNA/U6:sgRNA-CMV-SpCas9n construct (Supplementary Figure 22A, left panel). All constructs were confirmed by sequencing.

The two sgCyp2c70-1 and sgCyp2c70-1 were cloned into the U6:sgRNA/U6:sgRNA-CMV-SpCas9n, then was linearized by the restriction digestion using NotI followed by gel extraction and purification using a Wizard® SV Gel and PCR Cleanup System (A9282, Promega, Madison, WI). The sample was diluted to 2 ng/µl and injected into the zygotes of C57BL/6 mice. The third generation (F3) mice were identified by ear biopsy genotyping (Supplementary Figure 22B and 22C; Supplementary Table 7A).

Viral-based CRISPR-Staphylococcus aureus (Sa) Cas9:sgRNA delivery

Dual-sgMUC1: sgMUC1-1 (5'-TCCTTAGCATCGACTACCAC-3') and sgMUC1-2 (5'-GCTCAGCCACCAGTCCAGAC-3') targeting exon 2 of the murine MUC1 gene (OTTMUST00000048120.1); dual-sgMUC4: sgMUC4-1 (5'-TCAGTGAGACTTGTGCGGTG-3') and sgMUC4-2 (5'-GGTTTAACACCTGCCACCGC-3') targeting exon 2 of the murine MUC4 gene (OTTMUST00000062066.3); dual-sgMUC5ac: sgMUC5ac-1 (5'-GCACACCCGCCTGGTATGTC-3') and sgMUC5ac-2 (5'-TTATTGACTAAGACGGAGCT-3') targeting exon 4 of the murine MUC5ac

gene (OTTMUST00000084845.3) were designed by an Optimized CRISPR Design tool (http://crispor.tefor.net/).

We used a plasmid called PX333, which is a gift from Alex Hewitt (Addgene plasmid #64073[1]) that contains the secondary U6:sgRNA unit. By using a technique called site-directed mutagenesis, we mutated an unwanted XbaI restriction site at 497 bp in the PX333 vector to an Acc65I restriction site. The PX601 vector (a gift from Feng Zhang; Addgene plasmid # 61591[3]) with a saCas9^{KKH} (E782K/N968K/R1015H) mutant (PX601-saCas9^{KKH} vector) was synthesized by GenerayBiotech (GenerayBiotech, Shanghai, China). Then the PX333 plasmid with the mutated Acc65I restriction site was digested with Acc65I and KpnI restriction enzymes, and the secondary U6:sgRNA unit was gel-purified. The U6:sgRNA unit was subsequently recombined into the digested PX601-saCas9^{KKH} vector, which was also digested with Acc65I and KpnI restriction enzymes, to form the PX601-saCas9^{KKH}-U6:sgRNA/ U6:sgRNA construct. All constructs were confirmed by sequencing.

As described above, the Cas9 enzyme and the sgRNAs (sgCTRL, sgMUC1, sgMUC4, sgMUC5ac) were introduced into the 293T cells using PX601-saCas9^{KKH}. The AAV2/8 serotype packaging plasmid, pAAV8 and pALD-X80 AAV Helper plasmid were used to help produce and package the AAV particles. Viral genome particles were titered by real-time quantitative PCR (Supplementary Table 3).

12-week-old mice$^{C70\text{-}KO}$ were anesthetized by intraperitoneal injection of pentobarbital sodium (40 mg/kg) followed by intraperitoneal injection (2×10^{12} viral genome particles/mouse) of either a control non-targeting sgRNA (AAV-Cas9-sgCTRL) or specific sgRNA (AAV-Cas9-sgMUC1, AAV-Cas9-sgMUC4, or AAV-Cas9-sgMUC5ac) using a 32-gauge needle (Supplementary Figure 5A-C; Supplementary Table 7B-D).

Viral-baed MUC1 Overexpression and miR30 knockout

For construction of viral-baed MUC1 overexpression (MUC1-OE) and microRNA-30 (miR30) knockout, the previously validated murine MUC1 mRNA coding sequence (CCDS17496.1) and miR30 Sponge (30Spg) sequence[4] (Supplementary Table 3)

was synthesized by GenerayBiotech (GenerayBiotech, Shanghai, China) and cloned into the adeno-associated virus (AAV) Type 2/8 (AAV2/8) viral system. And AAV2/8-CMV-MUC1 (AAV-MUC1, MUC1-OE), AAV2/8-CMV-30Spg (AAV-30Spg, 30Spg) and AAV2/8-CMV- Null (AAV-Null, Blank) virus were packaged by Obio Technology (Obio, Shanghai, China). 12-week-old mice were anesthetized by intraperitoneal injection of pentobarbital sodium (40 mg/kg) followed by intraperitoneal injection of $^{AAV2/8}$MUC1, $^{AAV2/8}$30Spg or $^{AAV2/8}$Null control (2×10^{12} viral genome particles/mouse) using a 32-gauge needle (Supplementary Figure 5A, 5D and 5E).

Hepatic changes in mice$^{C70\text{-}KO}$ were rescued by vitamin supplement

The BA pool of mice$^{C70\text{-}KO}$ show no change in BA pool size but was more hydrophobic than mice^{WT} (Supplementary Figure 1A and 1B). This phenomenon was ascribed to a loss of Cyp2c70 expression that would fail to initiate the conversion of the hydrophobic CDCA into the hydrophilic MCA (Supplementary Figure 1C). The elevation of the potent FXR agonist CDCA levels in mice$^{C70\text{-}KO}$ could statistically downregulate the mRNA expression of the BA biosynthetic enzymes (Cyp7a1/8b1) and the BA transporters (NTCP and OATP1a1) in the liver, but elevate the mRNA expression of the endogenous source of LXR ligand (Ch25OH) and the bilirubin conjugated enzyme (UGT1a1) in the liver, and SHP, CCL2, CXCL9 and IL6 in the gllbladder (Supplementary Figure 23A-D). Moreover, although liver histology (H&E and Masson’s trichrome staining) did not reveal signs of hepatic injury and active inflammation in mice$^{C70\text{-}KO}$ (Supplementary Figure 1D), the concentrations of liver damage markers (ALT and AST) in plasma were higher in mice$^{C70\text{-}KO}$, in comparison with that of mice^{WT} (Supplementary Table 10A). These data were in consistent with previous reports suggesting that the exposure of “novel” CDCA in mice$^{C70\text{-}KO}$ might cause liver injury via either “its associated inflammatory signals” (e.g., CDCA-JNK signaling) or the chronic FXR activation[5]. We found that vitamin supplement in drinking water was effective at restoring hepatic Cyp7a1/8b1 mRNA expression in mice$^{C70\text{-}KO}$, which could ascribed to the decreased SHP expression due to elevated vitamin D receptor (VDR) funtion[6] in the liver of mice$^{C70\text{-}KO}$ (Supplementary

Figure 1E and 1F, Supplementary Figure 23A and 23B). However, vitamin supplementation had a mildly effect on the mRNA expression of SHP in the gallbladder of mice$^{C70\text{-}KO}$ (Supplementary Figure 23A), which was attributed to the fact that gallbladder filling during fasting has the advantage of a relatively long time activation of FXR by CDCA. Vitamin supplementation also attenuated the levels of serum ALT and AST (Supplementary Table 10A). Further studies are required to clarify the mechanisms underlying the protective results of vitamin mixture treatment on the hydrophobic BA induced liver damage of mice$^{C70\text{-}KO}$.

The optimal dose of the GlyMCA, TβMCA on mice$^{C70\text{-}KO}$

We investigated the optimal dose of two compounds, Gly-MCA and TβMCA, for attenuating the activity of FXR in the small intestine (SI) and gallbladder of mice$^{C70\text{-}KO}$. The dough pills were produced for the oral administration of Gly-MCA (dose of 0, 10 and 50 mg/kg) or TβMCA (dose of 0, 200 and 500 mg/kg). Oral GlyMCA or TMCA did not cause any additional liver toxicity in mice$^{C70\text{-}KO}$ at any of the doses tested, as revealed by serum ALT and AST (Supplementary Table 10B). The SHP and FGF15 mRNAs in the SI of mice$^{C70\text{-}KO}$ were maximum decreased at a dose of 50 mg/kg GlyMCA (up to 38.7% and 42.2%, respectively) or a dose of 500 mg/kg TβMCA (up to 68% and 66.5%, respectively) for 7 days compared with other doses (Supplementary Figure 24A and 24B). As expected[7], GlyMCA levels in the gallbladder bile were undetectable (Supplementary Figure 18A). And we observed that SHP mRNA in the gallbladder of mice$^{C70\text{-}KO}$ were decreased (up to 68.9%) under the treatment of TβMCA (500 mg/kg) compared with other doses and with GlyMCA-treated mice$^{C70\text{-}KO}$ (Supplementary Figure 24A and 24B).

Ceramide determination

For serum lipidomics, a 25 μl sample aliquot of serum from experimental mice was extracted with ice-cold chloroform:methanol (2:1, v/v; 100 μl) solution containing ceramide d18:1-C12:0 and sphingomyelin d18:1-C12:0 (Avanti, Alabaster, AL) at 2 μM as internal standards. The sample was vibrated for 30 seconds and then incubated for 5 minutes at room temperature. After centrifugation (13,000 g, 5 minutes; Optima XPN-100, BeckmanCoulter, Brea, CA), the organic phase was collected and

dried under vacuum at room temperature. The residue was dissolved in 25 μl of chloroform:methanol (1:1), followed by diluting with isopropanol:acetonitrile:H_2O (2:1:1, v/v/v) containing 2 μM 1,2-diheptadecanoyl-sn-glycero-3-phosphocholine 17:0 (850360, Avanti, Alabaster, AL) before by ultra-performance liquid chromatography coupled with tandem quadrupole mass spectrometry (Waters, Milford, MA). Total ceramide contents were calculated from the sum of individual ceramide species, and their amounts were normalized to the internal standards.

Quantification of bile acid metabolites

TCA-d5 (T008852, TRC, North York, Canada) was used as an internal standard. And the concentrations of bile acid (BA) metabolites were determined by ultra-performance liquid chromatography coupled with tandem quadrupole mass spectrometry (Waters, Milford, MA) with multiple reaction monitoring in negative mode. A mass range of m/z 50 ~ 850 amu at a rate of 0.3 second/scan was acquired. The column temperature was maintained at 45 °C, and the flow rate was 0.4 mL/minute. Tissues (the liver, ileum, and gallbladder) were weighed, and then were homogenized with 100% acetonitrile containing internal standard. After centrifugation (13,000 g, 25 minutes; Optima XPN-100, BeckmanCoulter, Brea, CA), the supernatant was diluted by an equal volume of HPLC grade water containing 0.1% formic acid. UPLC was performed as described following the methods of Takahashi: (A) 0.1% formic acid in water; (B) 0.1% formic acid in acetonitrile. The gradient elution was started from 80% (A) for 4 minutes, decreased linearly to 60% (A) at 15 minutes, to 40% (A) at 20 minutes, to 10% (A) at 21 minutes, followed by flushing for 1-minute, and finally increased to 80% (A) for 4 minutes to re-equilibrate the column.

In vitro lentiviral particle transduction, drug treatment and qRT-PCR

Here we used the cultured human intrahepatic biliary epithelial cell (HIBEpiC) lines as an *in vitro* model for studying the regulation of the expression of MUC1 in gallbladder epithelial cells (GBEC). HIBEpiC lines were obtained from ScienCell Research Laboratories (5100, ScienCell, Carlsbad, CA). The cells were grown as monolayers at 37°C in a humidified atmosphere of 95% air and 5% CO_2 in Epithelial

Cell Medium (EpiCM; 4101, ScienCell, Carlsbad, CA) supplemented with 10% fetal bovine serum (FBS; ThermoFisher, Waltham, MA).

The pMD19T vectors containing the sequence of SDPR protein coding sequence (CDS) region (Homo Sapiens CCDS2313.1) and the sequence of the site-directed mutagenesis (T369E; Supplementary Figure 12F) of RBL1 CDS region (Homo Sapiens CCDS13289.1) were stored by our laboratory and were respectively inserted into the pCDH-GFP plasmid (CD511B-1, System Biosciences, Palo Alto, CA). The pCDH-GFP plasmid was used as blank control.

The sequence of Sp1 shRNA (5'-GCTGGTGGTGATGGAATACAT-3'), the sequence of RBL1 shRNA (5'-AGTTGGAGCTCTGTCCTTCAA-3'), the sequence of SDPR shRNA (5'-CAGCAACATCGACTTGACTAT-3'), and the sequence of CTRL shRNA (5'-GCAGTTATCTGG- AAGATCAGG-3') were synthesized by GenerayBiotech, China (GenerayBiotech, Shanghai, China) and were respectively inserted into the pLL3.7-GFP plasmid (Addgene plasmid #11795, a gift from Luk Parijs[8]).

The lentiviral Sp1 shRNA (^{lenti}shSp1), ^{lenti}shRBL1, ^{lenti}shSDPR, ^{lenti}shCTRL, and the lentiviral transgenesis of SDPR ($^{lenti\text{-}Tg}$SDPR), $^{lenti\text{-}Tg}$RBL1-T369E, $^{lenti\text{-}Tg}$blank were packaged by Obio Technology (Obio, Shanghai, China).

HIBEpiC lines stably expressing exogenous blank control, the wild type SDPR protein, the site-directed mutagenesis of threonine 369 to glutamicacid (T369E) in RBL1 protein, Sp1 shRNA, RBL1 shRNA and scrambled control (CTRL) shRNA were generated using lentivirus transduction. Briefly, $1x10^6$ HIBEpiC lines were seeded in 6-well plates 1 day before transduction. The cells were transduced with $1x10^8$ IFU in 2 mL of culture medium. 7 days later, we selected the stably transfected cells by using a MoFlo Cytomation (BeckmanCoulter, Brea, CA) fluorescence-activated cell sorter to sort the cells for GFP expression. After sorting for insertion, the cells were cultured in a totally renewed cell culture medium and treated with indicated dose (0 μM to 10 μM) of C16:0 ceramide or with indicated dose (0 μM to 10 μM) of ACPD and 5 μM C16:0 ceramide for 2 hour. In some experiments, the cells were pretreated for 72 hours with different reagents before C16:0 ceramide treatment, such as ^{Hpho}BA or ^{Hphil}BA mixture (0 μM to 100 μM; Supplementary Table

3), CDCA (50 μM), GW4064 (2 μM to 5 μM), GlyMCA (25 μM to 100 μM), TβMCA (100 μM to 300 μM).

The mRNA levels of MUC1 were examined by qRT-PCR using total RNA extracted from *in vitro* GBEC model (HIBEpiC lines). All chemical reagents and primer sequences were shown in Supplementary Table 3. Expression data were normalized to the expression of 18s RNA.

Luciferase assay

The full length (FL) MUC1 gene promoter (MUC1-LUC-FL; Mus Musculus Chr3:89133433 ~ 89136333, in the region upstream of the initiation site of mouse MUC1 gene), and various shorter fragments of the MUC1 gene promoter, such as MUC1-LUC-DM1 (Mus Musculus Chr3:89133433 ~ 89133732), MUC1-LUC-DM2 (Mus Musculus Chr3:89135663 ~ 89135962), MUC1-LUC-DM3 (Mus Musculus Chr3:89136064 ~ 89136333), MUC1-LUC-120/112 (Mus Musculus Chr3:89136064 ~ 89136333), MUC1-LUC-98/80 (Mus Musculus Chr3:89136063 ~ 89136264 and Chr3:89136284 ~ 89136333), were inserted into the pGreenFire 2.0 mCMV luciferase reporter (TR411PA-P, System Biosciences, Palo Alto, CA). Similarly, a fragment of the FXR gene (FXR-LUC; Mus Musculus Chr4:120661717 ~ 120663486, in the intron 1 region of mouse NFYC gene) and a full 3’UTR of human SDPR gene (SDPR-LUC; Homo Sapiens Chr2:192699028 ~ 192700648, in the region downstream of the stop codon of human SDPR gene) were also inserted into the pGreenFire 2.0 mCMV luciferase reporter (TR411PA-P, System Biosciences, Palo Alto, CA). The pGreenFire plasmid containing a 1770 bp DNA fragment in the intron 1 region of mouse NFYC gene (Mus Musculus Chr4:120661717 ~ 120663486) was used as a template to generate a FXR-mutated (FXR-LUC-MT) binding site construct by using site-directed mutagenesis. This mutation involved changing a putative FXR binding site 5’-TGGACTCCAGGTCAGTGAGAA-3’ (Mus Musculus Chr4:120661800 ~ 120661820) to 5’-TAAAAACCAAAAA- TGTAAAAA-3’ on the construct. The pGreenFire plasmid containing a 1621 bp DNA fragment in the region downstream of the stop codon of human SDPR gene (Homo Sapiens Chr2:192699028 ~ 192700648) was used as a template to generate a SDPR-mutated (SDPR-MT)

binding site construct by using site-directed mutagenesis. This mutation involved changing a putative FXR binding site 5'-ACAAAUG-3' (Homo Sapiens Chr2: 192700114 ~ 192700120) to 5'-GGGGGGG-3' on the construct. Mutations were created by site-directed mutagenesis using the GeneArt™ Site-Directed Mutagenesis System (ThermoFisher A13282, Waltham, MA). These mutations were sequence verified. These constructs were synthesized by GenerayBiotech (GenerayBiotech, Shanghai, China) and then packaged into lentivirus by Obio Technology (Obio, Shanghai, China).

The HIBEpiC lines were transduced with various lentivirus respectively, including: MUC1-LUC-WT, MUC1-LUC-DM1, MUC1-LUC-DM2, MUC1-LUC-DM3, MUC1-LUC-120/112, MUC1-LUC-98/80, FXR-LUC, FXR-LUC-MT, SDPR-LUC, and SDPR-MT. After 24 hours following lentiviral transduction, the cells were cultured in a totally renewed cell culture medium and treated with indicated dose (0 μM to 10 μM) of C16:0 ceramide or with indicated dose (0 μM to 10 μM) of ACPD and 5 μM C16:0 ceramide for 2-hour. In some experiments, the cells were pretreated for 72 hours with different reagents before C16:0 ceramide treatment, such as ^{Hpho}BA or ^{Hphil}BA mixture (0 μM to 100 μM; Supplementary Table 3), CDCA (50 μM), GW4064 (2 μM to 5 μM), GlyMCA (25 μM to 100 μM), TβMCA (100 μM to 300 μM).

The luciferase activities were detected using a Dual-Glo Luciferase Reporter Assay System kit (E2920, Promega, Madison, WI), and luciferase light emission was monitored on a luminometer (Synergy HTX, BioTek, Winooski, VT). Luminescence was normalized to cell number from viable cell counting by Cellometer reading (Nexcelom Bioscience, Lawrence, MA).

miRNA expression analysis

Mice^{WT} and mice$^{C70\text{-}KO}$ were anesthetized after 12-week chow or WD feeding, total RNA from the gallbladder tissues were extracted with TRIzol (ThermoFisher, Waltham, MA). cDNA was synthesized from 1 μg of total RNA using the TaKaRa RNA PCR Kit (Takara, Shiga, Japan). cDNA was used as a template for qRT-PCR amplification to observe the miRNAs expression by using the miRNAs-specific

primers (Qiagen, Hilden, Germany; Supplementary Table 3) for mmu-miR-27b-3p (miR27b), mmu-miR-30a-5p (miR30a), mmu-miR-30b-5p (miR30b), mmu-miR-30c-5p (miR30c), mmu-miR-30d-5p (miR30d), mmu-miR-30e-5p (miR30e), mmu-miR-107-3p (miR107), mmu-miR-137-3p (miR137), and mmu-miR-384-5p (miR384). The primers used to amplify 18s rRNA were 5'-GATGGGAAGTACAGCCAGGT-3' (forward) and 5'-TTTCTTCAGCCTCTCCAGGT-3' (reverse). All reactions were performed in triplicate. The relative expression of miRNAs was normalized to U6. Data were analyzed by using the $2^{-\Delta\Delta}$Ct method.

The mmu-miR-27a-3p (Assay ID MC10939), mmu-miR-27b-3p (Assay ID MC10750), mmu-miR-30a-5p (Assay ID MC11062), mmu-miR-30b-5p (Assay ID MC10986), mmu-miR-30c-5p (Assay ID MC11060), mmu-miR-30d-5p (Assay ID MC10756), mmu-miR-30e-5p (Assay ID MC10037), mmu-miR-103-3p (Assay ID MC10632), mmu-miR-107-3p (Assay ID MC10056), mmu-miR-137-3p (Assay ID MC10513), mmu-miR-140-5p (Assay ID MC10205), mmu-miR-146a-5p (Assay ID MC10722), mmu-miR-146b-5p (Assay ID MC10105), mmu-miR-192-5p (Assay ID MC10456), mmu-miR-215-5p (Assay ID MC10296), and mmu-miR-384-5p (Assay ID MC12637) were designed and synthesised by RealGene Labs (RealGene Labs, Lake Forest, CA). For the analysis of the miRNA-SDPR axis, HIBEpiC lines were grown to approximately 60% confluence in 24-well plates and co-transfected with different miRNA mimics (100 nM). After 48-hour of transfection, total RNA from the HIBEpiC lines (non-transfected HIBEpiC lines were used as control) were extracted with TRIzol (ThermoFisher, Waltham, MA). cDNA was synthesized from 1 μg of total RNA using the TaKaRa RNA PCR Kit (Takara, Shiga, Japan). cDNA was used as a template for qRT-PCR amplification to observe the SDPR mRNA expression. Significantly and differentially expressed SDPR mRNA by various miRNA mimics were identified by volcano plot filtering (GraphPad Software, San Diego, CA).

Reference

[1] D. Maddalo, E. Manchado, C. P. Concepcion, C. Bonetti, J. A. Vidigal, Y. C. Han, P. Ogrodowski, A. Crippa, N. Rekhtman, E. de Stanchina, S. W. Lowe, A. Ventura, *Nature* **2014**, *516* (7531), 423, https://doi.org/10.1038/nature13902.

[2] F. A. Ran, P. D. Hsu, J. Wright, V. Agarwala, D. A. Scott, F. Zhang, *Nat Protoc* **2013**, *8* (11), 2281, https://doi.org/10.1038/nprot.2013.143.

[3] F. A. Ran, L. Cong, W. X. Yan, D. A. Scott, J. S. Gootenberg, A. J. Kriz, B. Zetsche, O. Shalem, X. Wu, K. S. Makarova, E. V. Koonin, P. A. Sharp, F. Zhang, *Nature* **2015**, *520* (7546), 186, https://doi.org/10.1038/nature14299.

[4] J. Wu, C. Zheng, Y. Fan, C. Zeng, Z. Chen, W. Qin, C. Zhang, W. Zhang, X. Wang, X. Zhu, M. Zhang, K. Zen, Z. Liu, *J Am Soc Nephrol* **2014**, *25* (1), 92, https://doi.org/10.1681/ASN.2012111101.

[5] a) G. L. Guo, J. Y. L. Chiang, *J Lipid Res* **2020**, *61* (3), 269, https://doi.org/10.1194/jlr.C120000621; b) Q. Cheng, Y. Inaba, P. Lu, M. Xu, J. He, Y. Zhao, G. L. Guo, R. Kuruba, R. de la Vega, R. W. Evans, S. Li, W. Xie, *Mol Endocrinol* **2015**, *29* (4), 571, https://doi.org/10.1210/me.2014-1337.

[6] E. C. Chow, L. Magomedova, H. P. Quach, R. Patel, M. R. Durk, J. Fan, H. J. Maeng, K. Irondi, S. Anakk, D. D. Moore, C. L. Cummins, K. S. Pang, *Gastroenterology* **2014**, *146* (4), 1048, https://doi.org/10.1053/j.gastro.2013.12.027.

[7] C. Jiang, C. Xie, Y. Lv, J. Li, K. W. Krausz, J. Shi, C. N. Brocker, D. Desai, S. G. Amin, W. H. Bisson, Y. Liu, O. Gavrilova, A. D. Patterson, F. J. Gonzalez, *Nat Commun* **2015**, *6*, 10166, https://doi.org/10.1038/ncomms10166.

[8] D. A. Rubinson, C. P. Dillon, A. V. Kwiatkowski, C. Sievers, L. Yang, J. Kopinja, D. L. Rooney, M. Zhang, M. M. Ihrig, M. T. McManus, F. B. Gertler, M. L. Scott, L. Van Parijs, *Nat Genet* **2003**, *33* (3), 401, https://doi.org/10.1038/ng1117.
